# Supplementary material for: Basal T cell activation predicts yellow fever vaccine response independently of cytomegalovirus infection and sex-related immune variations
Source: Cell Rep Med. 2025 Feb 11;6(2):101946. doi: 10.1016/j.xcrm.2025.101946 (PMC11866508; doi:10.1016/j.xcrm.2025.101946)
Supplement: Document S1. Figures S1–S12 [file mmc1.pdf]

Cell Reports Medicine, Volume *6*

## Supplemental information

# Basal T cell activation predicts yellow fever vaccine response independently of cytomegalovirus infection and sex-related immune variations

**Antonio Santos-Peral, Magdalena Zaucha, Elena Nikolova, Ekin Yaman, Barbara Puzek, Elena Winheim, Sebastian Goresch, Magdalena K. Scheck, Lisa Lehmann, Frank Dahlstroem, Hadi Karimzadeh, Julia Thorn-Seshold, Shenzhi Jia, Fabian Luppa, Michael Pritsch, Julia Butt, Camila Metz-Zumaran, Giovanna Barba-Spaeth, Stefan Endres, Sarah Kim-Hellmuth, Tim Waterboer, Anne B. Krug, and Simon Rothenfusser**

| *Pathogen family or species* | *Type* | *Antigen* | *Cohort prevalence (n = 248)*[a] | *Male n = 80* | *Female n = 168* |
|---|---|---|---|---|---|
| *Papillomaviridae* | **HPV 1** | L1 | 70 (28.23 %) | 15 | 55 |
| | **HPV 4** | L1 | 34 (13.71 %) | 5 | 29 |
| | **HPV 8** | L1 | 26 (10.48 %) | 5 | 21 |
| *Polyomaviridae* | **JCPyV** | VP1 | 110 (44.35 %) | 41 | 69 |
| | **KIPyV** | VP1 | 163 (65.73 %) | 56 | 107 |
| | **WUPyV** | VP1 | 240 (96.77 %) | 77 | 163 |
| | **HPyV 6** | VP1 | 202 (81.45 %) | 65 | 137 |
| | **HPyV 7** | VP1 | 113 (45.56 %) | 37 | 76 |
| | **MCV** | VP1 | 178 (71.77 %) | 60 | 118 |
| *Herpesviridae* | **HHV 1 or HSV1** | gG | 124 (50.00 %) | 40 | 84 |
| | **HHV 2 or HSV2** | mgG | 17 (6.85 %) | 5 | 12 |
| | **HHV 3 or VZV** | gE gI | 145 (58.47 %) | 46 | 99 |
| | **HHV 4 or EBV** | | 210 (84.68 %) | 64 | 146 |
| | | Zebra | 198 (79.84 %) | 61 | 137 |
| | | EA D | 157 (63.31 %) | 46 | 111 |
| | | VCA p18 | 186 (75.00 %) | 57 | 129 |
| | | EBNA | 206 (83.06 %) | 65 | 141 |
| | **HHV 5 or CMV** | | 83 (33.47 %) | 25 | 58 |
| | | pp150 | 91 (36.69 %) | 27 | 64 |
| | | pp52 | 77 (31.05 %) | 21 | 56 |
| | | pp28 | 87 (35.08 %) | 26 | 61 |
| | **HHV 6** | | 70 (28.23 %) | 18 | 52 |
| | | IE1B | 58 (23.39 %) | 13 | 45 |
| | | IE1A | 21 (8.47 %) | 7 | 14 |
| | **HHV 7** | U14 | 187 (75.40 %) | 59 | 128 |
| *Toxoplasma gondii* | **Tg** | | 37 (14.92 %) | 11 | 26 |
| | | p22 | 7 (2.82 %) | 2 | 5 |
| | | Sag 1 | 31 (12.50 %) | 9 | 22 |
| *Chlamydia trachomatis* | **Ct** | Pgp3 | 35 (14.11 %) | 6 | 29 |
| *Mycoplasma genitalium* | **Mg** | | 6 (2.42 %) | 3 | 3 |
| | | MgPa | 19 (7.66 %) | 6 | 13 |
| | | rMgPa | 22 (8.87 %) | 10 | 12 |
| *Parvoviridae* | **B19** | VP1 | 175 (70.56 %) | 57 | 118 |

**Figure S1. Prior infection status of the study cohort**
Cohort prevalence of 20 common pathogen infections including viruses, bacteria, and protozoans.
[a]2 individuals were not available for serology analysis

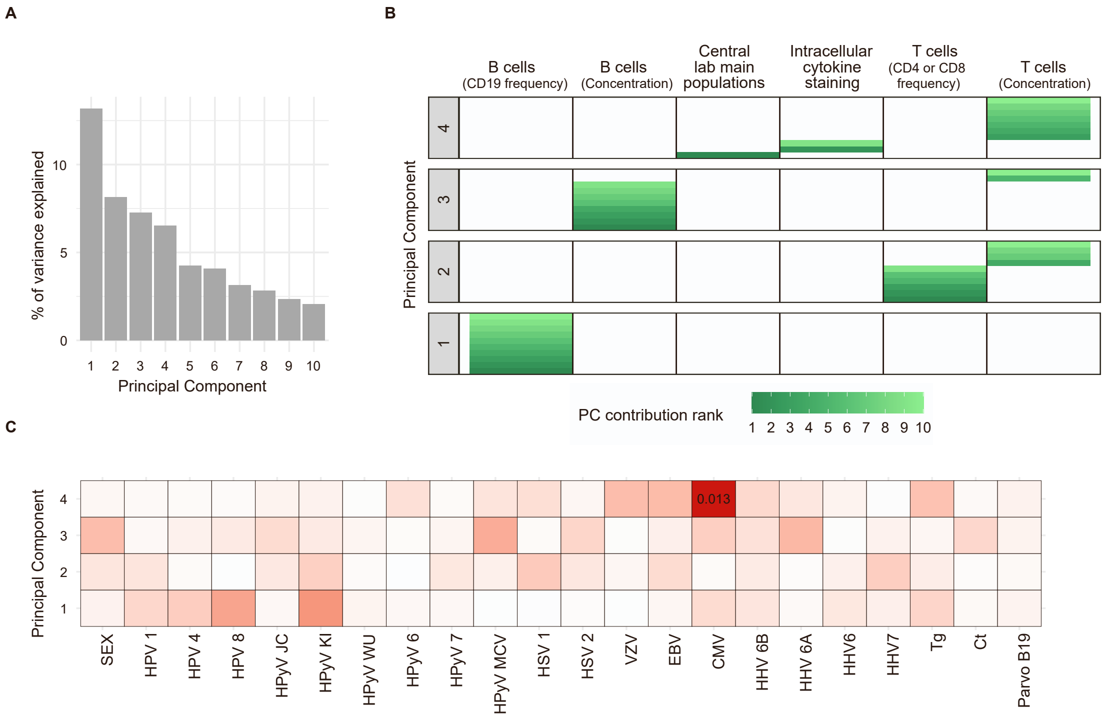


**Figure S2. Principal component analysis of baseline parameters.** Related to Figures 1 and 2 and Table S1.

A) Scree plot illustrating the proportion of variance explained by the top 10 principal components (PCs). B) Key contributors to the first four PCs, grouped by baseline parameter categories. PCs 1 and 3 are primarily influenced by B-cell-related measurements, while PCs 2 and 4 are largely driven by T-cell-related measurements. Notably, PC4 also captures variability related to lymphocyte counts. C) Association between baseline PCs and metadata categories. The mean of PC4 values differs significantly between CMV-positive and CMV-negative individuals (t-test, adj. p-value = 0.013). Baseline parameters are listed in supplementary table S1.

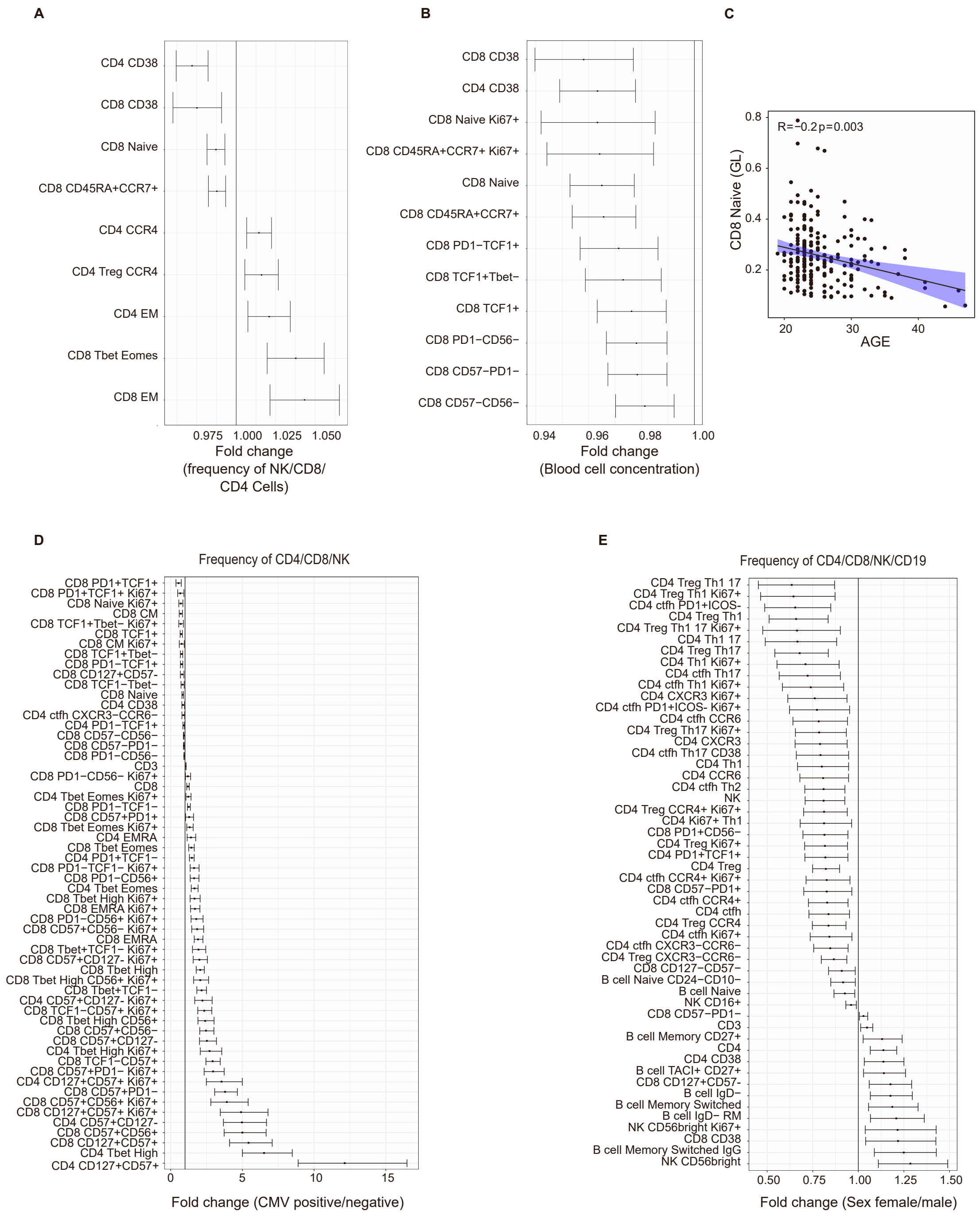


**Figure S3. Effect of Age, CMV and Sex on baseline immune parameters.** Related to Figures 1 and 2 and Table S2.

A-B) Effect of age on the frequency of circulating immune cells (A) or their concentration in blood (B) based on multiple regression analysis for CD8+, CD4+, B, and NK cell subsets. Fold changes corresponding to each additional year of age are displayed only for comparisons that meet the false discovery rate (FDR) threshold (< 0.1). Horizontal bars represent 95% confidence intervals (2.5-97.5%).
C) Spearman correlation between age and the concentration of naïve CD8+ T cells in blood (n = 238).
D-E) Effect of CMV infection (D) and sex (E) on the frequency of circulating immune cells, estimated using a multiple regression model for CD8+, CD4+, B, and NK cell parameters. Fold changes are reported only for significant comparisons (FDR < 0.1), with horizontal bars indicating the 2.5-97.5% confidence intervals. Sample sizes and linear model results can be seen in Tables S1 and S3. Phenotypic markers of T cell populations depicted in the figure are as follows: TSCM (CD45RA+CCR7+CD95+); Naïve (CD45RA+CCR7+CD95-); CM (CD45RA-CCR7+); EM (CD45RA-CCR7-); EM1 (CD45RA-CCR7-CD27+); EM2 (CD45RA-CCR7-CD27-); EMRA (CD45RA+CCR7-); pE (CD45RA+CCR7-CD27+); E (CD45RA+CCR7-CD27-); Effector (CD45RA+CD27-); cTfh (CXCR5+); Treg (FoxP3+CD25+CD127-); Th1 (CCR6-CXCR3+); Th17 (CCR6+CXCR3-); Th1-17 (CCR6+CXCR3+); Th2 (CCR6-CXCR3-CCR4+).
Phenotypic markers of B cell populations depicted in the figure are as follows: AM (CD27+CD21-); RM (CD27+CD21+); TLM (CD27-CD21-); IM (CD27-CD21+); plasmablasts (CD20-CD38+); Naïve (IgD+CD27-); Memory (CD27+); DN (IgD-CD27-); DN1 (DN, CXCR5+CD21+); DN2 (DN, CXCR5-CD21-); Memory switched (CD27+IgD-IgM-); Memory pre-switched (CD27+IgD-IgM+).

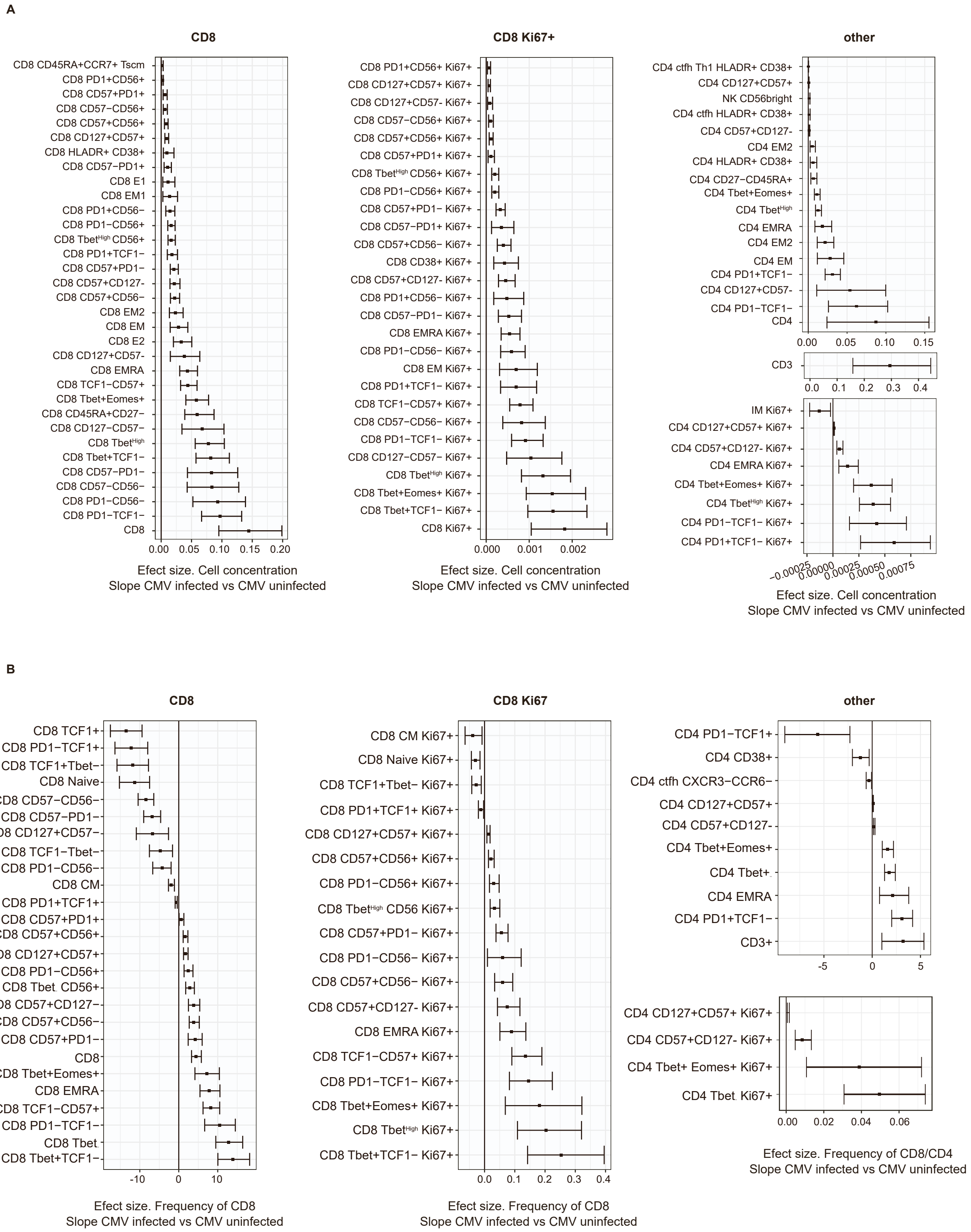


**Figure S4. Effect size of CMV infection on blood cell concentrations and population frequencies.** Related to Figure 1 and Table S2.

A) The estimated slope representing the difference in cell concentrations between CMV-infected and uninfected individuals calculated using a multiple regression model. B) The slope, representing the difference in CD8/CD4/NK frequencies between CMV-infected and uninfected individuals estimated by multiple regression. CD8, CD4 and CD3 values represent the difference in lymphocyte frequency.
Only comparisons passing a multiple testing correction (FDR<0.1) are shown. Horizontal bars indicate the 2.5-97.5 % confidence intervals. For sample sizes and further linear model results, see table S3.
Phenotypic markers of T cell populations depicted in the figure are as follows:: TSCM (CD45RA+CCR7+CD95+); CM (CD45RA-CCR7+); EM (CD45RA-CCR7-); EM1 (CD45RA-CCR7-CD27+); EM2 (CD45RA-CCR7-CD27-); EMRA (CD45RA+CCR7-); pE (CD45RA+CCR7-CD27+); E (CD45RA+CCR7-CD27-); Effector (CD45RA+CD27-); cTfh (CXCR5+).
Phenotypic markers of B cell populations depicted in the figure are as follows: AM (CD27+CD21-).

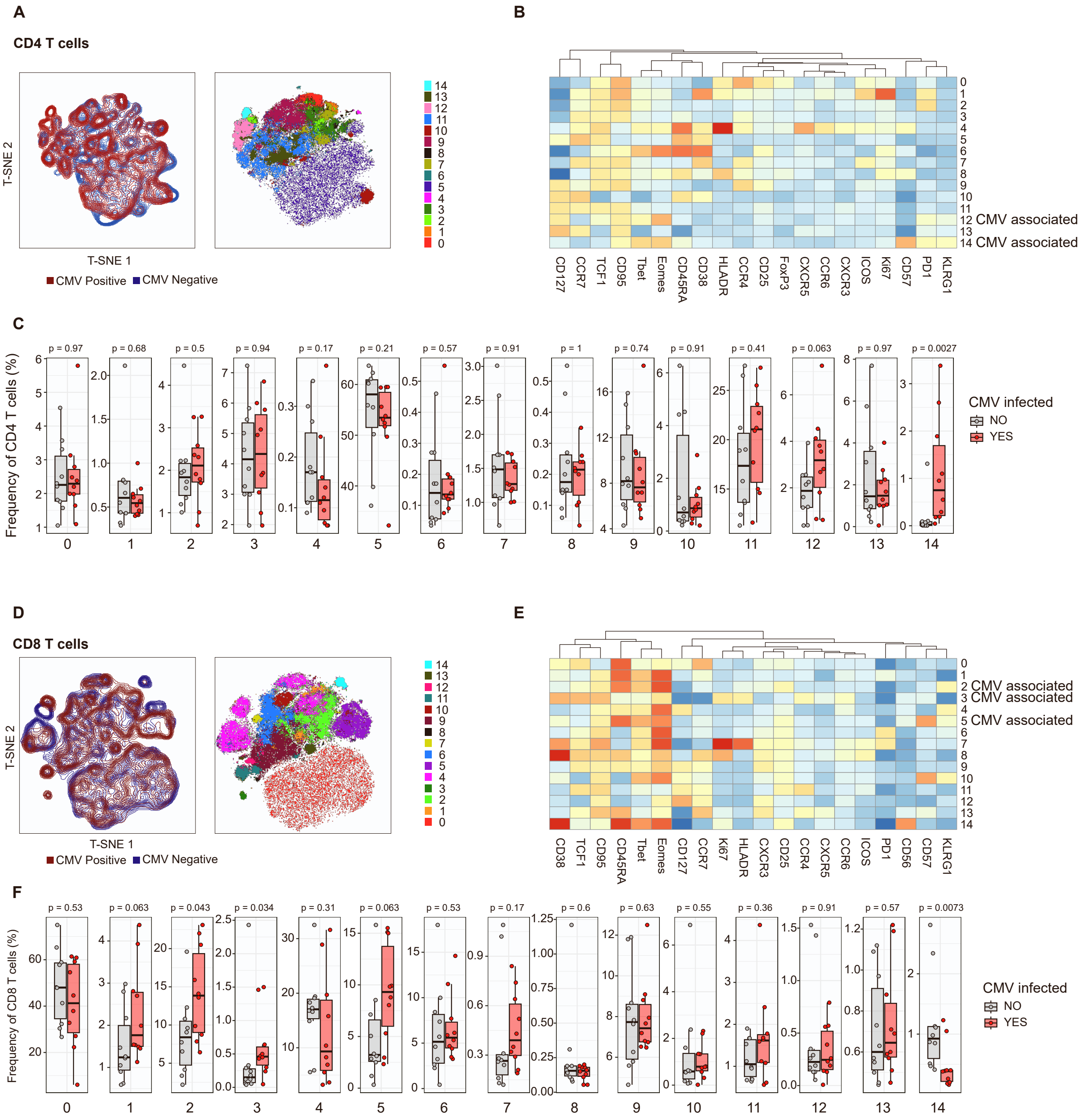


**Figure S5. Dimension reduction and FlowSOM clustering of baseline CD4 and CD8 T cell populations.** Related to Figure 1.

A) T-SNE dimensional reduction of CD4 T cells. The left panel visualizes the overlay of CMV-infected and uninfected groups on the T-SNE plot, while the right panel shows 14 clusters generated by FlowSOM clustering. B) Heatmap displaying the expression levels of 20 CD4 T cell markers across the 14 FlowSOM-identified clusters, with distinct expression patterns for each cluster. C) Boxplots illustrating the frequency of each CD4 T cell cluster for CMV-infected and uninfected individuals. D) T-SNE dimensional reduction of CD8 T cells. The left panel overlays CMV-infected and uninfected groups, and the right panel shows the 14 clusters identified by FlowSOM clustering. (E) Heatmap displaying the expression levels of 20 CD8 T cell markers across the 14 clusters, highlighting differential marker expression. (F) Boxplots displaying the frequency of each CD8 T cell cluster for CMV-infected and uninfected individuals.

The analysis was conducted on a cohort of 20 individuals (10 CMV-positive and 10 CMV-negative). For each individual, the analysis was restricted to 20,000 CD4 T cells and 12,900 CD8 T cells. Boxplots show a horizontal line indicating the median and lower and upper hinges corresponding to the first and third quartiles. The lower and upper whiskers extend to 1.5x IQR from the respective hinge. Significance in panels C and F was assessed using the Wilcoxon rank-sum test.

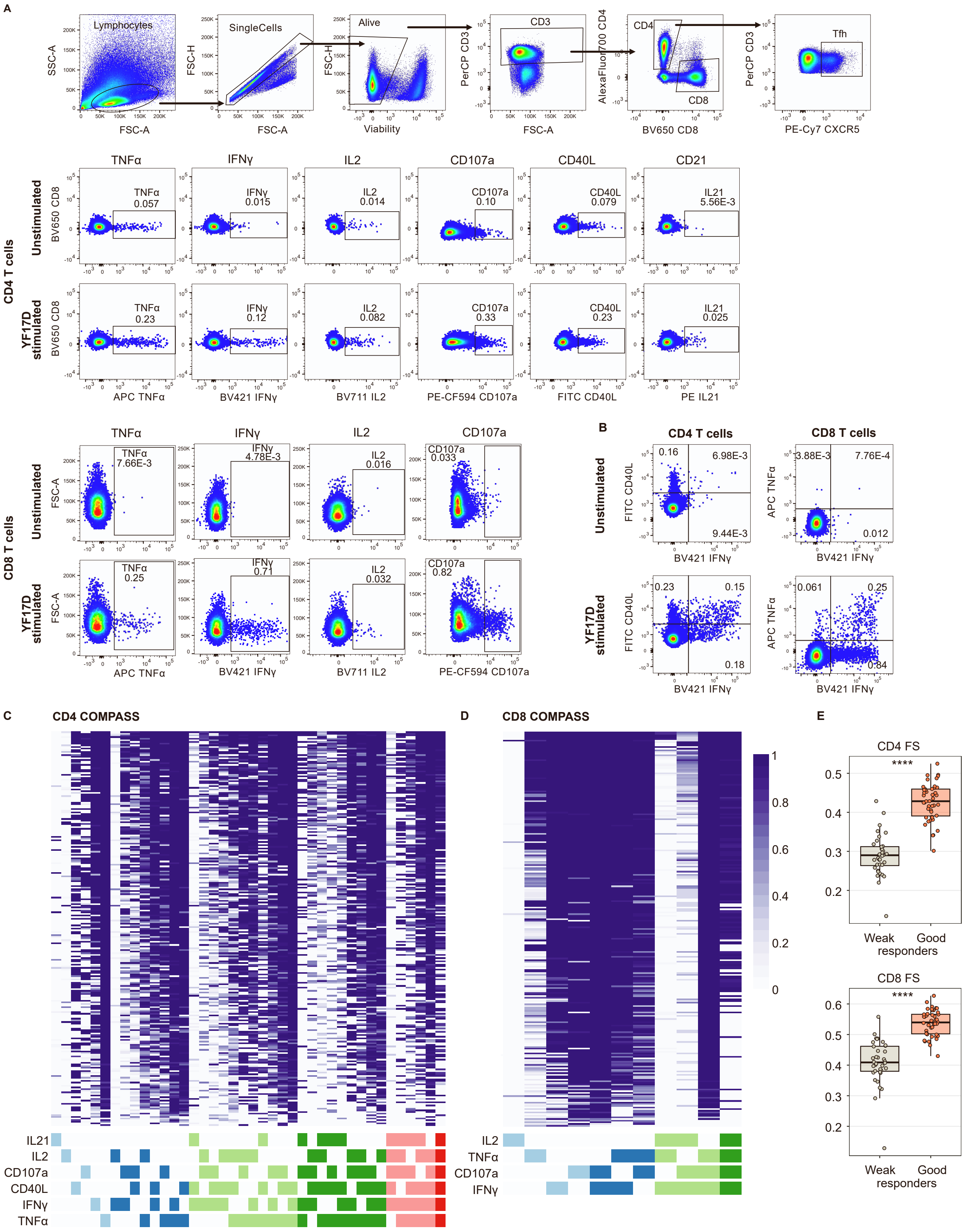


**Figure S6. YF17D ex vivo restimulation and Combinatorial Polyfunctionality Analysis of Single Cells (COMPASS) of the antigen-specific CD4 and CD8 T cell response on day 28 pv.** Related to Figure 3 and to STAR Methods, intracellular staining of ex vivo re-stimulated samples.

A) Gating strategy for intracellular cytokine staining for unstimulated and YF17D re-stimulated samples. B) Representative examples for the quantification of antigen-specific CD8 and CD4 T cells. C) Heatmap of the posterior probability of antigen-specificity for 64 functional CD4 subpopulations (n = 187 individuals) D) Heatmap of the posterior probability of antigen-specificity for 16 functional CD8 subpopulations (n = 187 individuals) E) Functionality score of the CD4 and CD8 T cell response for good (n = 44) and weak (n = 36) vaccine responders as defined in figure 4 A/B.

Functional subpopulations that lacked antigen-specificity were excluded from visualization in A and B. Boxplots show a horizontal line indicating the median and lower and upper hinges corresponding to the first and third quartiles. The lower and upper whiskers extend to 1.5x IQR from the respective hinge. Statistical significance for E was calculated with a Wilcoxon–Mann–Whitney test and depicted as **** $p<0.0001$.

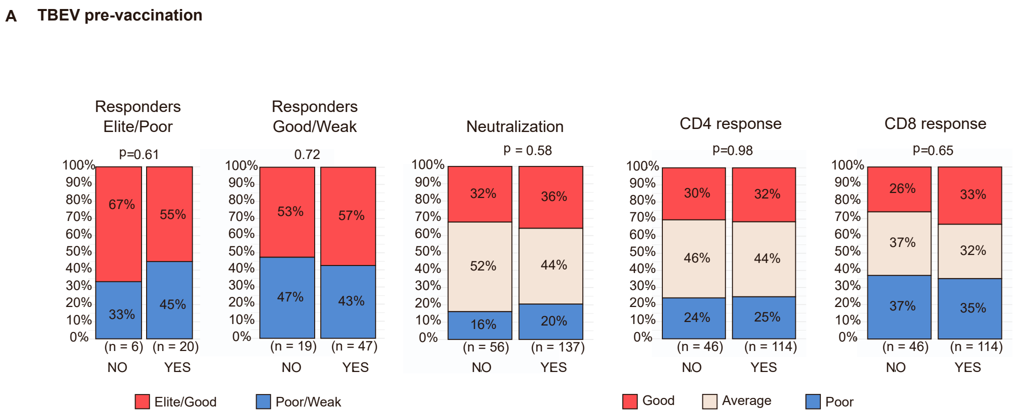


**Figure S7. Categorical classification across vaccine response groups of individuals with or without a previous TBEV vaccination.** Related to Figure 4.

TBEV-pre-vaccinated and TBEV-naive individuals were allocated to the vaccine response groups defined in Figure 4 A. Statistical significance was evaluated with a Chi-square test. Sample size is indicated below each comparison.

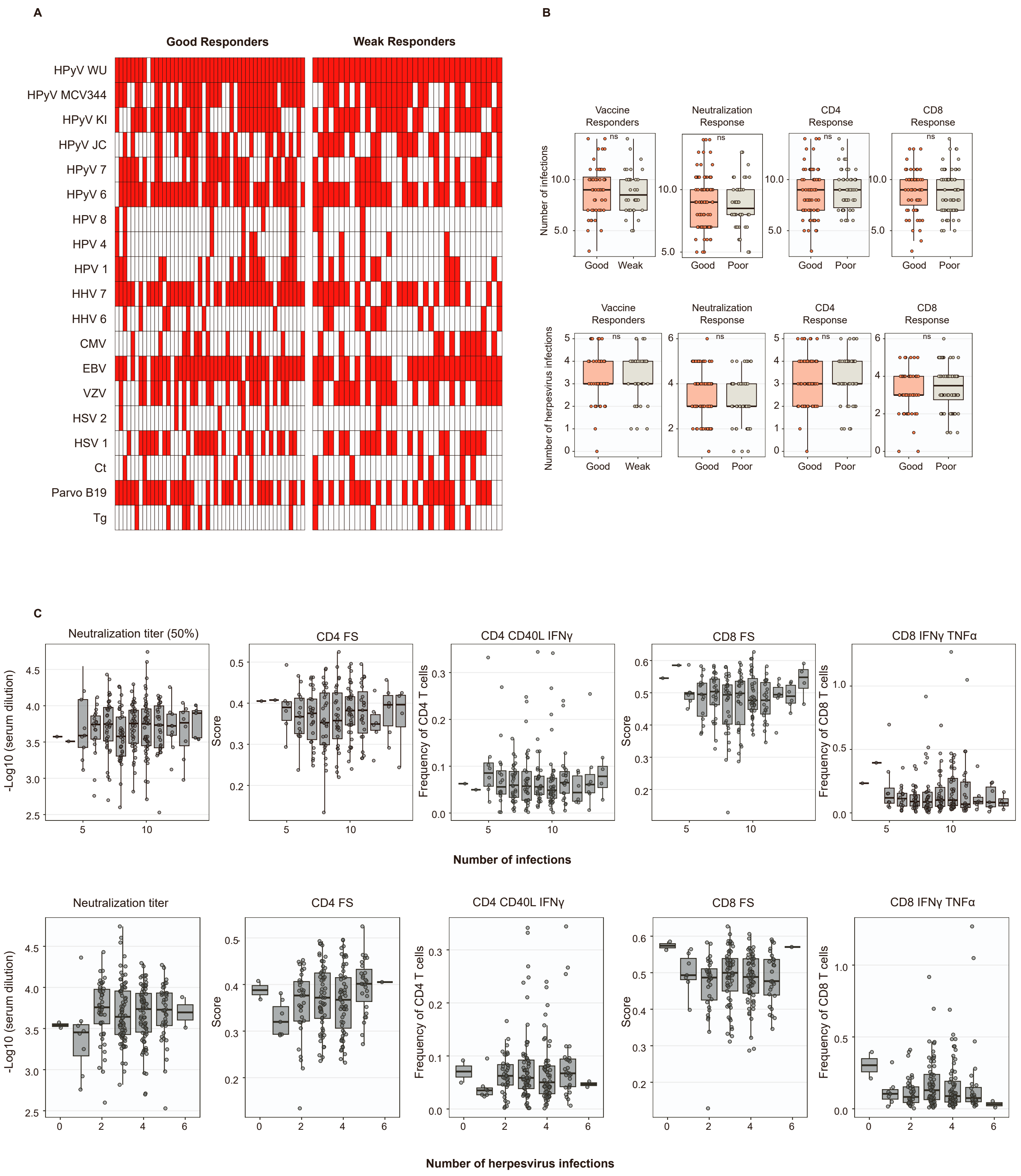


**Figure S8. No cumulative effect of prior infections on the YF17D vaccine response.** Related to Figures 3 and 4.

A) Depiction of the number of past infections for good and weak responders. Each column corresponds to an individual participant, with red indicating a positive infection history and white indicating a negative infection history for each assessed pathogen. B) Boxplots illustrating the distribution of the total number of infections (top panels) and herpesvirus infections (bottom panels) across good (n = 49) and weak (n = 37) responders, as well as good and poor neutralization groups (n = 90 and n = 48, respectively), and elite/good and poor CD4 (n =67 and n = 51, respectively) and CD8 responses (n = 60 and n = 73, respectively) as categorized in Figure 4A. C) Boxplots displaying the magnitude of selected vaccination endpoints for the study participants stratified by the number of prior infections. Boxplots show a horizontal line indicating the median and lower and upper hinges corresponding to the first and third quartiles. The lower and upper whiskers extend to 1.5x IQR from the respective hinge. Statistical significance was calculated with a Wilcoxon–Mann–Whitney test for B and C with the designation: ns (non-significant), *p ≤ 0.05, **p ≤ 0.01, ***p ≤ 0.001, ****p ≤ 0.0001.

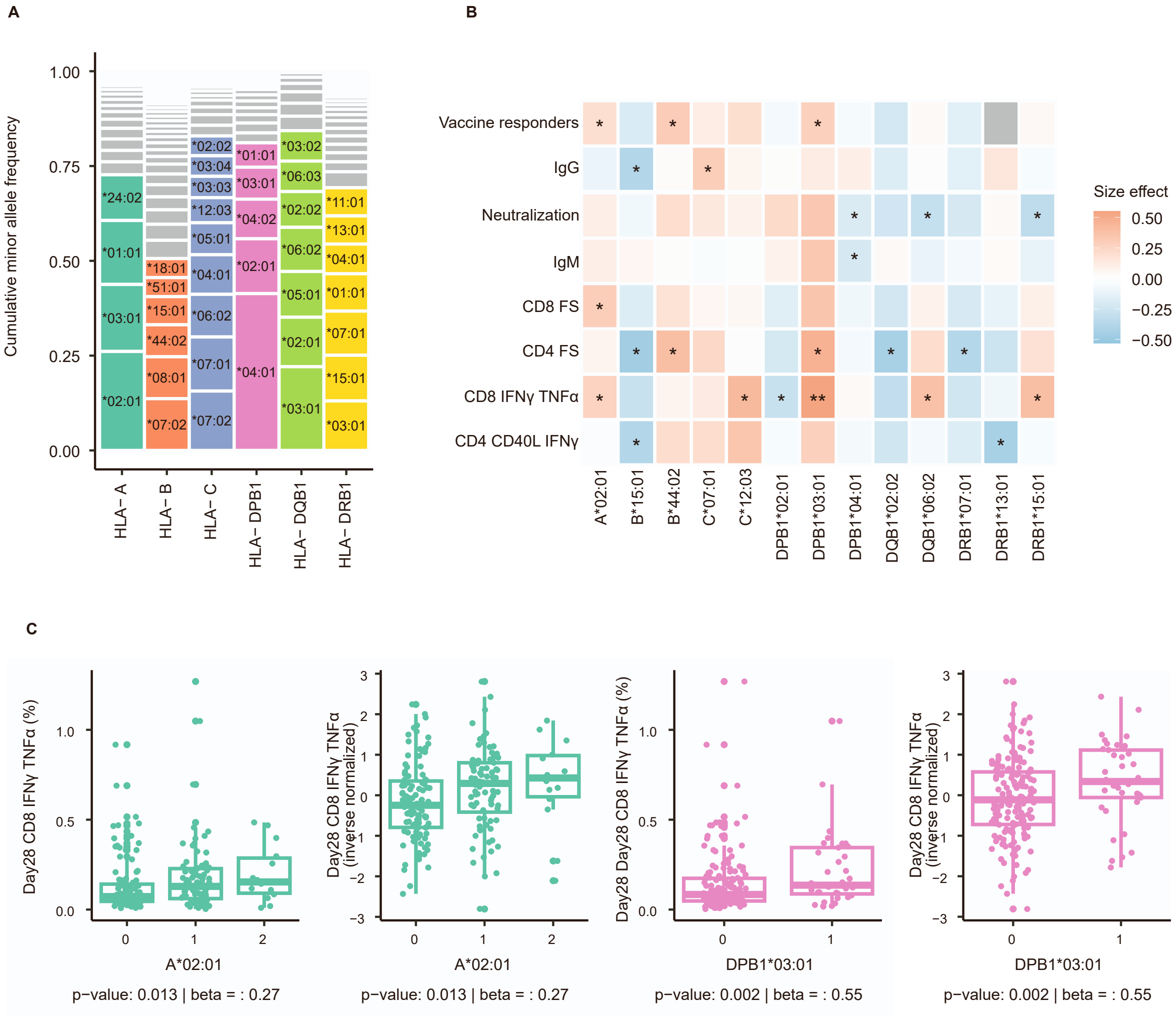


**Figure S9. Association analysis of HLA alleles and vaccination outcomes.** Related to Figures 4 and 5.

A) Frequency of HLA alleles for the 6 assessed HLA genes. Alleles with minor allele frequency < 0.05 were excluded from the analysis and are shown in grey. B) Association analysis results are shown for alleles with statistical evidence of association (p-value < 0.05) for at least one of the vaccination outcomes. The color gradient reflects the effect size and direction of the association, with statistical significance indicated by symbols (* = p ≤ 0.05, ** = p ≤ 0.01). Grey fields indicate combinations of alleles and outcomes that were not tested due to minor allele frequency threshold. C) Individual-level data for HLA-A*02:01 and for HLA-DPB1*03:01 grouped based on the number of gene copies (0, 1 or 2). For each allele, both the raw outcome counts, and the transformed outcome counts are shown. Boxplots show a horizontal line indicating the median and lower and upper hinges corresponding to the first and third quartiles. The lower and upper whiskers extend to 1.5x IQR from the respective hinge. Statistical significance in C was estimated with a Wilcoxon rank sum test.

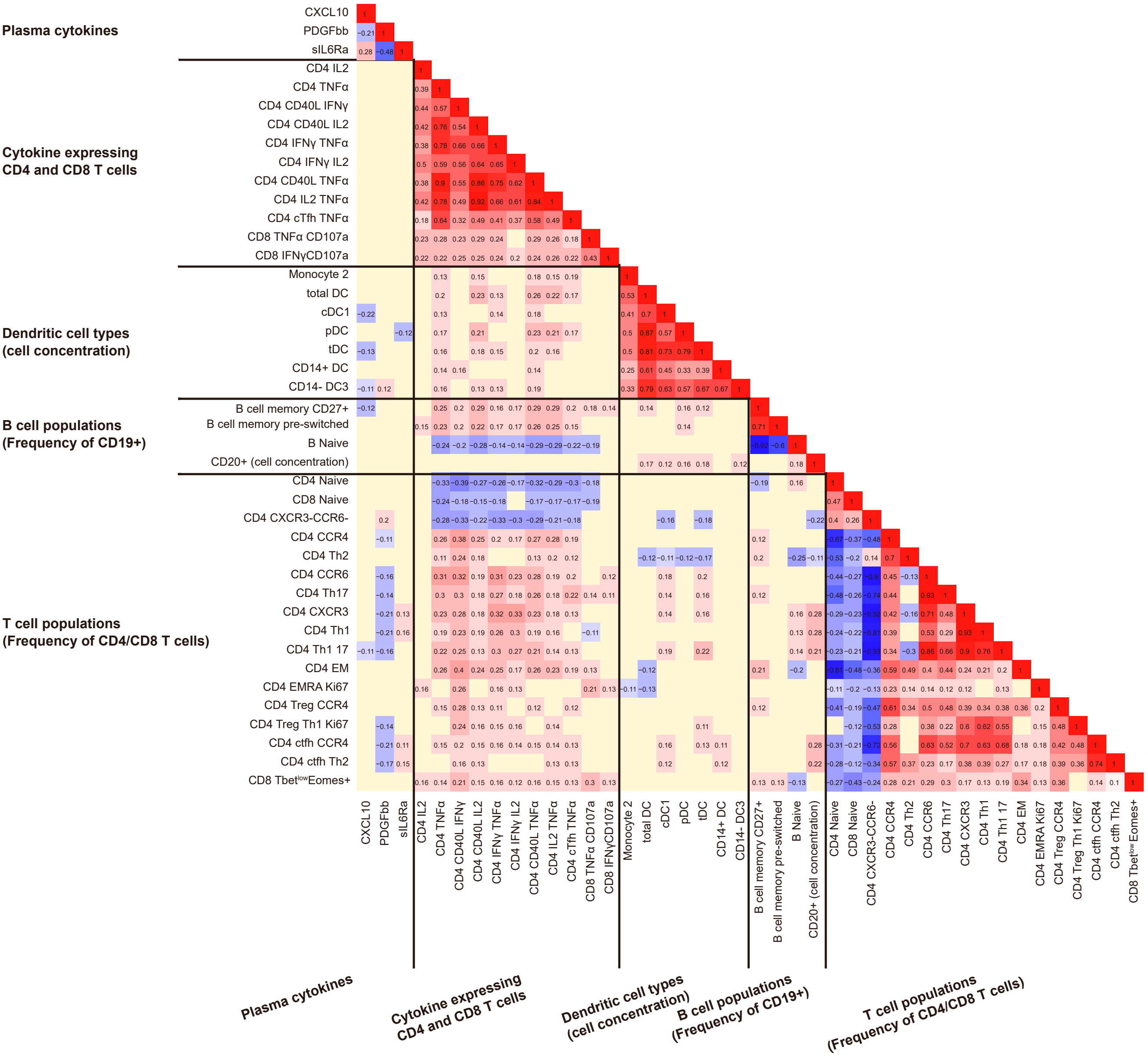


**Figure S10. Spearman correlation of baseline parameters.** Related to Figures 6 and 7.

This heatmap displays the Spearman correlation coefficients for statistically significant associations between baseline parameters (p-value < 0.05). The color gradient represents the magnitude and direction of the correlation, with positive correlations shown in the red spectrum and negative correlations in blue. Only significant correlations are shown, and the intensity of the color reflects the correlation spearman coefficient.

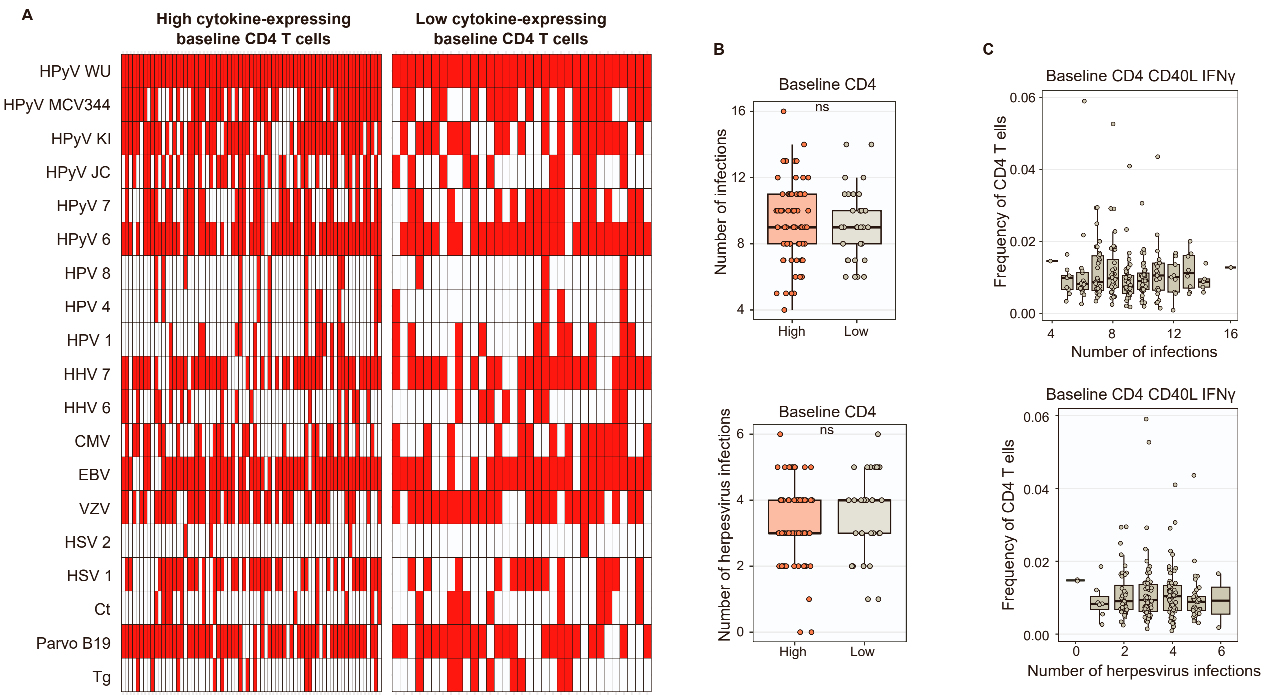


**Figure S11. The cumulative number of prior infections is not associated with baseline cytokine-expressing CD4 T cells.** Related to Figures 6 and 7.

A) Depiction of the number of past infections for individuals grouped by high or low levels of cytokine-expressing CD4 T cells as defined in Figure 6A. Each column represents an individual, with red indicating a positive infection history and white indicating a negative infection history for each pathogen assessed. B) Boxplots illustrating the distribution of the total number of infections (top panels) and herpesvirus infections (bottom panels) across the different baseline CD4 T cell clusters defined in Figure 6A. C) Boxplots illustrating the frequency of CD40L+ IFNγ+ CD4 T cells for the study participants stratified by the number of infections. Boxplots show a horizontal line indicating the median and lower and upper hinges corresponding to the first and third quartiles. The lower and upper whiskers extend to 1.5x IQR from the respective hinge. Statistical significance was calculated with a Wilcoxon–Mann–Whitney test for B and C with the designation: ns (non-significant), $*p \leq 0.05$, $**p \leq 0.01$, $***p \leq 0.001$, $****p \leq 0.0001$.

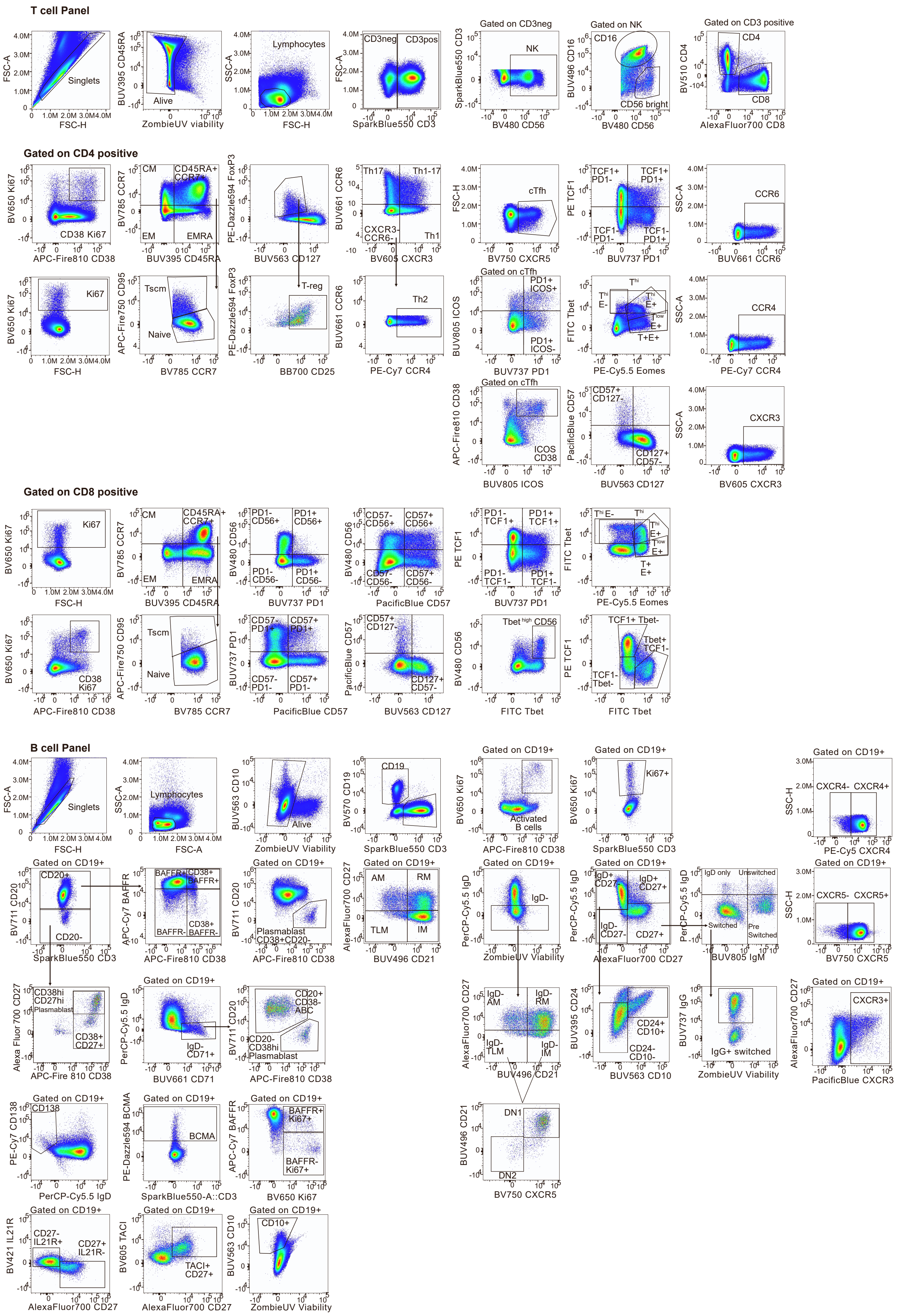


**Figure S12. Gating strategy for T cell (top) and B cell (bottom) parameters.** Related to Star Methods, Flow cytometry and phenotyping
