# Supplementary material for: Basal T cell activation predicts yellow fever vaccine response independently of cytomegalovirus infection and sex-related immune variations
Source: Cell Rep Med. 2025 Feb 11;6(2):101946. doi: 10.1016/j.xcrm.2025.101946 (PMC11866508; doi:10.1016/j.xcrm.2025.101946)
Supplement: Document S2. Article plus supplemental information [file mmc4.pdf]

Cell Reports
Medicine

Article

Basal T cell activation predicts yellow fever vaccine response independently of cytomegalovirus infection and sex-related immune variations

Graphical abstract

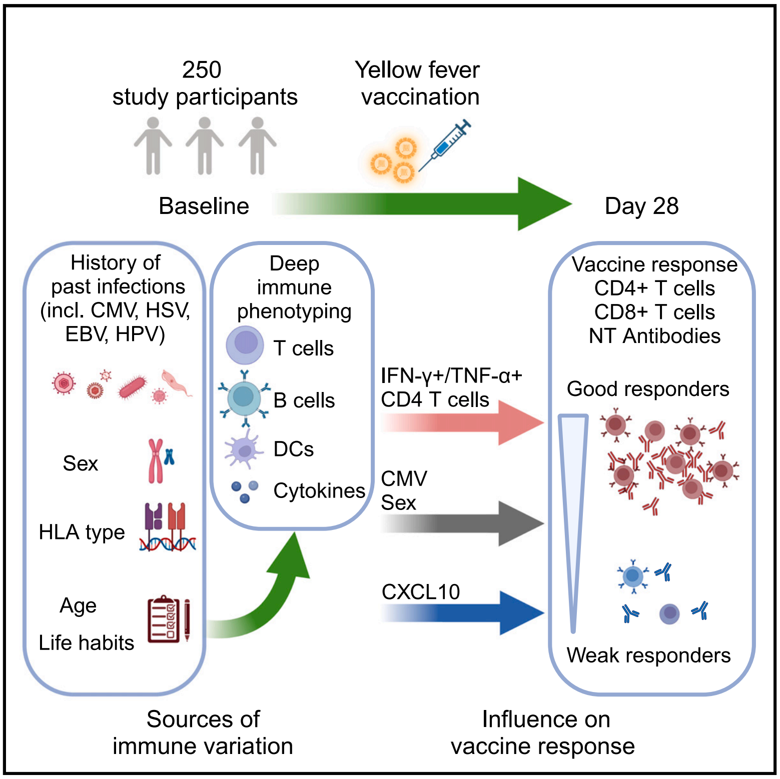

Authors

Antonio Santos-Peral,
Magdalena Zaucha, Elena Nikolova, ...,
Tim Waterboer, Anne B. Krug,
Simon Rothenfusser

Correspondence

simon.rothenfusser@med.uni-muenchen.de

In brief

The immune response to live vaccines varies significantly among individuals, but the factors influencing vaccine performance remain unclear. Santos-Peral et al. find that sex and cytomegalovirus infection do not impact the yellow fever vaccine outcomes. Instead, environmental factors shape a baseline T cell activation state linked to enhanced vaccine responses.

Highlights

- Sex and CMV status affect baseline immune variation but not YF17D vaccine outcomes
- High numbers of activated $CD4^+$ T cells at baseline link to stronger vaccine responses
- Elevated baseline CXCL10 levels are negatively associated with YF17D vaccine responses
- Environmental factors contribute to the baseline immune status tied to vaccine response

Santos-Peral et al., 2025, Cell Reports Medicine 6, 101946
February 18, 2025 © 2025 The Author(s). Published by Elsevier Inc.
https://doi.org/10.1016/j.xcrm.2025.101946

CellPress

Cell Reports Medicine

CellPress
OPEN ACCESS

Article

Basal T cell activation predicts yellow fever vaccine response independently of cytomegalovirus infection and sex-related immune variations

Antonio Santos-Peral,[1] Magdalena Zaucha,[1] Elena Nikolova,[1] Ekin Yaman,[2,3] Barbara Puzek,[2,3] Elena Winheim,[4] Sebastian Goresch,[1] Magdalena K. Scheck,[1] Lisa Lehmann,[1] Frank Dahlstroem,[1] Hadi Karimzadeh,[1] Julia Thorn-Seshold,[1,5] Shenzhi Jia,[1] Fabian Luppa,[6] Michael Pritsch,[6] Julia Butt,[7] Camila Metz-Zumaran,[7] Giovanna Barba-Spaeth,[8] Stefan Endres,[1,9] Sarah Kim-Hellmuth,[2,3] Tim Waterboer,[7] Anne B. Krug,[4] and Simon Rothenfusser[1,9,10,*]

[1]Division of Clinical Pharmacology, LMU University Hospital, LMU Munich, Munich, Germany
[2]Department of Pediatrics, Dr. von Hauner Children's Hospital, LMU University Hospital Munich, Munich, Germany
[3]Institute of Translational Genomics, Department of Computational Health, Helmholtz Munich, Munich, Germany
[4]Institute for Immunology, Biomedical Center, Faculty of Medicine, LMU Munich, Munich, Germany
[5]Faculty of Chemistry and Pharmacy, LMU Munich, Munich, Germany
[6]Division of Infectious Diseases and Tropical Medicine, LMU University Hospital, LMU Munich, Munich, Germany
[7]Division of Infections and Cancer Epidemiology at the German Cancer Research Center (DKFZ), Heidelberg, Germany
[8]Institut Pasteur, Université de Paris, CNRS UMR 3569, Unité de Virologie Structurale, Paris, France
[9]Einheit für Klinische Pharmakologie (EKLiP) Helmholtz Zentrum München German Research Center for Environmental Health (HMGU), Neuherberg, Germany
[10]Lead contact
*Correspondence: simon.rothenfusser@med.uni-muenchen.de
https://doi.org/10.1016/j.xcrm.2025.101946

SUMMARY

The live-attenuated yellow fever 17D (YF17D) vaccine is a model of acute viral infection that induces long-lasting protective immunity. Among immunocompetent adults, responses to YF17D vary significantly. To understand the sources of this variability, we investigate the influence of sex, age, human leukocyte antigen (HLA) type, and 20 prior infections on basal immune parameters and the cellular and antibody response to YF17D in 250 healthy young individuals. Multivariate regression found that sex and cytomegalovirus (CMV) infection significantly contribute to baseline immune variation but do not affect vaccine responses except for reduced YF17D-specific $CD8^+$ frequencies in CMV-infected males. However, the abundance at baseline of non-specific cytokine-expressing T helper cells in circulation is associated with stronger vaccine responses, a state that smoking favors. Additionally, an elevated baseline level of interferon-stimulated CXCL10 is linked to poorer vaccination outcomes. Altogether, YF17D reactivity is conditioned by the baseline immune status independent of sex and CMV-related variations.

INTRODUCTION

Vaccine responses vary considerably among healthy individuals due to genetic, intrinsic, and environmental factors.[1] Identifying mediators of immune variation that affect the quality of immune responses is crucial to understanding vaccine effectiveness and a given subject's predisposition and susceptibility to pathogens.

The natural variation in immune parameters is explained partly by genetic determinants.[2,3] For instance, human leukocyte antigen (HLA) alleles have been associated with vaccine efficacy and immune responses.[4,5] Intrinsic factors such as sex and age have an unequivocal role in molding host immune parameters and the course of response to infectious agents and vaccinations.[1,6] Aging gradually leads to a functional decline of the immune system that corresponds to the cumulative history of infections and exposure to environmental stressors.[7–9] Likewise, sex is also a major immune determinant. Females exhibit higher lymphocyte counts and immunoglobulin levels and generally mount stronger innate and adaptive immune responses.[1,2,10–14]

Baseline host immune variability can also be partially explained by the constant interaction with chronic or latent infections as well as the accumulation of pathogen exposures. Widespread pathogens like cytomegalovirus (CMV), Epstein-Barr virus (EBV), or herpes simplex viruses (HSV) 1 and 2 have a lifelong persistence combining latency with intermittent reactivations. Virus-mediated immunomodulation and the constant immune reaction against them result in profound alterations of the host immune system.[10] Specifically, CMV-infected individuals show an accumulation of differentiated effector $CD4^+$ and $CD8^+$ T cells, and a substantial fraction of their memory T cell

This is an open access article under the CC BY license (http://creativecommons.org/licenses/by/4.0/).

pool is CMV specific.[2,11–13] Nevertheless, the effect of persistent infections on immune function remains controversial, and there is no unequivocal pattern exerted by CMV on vaccine responses.[14] Reports studying the effect of CMV infection on influenza vaccine responses run the gamut from it being favorable for young adults[15] and the elderly[16] to reduced responses for both age groups.[17–19] Others have reported no impact[20] or only reduced CD8$^+$ T cell responses in older adults.[21]

Research efforts have been directed toward identifying baseline markers capable of predicting vaccine responses. Female sex, lower height, and higher total white blood cells were recognized as predictors of high responder status for 26 HIV vaccine studies.[22] The strength of influenza vaccine responses could be predicted by the frequency of circulating immune cell subsets including effector memory (EM) CD4$^+$ T cells, activated CD4$^+$ and CD8$^+$ T cells, dendritic cells (DCs), as well as CD20$^+$CD38$^+$ B cells.[23–26] Likewise, systems vaccinology approaches have proposed gene expression signatures predictive of vaccine responses.[26–29] However, these predictors might not be common to all vaccine types, especially live vaccines.[28] The response to live vaccines depends on a complex balance between viral replication, attenuation, and the immunogenicity of the vaccine virus. Therefore, the immunological factors that characterize the response to inactivated vaccines differ from those of live vaccines.[29,30]

Here, we aimed to elucidate the interplay between intrinsic (sex, age, and HLA type), non-genetic, and environmental factors in shaping the pre-vaccination baseline immune state and its impact on the response to the live-attenuated yellow fever 17D (YF17D) vaccine. Despite the exceptional performance of the YF17D vaccine, which elicits a long-lasting protective immunity mediated by neutralizing antibodies and T cell responses,[31–34] basal immune setpoints that impact the YF17D response and their causal factors remain largely understudied. In this study, we integrated data on 20 previous infections, sex, age, HLA type, lifestyle information, and a comprehensive set of baseline immune parameters with cellular and humoral YF17D vaccination endpoints in a cohort of 250 healthy young adults. We identify sex and CMV as major sources of variability in the basal immune status and clarify that these factors do not affect the immunogenicity of the YF17D vaccine. Furthermore, we identify the abundance of nonspecific cytokine-expressing differentiated CD4$^+$ T helper cells in circulation and the amount of CXCL10 as setpoints predicting the T cell and antibody response to the YF17D vaccine. Additionally, we recognize smoking as a potential extrinsic environmental factor influencing this specific phenotype.

RESULTS

Intrinsic factors and infection history impact on basal host-immune parameters

250 healthy young individuals were vaccinated with the YF17D vaccine (Stamaril). Approximately two-thirds of the study cohort (n = 139) had been vaccinated against tick-borne encephalitis virus (TBEV), a factor that we have previously identified as influencing the response to YF17D by expanding cross-reactive antibodies but with limited impact on the neutralizing antibody response.[35] Peripheral blood mononuclear cell and serum samples were analyzed at baseline (before YF17D vaccination) and day 28 post vaccination. Baseline serum was evaluated for specific IgG responses to multiple antigens from the following infective agents: persistent viruses including human papillomaviruses 1, 4, and 8; polyomaviruses JCPyV, KIPyV, WUPyV, HPyV 6, HPyV 7, and MCV; human herpesviruses like herpes simplex 1 (HHV1/HSV1), herpes simplex 2 (HHV2/HSV2), varicella zoster (HHV3/VZV), EBV (HHV4/EBV), human CMV (HHV5/CMV), HHV6 A and B, and HHV7; a persistent parasite, *Toxoplasma gondii*; bacteria, *Chlamydia trachomatis* and *Mycoplasma genitalium*; and an acute past viral infection, parvovirus B19. Antigens used for serodiagnosis are summarized in Figure S1. Serology results indicated that large fractions of the cohort were infected with EBV (85%), HHV7 (75%), WUPyV (97%), and HPyV 6 (81%). About half of the participants were positive for HSV1 (50%) and VZV (58%), and 33% of the study participants were chronically infected with CMV (Figure S1).

To define the basal immune status of the study participants, we unified a dataset of 786 immune parameters. These include the concentration of 18 cytokines in plasma, C-reactive protein (CRP), immunoglobulin concentrations, lymphocyte concentration and frequency in the peripheral blood of CD4$^+$ and CD8$^+$ T cell subsets, DC subsets, monocytes, and B cells. In addition, the activation state of DCs, monocytes, T and B cells, as well as basal T cell cytokine expression was incorporated in the dataset (Table S1A).

First, we performed a principal component (PC) analysis on the 768 baseline parameters to identify the main sources of variation. We observed that the dominant contributing factors for each respective PC were groups of related immunological parameters (Figures S2A and S2B; Table S1B). Next, we examined whether the variation in baseline parameters, summarized by the PCs, was associated with independent intrinsic and non-genetic factors. While these variables could not explain PC1, PC2, and PC3, we identified a significant difference of means in PC4 values (characterized by the abundance of lymphocytes, CD8$^+$ and CD4$^+$ T cells) between CMV-infected and uninfected participants (Figure S2C). Next, to address the independent impact of sex, age, and the history of infections on the basal immune status, we implemented a multivariate linear regression model in which 22 independent variables (sex, age, and prior infection with 20 pathogens) were evaluated against the 768 basal immune parameters (see STAR Methods and results in Table S2). Corroborating the principal component analysis results, the model identified CMV infection, but not other previous infections, as well as sex, as having the strongest effects, explaining a significant part of the variability in baseline immune parameters (Figure 1A; and in more detail further).

The effect of age we observed was limited by the homogeneously young age of the study participants. Nevertheless, with increasing age, participants tended to have lower naive CD8$^+$ T cell counts and decreased TCF1$^+$ T cells (Figure S3), an increase in the frequency of EM T cells and CD8$^+$ T cells that were positive for both Tbet and Eomes, and a significantly lower frequency of CD38$^+$ cells within CD4$^+$ and CD8$^+$ T cells, which might suggest a gradual decline in function and activity (Figures S3A–S3C).

CellPress
OPEN ACCESS

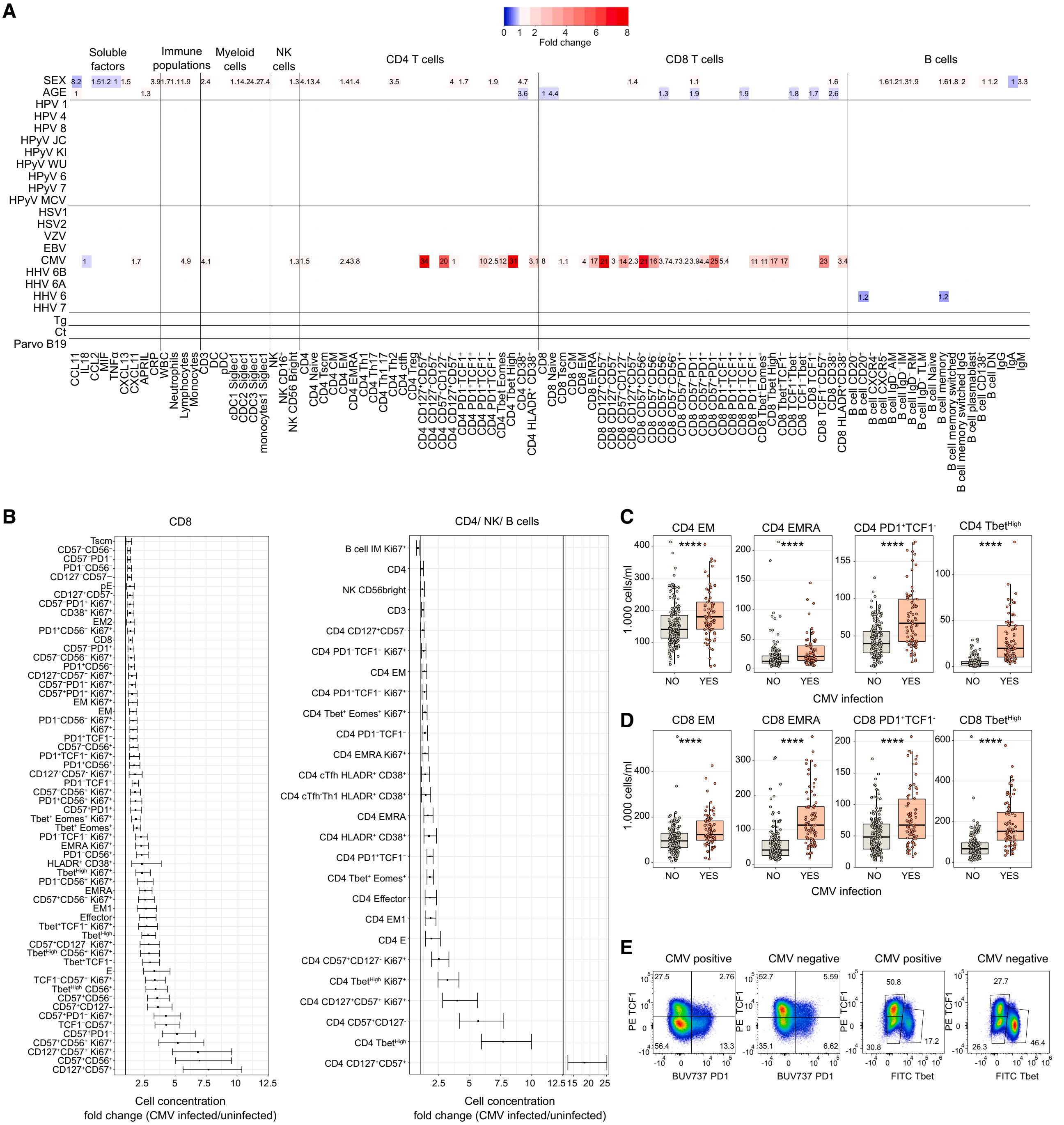

Figure 1. Effect on baseline immune parameter variation of intrinsic factors and prior infections

(A) Multivariate linear regression analysis of baseline immune parameters and 20 prior infections, sex, and age. Only a selection with positive hits is included. Numbers indicate the −log10 of the q-value for the effect of the independent variable. Color scale represents the fold change between positive/negative infection or female/male sex or with a yearly increase of age. Only comparisons passing a multiple testing correction (false discovery rate < 0.1) are depicted for fold change.

(B) CMV infection effect on the number of circulating immune cells for CD8$^+$, CD4$^+$, B, and NK cell parameters. Fold change between infected/uninfected individuals is shown only for comparisons passing a multiple testing correction. Horizontal bars indicate the 2.5%–97.5% confidence intervals.

(C and D) Direct comparison of the concentration in blood of different cell populations between CMV-infected and uninfected individuals for CD4$^+$ (C) and CD8$^+$ (D) subpopulations (n = 158 CMV-negative and n = 78 CMV-positive donors).

(legend continued on next page)

CMV infection leads to an accumulation of memory T cell subsets

In line with previous studies, individuals infected with CMV exhibited a 1.4-fold increase in the CD8$^+$ T cell population and a 1.2-fold increase in the CD4$^+$ T cell population, corresponding to increases in cell concentrations of 150,000 and 90,000 cells/mL, respectively (Figures 1B and S4A). This increase resulted from the accumulation of late-stage differentiated cells that express markers associated with senescence and exhaustion, such as CD57$^+$ and PD1$^+$, and are negative for TCF1 (Figures 1B–1E). Confirming this observation, FlowSOM clustering identified higher frequencies of CD4$^+$ T cells expressing KLRG1, PD1, CD57, CD95, Tbet, and Eomes, negative for TCF1 and CD127 in CMV-infected individuals (Figures S5A–S5C) likely characterizing late-effector and exhausted cells without self-renewal capacity. Similarly, CMV-infected donors expanded CD8$^+$ T cells expressing CD45RA, CD95, Tbet, and Eomes, negative for CD127 and TCF1. CMV-infected individuals also exhibited increased frequencies and concentrations of Ki67$^+$, HLA-DR$^+$, and CD38$^+$-activated proliferating cells (Figures 1B, S3, S4, and S5D–S5F) and elevated Tbet-high CD4$^+$ and CD8$^+$ T cells (Figures 1C and 1D). The expansion and accumulation of CCR7$^-$CD45RA$^-$ EM (1.6-fold and 28,000 cells/mL increase) and CCR7-CD45RA$^+$ (EMRA, 2.6-fold and 43,500 cells/mL increase) subpopulations were more apparent for CD8$^+$ T cells, but also existent for CD4$^+$ T cells (Figures 1B, 1C, and S4), which collectively contributed to an increased number of CD3$^+$ T cells in blood, estimated to be around 300.000 cells/mL higher in the CMV-infected group (Figures 1B–1E, S3D, and S4). The expansion of late-stage differentiated and effector T cells coincided with a reduction in the frequency of TCF1$^+$ Tbet$^-$ and naive cells (Figures S3D and S4B), while the total count of naive CD4$^+$ and CD8$^+$ T cells remained unaffected. CMV infection did not seem to impact the baseline abundance of B cells or the levels of immunoglobulins.

In contrast, past infections other than CMV did not add variation to the frequency or absolute numbers of immune cells in circulation. These results indicate that CMV profoundly affects the distribution of circulating lymphocyte subsets in young adults.

Sex assigned at birth conditions the basal distribution of immune cell subsets and immunoglobulin levels and has additive effects on CMV infection

Sex was a key determinant of the number and type of lymphocytes found in circulation (Figures 2A–2C; Figure S3E; Table S2). Females had on average 170.000 more CD4$^+$ T cells/mL than males, especially of the naive (CCR7$^+$CD45RA$^+$) compartment that was 1.3-fold more abundant (110.000 cells/mL more, Figure 2A; Table S2). Females exhibited elevated counts of Th2 cells and CD4$^+$CD127$^+$CD57$^-$ T cells. Additionally, there was a significant increase in CD38-expressing circulating follicular helper T cells (cTfh) and CD4$^+$ and CD8$^+$ T cells in females compared to males (Figure 2A). Furthermore, females displayed higher counts of effector and late-stage differentiated CD4$^+$ T cells and increased counts of CD56-bright natural killer cells and CXCR5$^-$ B cell together with various B cell subsets including memory B (CD27$^+$), IgG$^+$ class-switched, activated memory (AM, IgD$^-$CD21$^-$CD27$^+$), and resting memory (RM, IgD$^-$CD21$^+$CD27$^+$) B cells (Figure 2A). Males displayed higher levels of eotaxin and tumor necrosis factor alpha (TNFα) in plasma. Interestingly, a higher expression of SIGLEC-1 (CD169) in antigen-presenting cells, higher levels of CRP (Figure 2B), and IgM antibody levels (Figures 2B and 2C) further characterized the female population.

Since both CMV infection and the female sex were associated with increased concentration of T cells in the blood, we explored whether both factors had a combined effect. Indeed, females chronically infected with CMV had higher lymphocyte and CD3$^+$ T cell counts than infected males and uninfected females and males (Figure 2D). Even though CMV infection led to the accumulation of differentiated subpopulations while sex mostly affected the naive compartment, we observed augmented amounts of EM and EMRA CD4$^+$ T cells in females infected with CMV (Figure 2D). The observed additional effect in females is likely attributable to an augmented supply of naive CD4$^+$ T cells, which in turn result in increased amounts of differentiated cells, further accumulated in CMV-infected individuals.

Infection history and intrinsic factors do not affect the outcome of YF17D vaccination

Given the profound effects of CMV and sex on the basal immune system, we investigated their impact on the YF17D vaccine response. The following endpoints, measured on day 28 post vaccination, served as indicators of vaccine immunogenicity: the polyclonal neutralizing antibody titer, the YF17D-specific IgM and IgG antibody titers, and the functionality and the frequency of YF17D-specific CD4$^+$ and CD8$^+$ T cell responses. The functionality score (FS) was calculated using a combinatorial polyfunctionality analysis of single cells (COMPASS) analysis[36] (Figure S6).

A direct comparison between CMV-infected and uninfected groups showed a blunted CD8$^+$ response with decreased FS and lower numbers of YF17D-specific IFNγ^+TNFα^+ CD8$^+$ T cells in CMV-infected individuals, especially in the male population whereas all other vaccination endpoints seemed

(E) Representative flow cytometer identification of CD8$^+$ subpopulations. Phenotypic markers of the following T cell populations named in the figure: TSCM (CD45RA$^+$CCR7$^+$CD95$^+$), naive (CD45RA$^+$CCR7$^+$CD95$^-$), CM (CD45RA-CCR7$^+$), EM (CD45RA-CCR7$^-$), EM1 (CD45RA-CCR7-CD27$^+$), EM2 (CD45RA-CCR7-CD27$^-$), EMRA (CD45RA$^+$CCR7$^-$), pE (CD45RA$^+$CCR7-CD27$^+$), E (CD45RA$^+$CCR7-CD27$^-$), effector (CD45RA$^+$CD27$^-$), cTfh (CXCR5$^+$), Treg (FoxP3$^+$CD25$^+$CD127$^-$), Th1 (CCR6-CXCR3$^+$), Th17 (CCR6$^+$CXCR3$^-$), Th1-17 (CCR6$^+$CXCR3$^+$), and Th2 (CCR6-CXCR3-CCR4$^+$). Phenotypic markers of the following B cell populations named in the figure: AM (CD27$^+$CD21$^-$), RM (CD27$^+$CD21$^+$), TLM (CD27$^-$CD21$^-$), IM (CD27$^-$CD21$^+$), plasmablasts (CD20$^-$CD38$^+$), naive (IgD$^+$CD27$^-$), memory (CD27$^+$), DN (IgD$^-$CD27$^-$), DN1 (DN, CXCR5$^+$CD21$^+$), DN2 (DN, CXCR5$^-$CD21-), memory switched (CD27$^+$IgD-IgM$^-$), and memory pre-switched (CD27$^+$IgD-IgM$^+$). Boxplots show a horizontal line indicating the median and lower and upper hinges corresponding to the first and third quartiles. The lower and upper whiskers extend to 1.5x IQR (interquartile range) from the respective hinge.

Statistical significance in (C) and (D) was estimated with a Wilcoxon rank-sum test with the designation: ns (non-significant), *$p \leq 0.05$, **$p \leq 0.01$, ***$p \leq 0.001$, ****$p \leq 0.0001$.

See Tables S1 and S2 for linear model sample sizes and results. See also Figures S1–S5.

CellPress
OPEN ACCESS

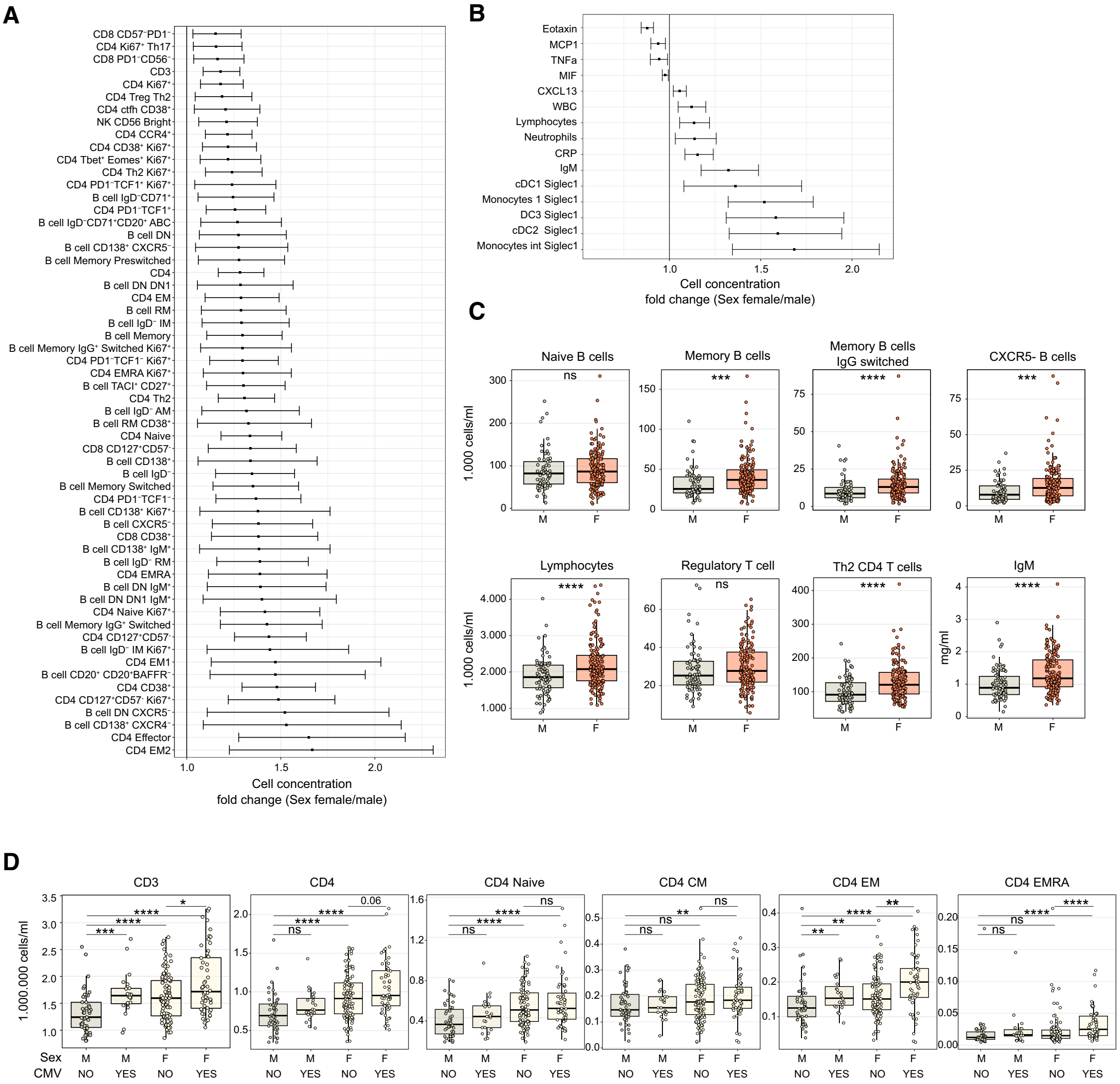

Figure 2. Sex effect on baseline immune parameters alone and in concert with CMV infection

(A and B) Cell concentrations of baseline immune populations identified in the linear model to be significantly affected by sex. Fold change difference between female and males is shown for the comparisons passing a multiple testing correction. Horizontal bars indicate the 2.5%–97.5% confidence intervals. Fold changes were calculated for all variables in cell concentration units except for cytokines (ng/mL), IgM (g/L), and siglec1 expression (mean fluorescence intensity).

(C) Comparison of immune variables between males and females (for B cell data, n = 64 males and n = 152 females; for T cell data and lymphocytes, n = 76 males and n = 162 females; for IgM data, n = 81 males and n = 169 females).

(D) Comparison of $CD3^+$ and $CD4^+$ memory subpopulations across males and females with or without CMV infection. Statistical significance against uninfected males is depicted above each group only when significant. An additional comparison is depicted between female uninfected and infected. (n = 52 CMV negative males, n = 23 CMV-positive males, n = 110 CMV-negative females, and n = 53 CMV-positive females). Phenotypic markers of the following T cell populations named in the figure: TSCM ($CD45RA^+CCR7^+CD95^+$), naive ($CD45RA^+CCR7^+CD95^-$), CM ($CD45RA-CCR7^+$), EM ($CD45RA-CCR7^-$), EM1 ($CD45RA-CCR7-CD27^+$), EM2 ($CD45RA-CCR7-CD27^-$), EMRA ($CD45RA^+CCR7$-), pE ($CD45RA^+CCR7-CD27^+$), E ($CD45RA^+CCR7-CD27^-$), effector ($CD45RA^+CD27^-$), cTfh ($CXCR5^+$), Treg ($FoxP3^+CD25^+CD127^-$), Th1 ($CCR6-CXCR3^+$), Th17 ($CCR6^+CXCR3^-$), Th1-17 ($CCR6^+CXCR3^+$), and Th2 ($CCR6-CXCR3-CCR4^+$). Phenotypic markers of the following B cell populations named in the figure: AM ($CD27^+CD21^-$), RM ($CD27^+CD21^+$), TLM ($CD27^-CD21^-$), IM ($CD27^-CD21^+$), plasmablasts ($CD20^-CD38^+$), naive (IgD^+CD27^-), memory ($CD27^+$), DN (IgD-$CD27^-$), DN1 (DN, $CXCR5^+CD21^+$), DN2 (DN, CXCR5-$CD21^-$), memory switched ($CD27^+$IgD-IgM^-), and memory pre-switched ($CD27^+$IgD-IgM^+). Boxplots show a horizontal line indicating the median and lower and upper hinges corresponding to the first and third quartiles. The lower and upper whiskers extend to 1.5x IQR from the respective hinge.

Statistical significance in (C) and (D) was estimated with a Wilcoxon rank-sum test with the designation: ns (non-significant), *$p \leq 0.05$, **$p \leq 0.01$, ***$p \leq 0.001$, ****$p \leq 0.0001$.

See Tables S1 and S2 for linear model sample sizes and results. See also Figures S1–S5.

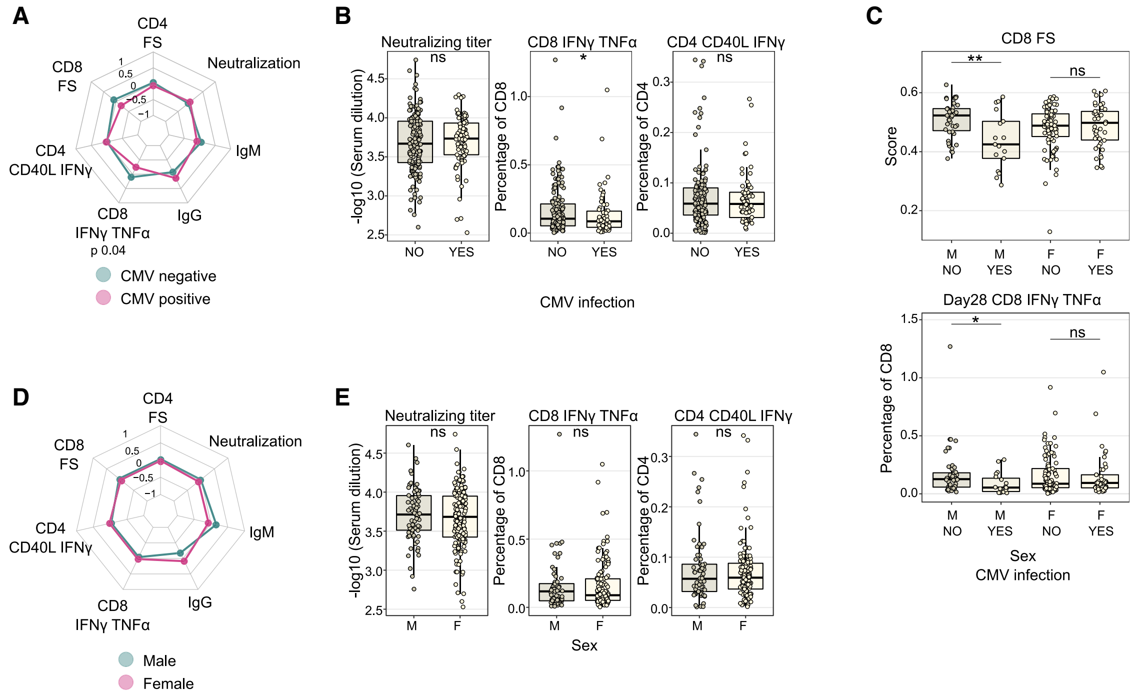

Figure 3. Sex and CMV do not influence YF17D vaccination outcome

(A and D) Radar plot associating main vaccination outcomes: $CD4^+$ and $CD8^+$ functionality score (FS), yellow fever-specific IgM and IgG antibody titer, neutralizing antibody titer, and frequency of antigen-specific $CD4^+$ and $CD8^+$ measured by intracellular cytokine staining for $CD40L^+IFN\gamma^+$ and $IFN\gamma^+TNF\alpha^+$, respectively, with CMV infection status (A, $n = 110$ CMV negative and $n = 49$ CMV positive) and sex (D, $n = 105$ female and $n = 56$ male).

(B) Boxplot comparison depicting the neutralizing and antigen-specific $CD4^+$ and $CD8^+$ response in CMV-positive ($n = 81$ for neutralizing titer and $n = 62$ for $CD8^+$ and $CD4^+$ responses) and negative ($n = 164$ for neutralizing titer and $n = 136$ for $CD8^+$ and $CD4^+$ responses) individuals.

(C) Boxplot comparison of CMV-positive or negative female and male individuals for the $CD8^+$ FS ($n = 43$ CMV-negative males, $n = 17$ CMV-positive males, $n = 83$ CMV-negative females, and $n = 42$ CMV-positive females) and the frequency of antigen-specific $CD8^+$ $IFN\gamma^+TNF\alpha^+$ T cells ($n = 47$ CMV-negative males, $n = 19$ CMV-positive males, $n = 89$ CMV-negative females, and $n = 43$ CMV-positive females).

(E) Boxplot comparison depicting the neutralizing and antigen-specific $CD4^+$ and $CD8^+$ response between both sexes ($n = 168$ female and $n = 79$ male for neutralizing titer and $n = 133$ female and $n = 67$ male for $CD8^+$ and $CD4^+$ responses). Radar plots were built using scaled data, and units refer to standard deviations from the mean. For IgG and IgM titers, data were scaled separately for TBEV pre-vaccinated and non-vaccinated groups. Boxplots show a horizontal line indicating the median and lower and upper hinges corresponding to the first and third quartiles. The lower and upper whiskers extend to 1.5x IQR from the respective hinge.

Statistical significance was assessed with Student's t test for radar plots and with Wilcoxon rank-sum test for boxplot comparisons and is depicted as ns (non-significant), $*p \leq 0.05$, $**p \leq 0.01$, $***p \leq 0.001$, $****p \leq 0.0001$.

See also Figure S6.

unaffected by CMV infection (Figures 3A–3C). On the other hand, sex alone did not affect any of the vaccination endpoints 28 days after vaccination, and females and males responded with equal strength to YF17D vaccination (Figures 3D and 3E).

Similar to the previous analysis, we conducted a multivariate linear regression model using sex, age, and the 20 infections as independent variables against 105 factors related to vaccination outcome. These factors included cytokine concentrations on days 3 and 7 after vaccination, the functionality and quantification of antigen-specific $CD4^+$ and $CD8^+$ T cells, the number and functionality of T cell memory subpopulations, and antibody and neutralization titers (Table S1C). Additionally, we evaluated the potential linear relationship between the antibody titer against the different antigens used for serodiagnosis in previously infected individuals and vaccination outcome. Notably, we did not identify any significant effect on the YF17D vaccine response (data not shown).

Altogether, even though CMV and sex had a significant influence on baseline immune distributions, they did not independently enhance or hamper the immunogenicity of the YF17D vaccine.

Classification of vaccine responses based on clustering of vaccination endpoints shows that sex and prior infections do not influence YF17D vaccine responses

Grouping individuals based on their vaccine response in good and weak responders improves the precise identification of factors that impact vaccine responses by minimizing interference from average responders. The YF17D vaccine is highly immunogenic, and all study participants developed neutralizing antibodies[35] and antigen-specific T cells by day 28 post immunization (Figure S6), which poses a challenge to separate the cohort into good and weak responders in a non-arbitrary manner. To address this, we performed hybrid hierarchical k-means

CellPress
OPEN ACCESS

clustering of the three main vaccination endpoints: the frequency and functionality of YF17D-specific CD4$^+$ and CD8$^+$ T cells, and the neutralizing antibody titer (Figure 4A). For CD4$^+$ T cells, 4 clusters discretely separated the cohort into elite, good, average, and poor responders. Similarly, for CD8$^+$ and neutralizing antibody titers, 3 clusters divided the cohort into good, average, and poor responders. Finally, individuals present in the good responder groups for every endpoint were classified as vaccine elite responders (n = 18), and individuals with poor responses in all endpoints were classified as vaccine poor responders (n = 17) (Figure 4B). Similarly, individuals classified as good responders in two of the indicators and average or good in the third were grouped as vaccine good responders (n = 49), while those classified as poor responders in two categories and average or poor in the third were named vaccine weak responders (n = 37) (Figure 4B).

Both sexes, CMV-infected and uninfected, and TBEV-vaccinated and unvaccinated individuals were equally distributed across the good and weak responder groups (χ^2 p value = 0.72, 0.40, and 0.72, respectively). Similarly, individuals are equally distributed according to CMV status and sex across antibody and CD4$^+$ and CD8$^+$ clusters (Figures 4C, 4D, and S7). This finding corroborates that none of these parameters independently influenced the main vaccination endpoints. Additionally, the accumulation of past infections or the sum of herpesvirus infections did not affect the response to the YF17D vaccine (Figures S8A–S8C).

Collectively, we did not find a direct association between any of the independent factors (sex, CMV, TBEV vaccination status, or prior infections) and the response to the YF17D vaccine on day 28.

Impact of HLA genotype on the YF17D vaccination outcome

To explore potential host genetic effects on the vaccination response, we mapped associations between HLA allele variation and vaccination outcomes. 13 alleles were associated with at least one vaccine outcome, but no association remains significant after multiple testing correction. Specifically, HLA-DPB1*03:01 associates with higher frequencies of antigen-specific CD8$^+$ T cells and good vaccine responders and has a positive slope for all vaccination endpoints. Likewise, the highly prevalent HLA-A*02:01 is positively associated with higher frequency and functionality of CD8$^+$ T cell responses and with good vaccine responders (Figures S9A–S9C). These results suggest that the HLA haplotype, which is functionally linked to adaptive cellular immunity, can play a role in shaping the immune response to the YF17D vaccine.

Baseline parameters affecting the response to the YF17D vaccine

Basal immune parameters can define different immunological states that condition the course of the vaccine response. Therefore, we explored whether baseline parameters, either individually or in combination, could predict the outcome of YF17D vaccination. Initially, we implemented a multivariate linear regression model using the 768 baseline parameters as dependent variables and the pre-defined vaccine responder groups as the independent variable (see STAR Methods and Table S2).

Interestingly, higher plasma levels of sIL6Ra as well as CXCL10 and SIGLEC-1 expression on non-classical monocytes (monocyte 2) were negatively associated with vaccine responsiveness (Figures 5A and 5B). This finding suggests a potential detrimental effect of basal interferons and inflammation on vaccine immunogenicity. To gain insight into the effect of a baseline interferon signature in the study cohort, we divided the vaccinees into quartiles based on their baseline levels of CXCL10 in plasma. Participants in the lowest quartile had significantly higher antigen-specific CD4$^+$ and CD8$^+$ T cell responses after vaccination compared to those in the highest quartile (Figures 5C and 5D). Thus, the baseline level of CXCL10, a prototypical interferon-stimulated protein detectable in plasma, was associated with decreased vaccine immunogenicity.

The frequency of baseline cytokine-expressing CD4$^+$ T cells predicts the outcome of YF17D vaccination

Good vaccine responders showed elevated counts of DCs (plasmacytoid, transitional, and classical) (Figure 5E) as well as non-classical monocytes, frequency of CCR4$^+$ Tregs, and of pre-switched B cells at baseline (Figure 5A). Among all factors, higher frequencies of cytokine-expressing (activated) CD8$^+$ and CD4$^+$ T cells at baseline showed the strongest link to good vaccine responses (Figure 5A, depicted in red and 5E). Independently, the frequency of activated CD4$^+$ T cells at baseline (such as CD4$^+$ T cells expressing TNFα^+, CD40L$^+$ IFNγ^+, CD40L$^+$TNFα, or IFNγ^+IL-2$^+$) was higher in good vaccine responders (Figure 5E).

To identify combinatorial baseline predictive patterns, individuals were grouped into 4 clusters based on high, average-high, average-low, and low frequencies of cytokine-producing CD4$^+$ T cells at baseline (Figure 6A). Those in the high-basal activation cluster significantly associated with elite and good vaccine responder groups as well as with the good YF17D-induced CD4$^+$ and CD8$^+$ response categories defined in Figure 4A (χ^2 p value = 0.0006, 0.0001, 0.0027, and 0.06, respectively) (Figure 6B). Additionally, individuals with a high frequency of cytokine-expressing CD4$^+$ T cells were associated with stronger YF-specific CD4$^+$, CD8$^+$, IgM, and neutralizing antibody responses (Figure 6C).

Overall, baseline activated CD4$^+$ T cell frequency serves as a robust positive predictor of both humoral and cellular responses to the YF17D vaccine.

Immunological factors associated with the activated CD4$^+$ T cell signature enhancing YF17D vaccine responses

We next aimed to identify immune features present at baseline that were associated with the frequency of cytokine-expressing CD4$^+$ T cells. To achieve this, we implemented another linear regression model using the clusters found in Figure 6A, examining individuals with high versus average-low and low frequency of baseline CD4$^+$ activation as independent variables and the 768 baseline parameters as dependent variables. Interestingly, the high basal CD4$^+$ activation state was significantly

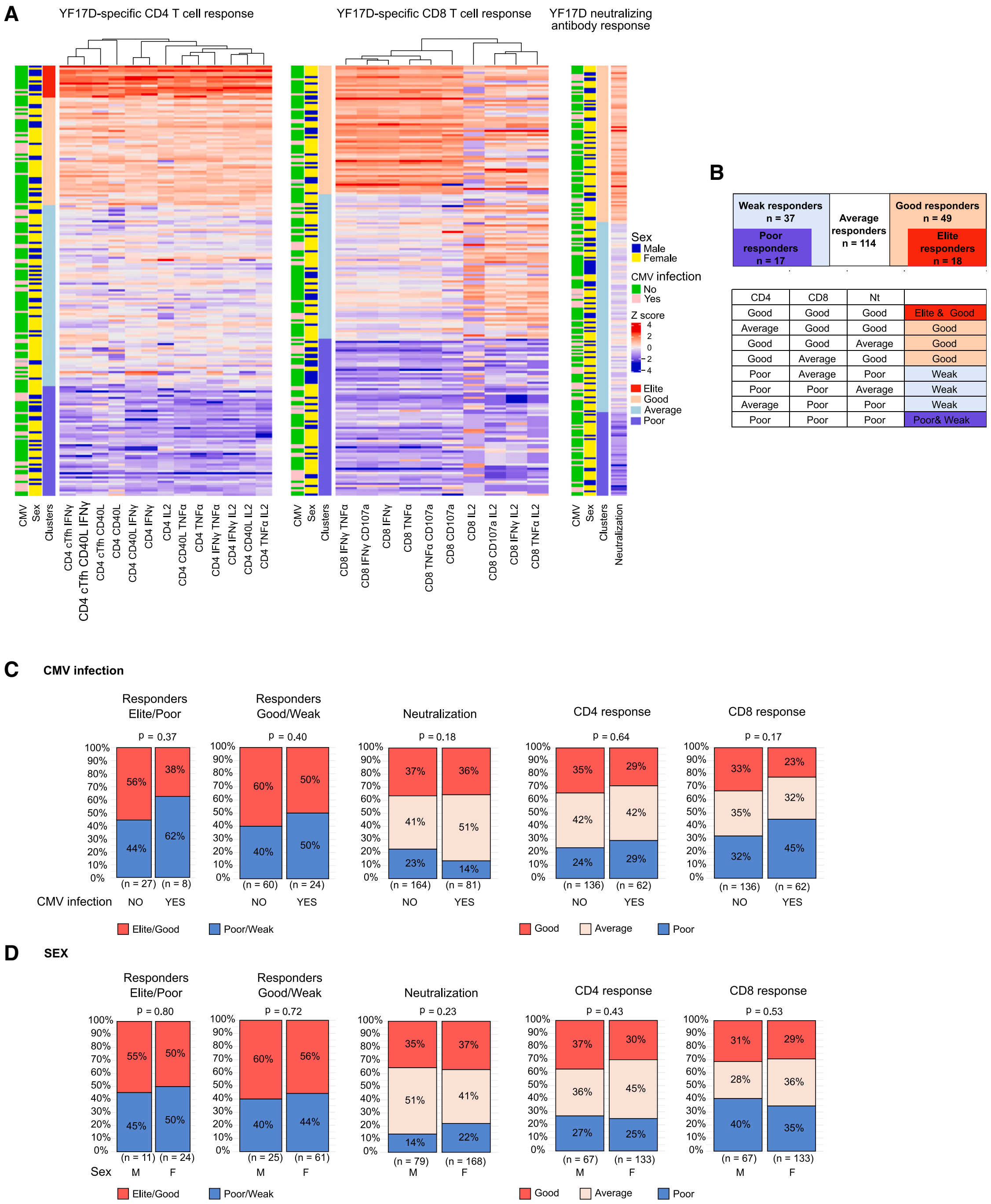

Figure 4. Vaccine response categories defined by clustering of vaccination endpoints

(A) Hybrid hierarchical k-means clustering of main vaccination endpoints: frequency of antigen-specific CD4$^+$ T cells, frequency of CD8$^+$ T cells and neutralizing antibody titers. 3 to 4 clusters identify good, average, and poor responders in each category. Every row represents an individual.

(B) Cohort grouping into 5 categories based on the clusters identified by clustering in (A).

(C and D) Response group allocation for CMV-infected and uninfected individuals (C) and for female and male sex (D). The sample size is indicated below each comparison and statistical significance was evaluated with a chi-square test.

See also Figures S7–S9.

CellPress
OPEN ACCESS

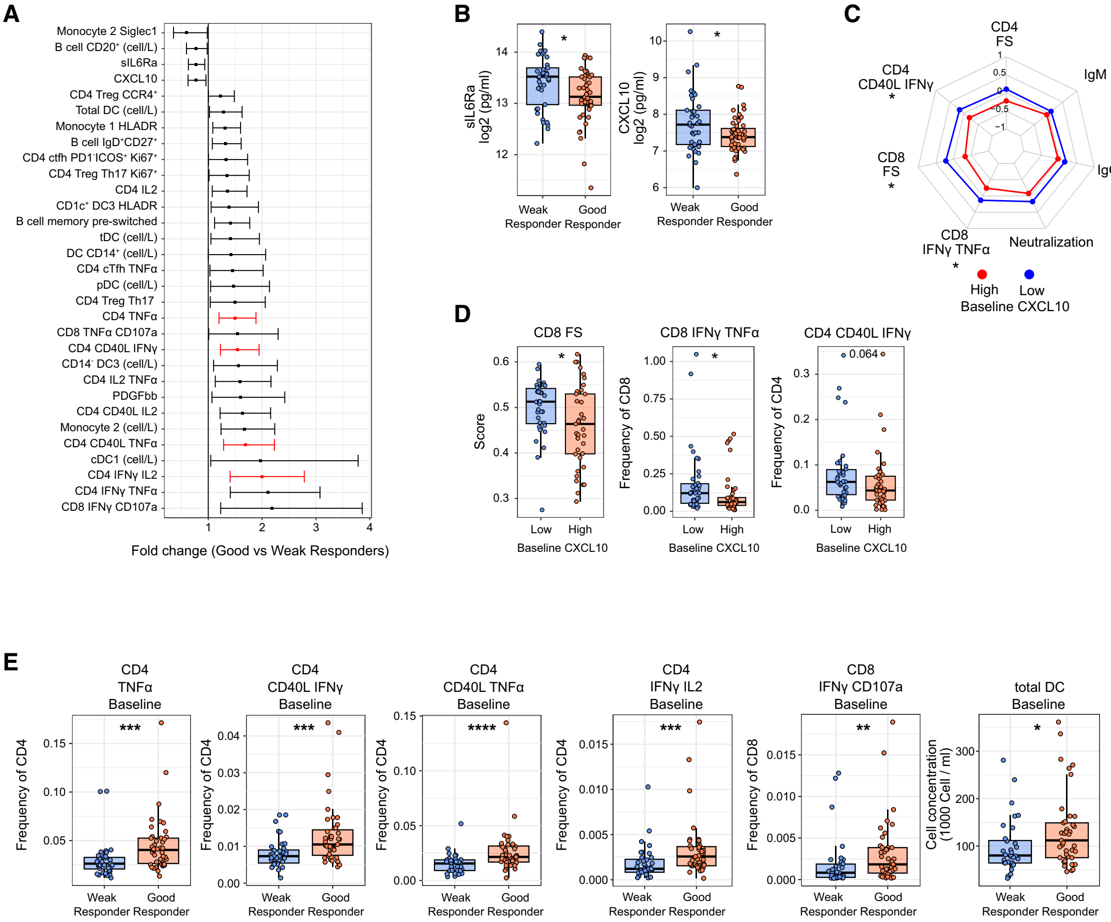

Figure 5. Baseline immune parameters associated with YF17D vaccination outcome

(A) Effect of baseline parameters on YF17D vaccine responses estimated by multivariate linear regression for good and weak responders (defined in Figure 4B). Data reflect the fold change between good/weak responders for the variables with a p value < 0.05. Associations with adjusted p value < 0.1 are depicted in red. Horizontal bars indicate the 2.5%–97.5% confidence intervals.

(B) Plasma levels of CXCL10 and sIL6Ra at baseline in good (n = 48) and weak (n = 37) vaccine responders (as defined in Figure 4B).

(C) Radar plot of seven scaled and continuous vaccine response endpoints for the individuals in the high (n = 34) and low (n = 28) quantiles of CXCL10 concentrations in plasma at baseline.

(D) T cell response endpoints for the high and low quantiles of CXCL10 concentration in plasma (n = 41 individuals in high and n = 38 individuals in low quantile).

(E) Frequency of activated $CD4^+$ T cells at baseline ($TNF\alpha^+$, $CD40L^+IFN\gamma^+$, $CD40L^+TNF\alpha^+$, and $IFN\gamma^+IL\text{-}2^+$), activated $CD8^+$ T cells ($IFN\gamma^+CD107a^+$), and total DC cell concentration in circulation in good (n = 43) and weak (n = 36) vaccine responders. Boxplots show a horizontal line indicating the median and lower and upper hinges corresponding to the first and third quartiles. The lower and upper whiskers extend to 1.5x IQR from the respective hinge. Radar plots were built using scaled data and units refer to standard deviations from the mean.

Statistical significance was assessed with Student's t test for radar plots and with Wilcoxon rank-sum test for boxplot comparisons and depicted as ns (non-significant), $*p \leq 0.05$, $**p \leq 0.01$, $***p \leq 0.001$, $****p \leq 0.0001$.

See also Table S2 for the linear model results.

associated with a high frequency of $CD4^+$ T cells that are differentiated ($CXCR3^+$, $CCR4^+$, $CCR6^+$, and EM, CCR7-$CD45RA^-$) and with a low frequency of naive and CXCR3-$CCR6^-$ $CD4^+$ T cells (Figure 7A). The proportion of $CCR4^+$ Tregs and cTfh of the total $CD4^+$ T cell population as well as the frequency of $Tbet^{low}Eomes^+$ $CD8^+$ T cells and pre-switched memory ($CD27^+IgM^+$) B cells were also associated with higher frequency of cytokine-expressing $CD4^+$ T cells (Figure 7A).

The clustering of $CD4^+$ populations identified in Figure 7A separated individuals with high and low frequencies of differentiated $CD4^+$ T cells (Figure 7B). As expected, individuals with a higher proportion of differentiated $CD4^+$ T cells exhibited higher neutralizing titers, greater $CD4^+$ FS, and increased numbers of YF17D-specific $CD4^+$ and $CD8^+$ T cell responses (Figure 7C). These individuals were also associated with strong vaccine responses (Figures 7D and 7E). The correlation of baseline parameters associated with strong vaccine responses revealed a profile characterized by a high frequency of differentiated cytokine-expressing $CD4^+$ T cells, an elevated proportion of memory B cells among total circulating $CD19^+$ cells, and a higher cell concentration of DCs, along with low levels of CXCL10, as the common baseline profile for good responders (Figure S10).

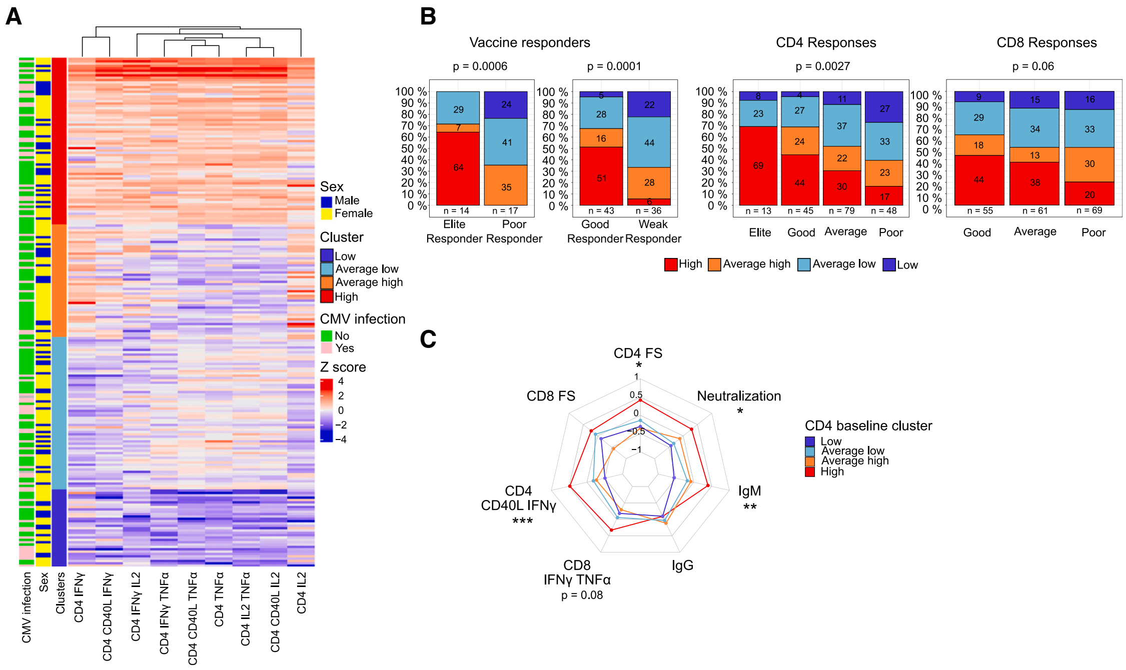

Figure 6. Frequency of baseline cytokine-expressing CD4+ T cells predicts YF17D vaccination outcome

(A) Hybrid hierarchical k-means clustering of the frequency of cytokine-expressing CD4+ T cell populations at baseline. 4 clusters define individuals with high CD4+ inflammatory status (n = 71), average-high (n = 48), average-low (n = 65), and low (n = 33) inflammatory status.

(B) Distribution of individuals classified based on the baseline CD4+ inflammatory status in (A) across the vaccine response categories defined in Figure 4. Statistical significance is evaluated with a chi-square test and sample size is indicated below every comparison.

(C) Radar plot associating vaccination endpoints across the different pre-vaccination CD4+ activation clusters identified in (A) (n = 52 high, n = 27 average high, n = 50 average low, and n = 19 low). Radar plots were built using scaled data and units refer to standard deviations from the mean. Statistical significance was assessed with ANOVA. Statistical significance is depicted with the following designation: ns (non-significant), *$p \leq 0.05$, **$p \leq 0.01$, ***$p \leq 0.001$, ****$p \leq 0.0001$. See also Figures S10 and S11.

Environmental and behavioral factors associated with the activated CD4+ T cell signature enhancing YF17D vaccine responses

The accumulation of past infections, including the number of herpesvirus infections, did not explain the baseline CD4+ T cell phenotype (Figures S11A–S11C). Other conditions such as alcohol consumption, smoking, dietary habits, upbringing in urban or rural areas, allergies, medication use, or recent illnesses before recruitment were assessed next. We found that having had a symptomatic infection before inclusion in the study negatively associated with the abundance of differentiated CD4+ T cells and baseline CD4+ T cell activation but was not associated with weaker vaccine responses and only showed a trend toward reduced antigen-specific CD4+ FS (Figure 7F). Interestingly, smoking was associated with higher frequencies of differentiated and cytokine-expressing CD4+ T cells at baseline (Figure 7G). Consequently, smoking demonstrated a significant association with a robust neutralizing response after YF17D vaccination, although smokers were present among both good and weak vaccine responders (Figure 7G).

DISCUSSION

Host genetics, environment, and intrinsic factors influence the basal immune status of healthy immunocompetent individuals and modify their response to infections and vaccinations. The live-attenuated YF17D vaccine virus is an excellent model for studying acute viral infections that result in long-lasting protective immunity. Understanding the environmental and host factors that influence YF17D immunogenicity in healthy young adults could provide valuable insights applicable to other immunological challenges, including natural infections.

In our analysis of past infections on baseline immune parameters and the response to a live vaccine, only CMV infection resulted in substantial variation of circulating immune cell types in young adults. Other studies have shown that EBV-specific T cells can accumulate and exhibit a less-differentiated phenotype compared to CMV-specific T cells.[2,11,13] Nevertheless, possibly due to the young age of our cohort and the high incidence of EBV (85%), we did not observe an EBV-associated effect.[18] Sex assigned at birth is a major factor influencing immune function and shaping the distribution of immune cell populations.[6,37,38] Previous trials found that males tend to have stronger antibody and neutralizing responses to YF-17D[39]; however, the effect of sex remains unclear across different studies.[39–42] In our cohort, females had higher average levels of immunoglobulin and naive T cells. Despite the sex- and CMV-driven differences in basal immune profiles, the YF17D vaccine responses were equally strong in these groups, with only a slightly blunted YF17D-specific CD8+ response in CMV-infected male donors.

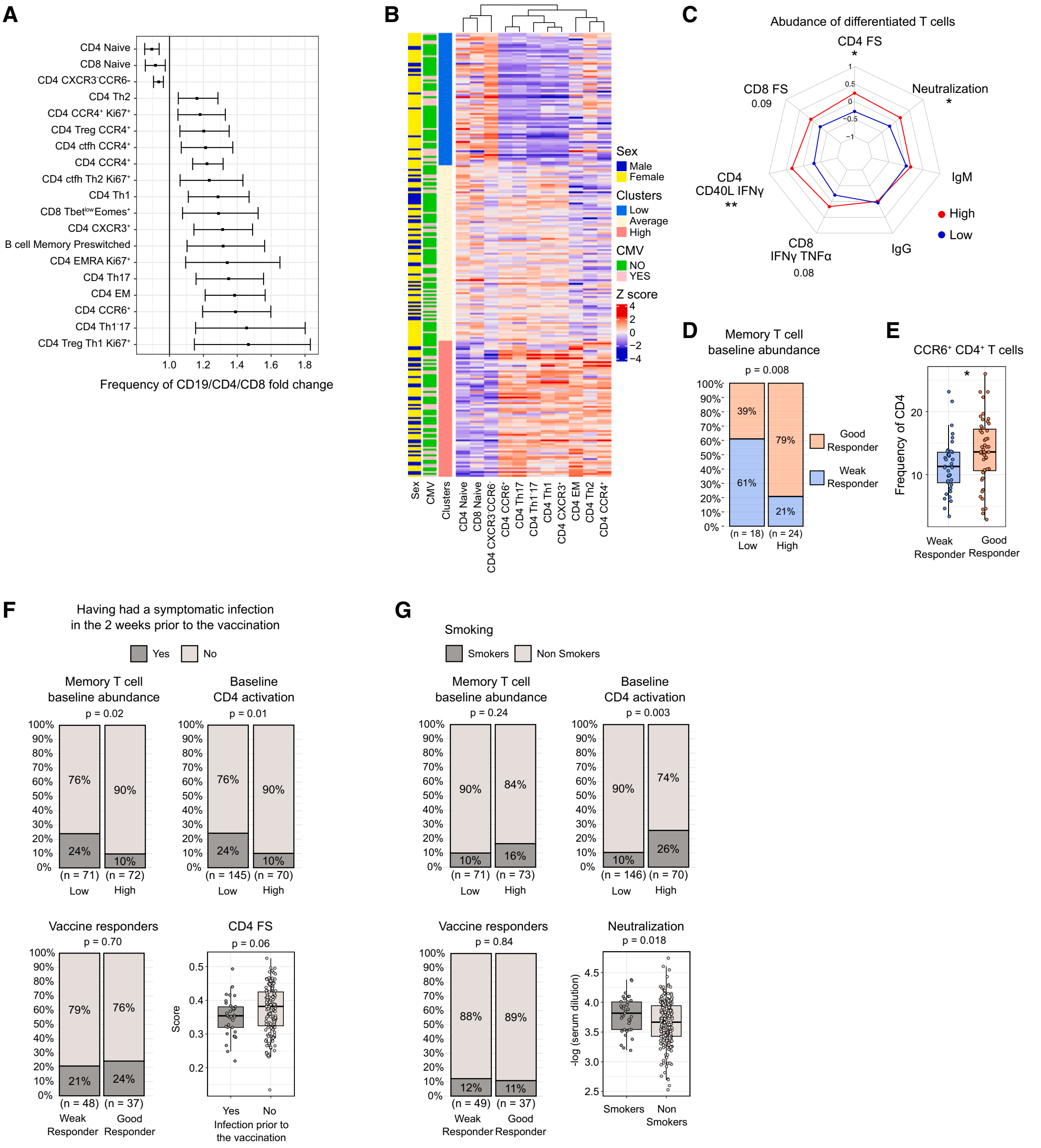

Figure 7. Immunological, environmental, and behavioral factors associated with the activated CD4⁺ T cell signature enhancing YF17D vaccine responses

(A) Multivariate linear regression analysis of the 768 baseline immune parameters with the CD4⁺ baseline cytokine expression clusters identified in Figure 6 (high versus average-low and low). Fold change is shown only for comparisons passing a multiple testing correction. Horizontal bars indicate the 2.5%–97.5% confidence intervals.

(B) Hybrid hierarchical k-means of the T cell memory populations identified in (A) to be associated with baseline cytokine-expressing CD4⁺ T cells. 3 clusters identify participants with a high frequency of differentiated CD4⁺ T cells (n = 71), with average (n = 94), and with low abundance of differentiated but high of naive T cells (n = 73).

(C) Radar plot associating the clusters identifies in (B) with the main vaccine outcome endpoints (n = 47 high and n = 45 low).

(D) Grouping of individuals with high/low abundance of differentiated T cells within the good and weak responder classification defined in Figure 4.

(E) Direct comparison of the frequency of CCR6⁺ CD4⁺ T cells between weak (n = 37) and good (n = 47) vaccine responders.

(F) The effect of symptomatic infection in the 2 weeks before YF17D vaccination on the response to the YF17D vaccine. Distribution of individuals with or without a symptomatic infection across clusters defined by the abundance of differentiated T cells (B), baseline activated CD4⁺ T cells (see Figure 6A), good and weak responders (see Figure 4B), and a direct comparison of the FS of YF17D-specific CD4⁺ T cells at day 28 between individuals with (n = 34) or without (n = 151) a symptomatic infection in the 2 weeks before YF17D vaccination.

(legend continued on next page)

This contrasts with previous studies reporting a beneficial effect of CMV infection on influenza vaccination in a healthy young cohort.[15] Other studies have shown that age and CMV infection go in concert with reduced responses to influenza and TBEV vaccines.[14,43] In our study, young vaccinees seemed to retain the capacity to respond vigorously to YF17D despite being positive for CMV, but it is possible that the effects of CMV infection on YF17D vaccine responses might only become apparent with increasing age. We hypothesize that CMV can exert a greater influence on the immunogenicity of inactivated vaccines whereas the high intrinsic immunogenicity of the YF17D vaccine, which consists of a replication-competent virus, may mitigate the effects of CMV and sex on vaccine responses.

Genetics, intrinsic, and environmental factors shape a basal immunological signature that is variable across individuals and indicative of particular immune states.[23] Therefore, it is possible to identify patterns in the basal immune status that identify a subject's predisposition to mount a strong immune response. Our analysis combined discrete classification models with regression-based models to find linear associations between baseline parameters and immune responses. Previously, we identified that TBEV pre-vaccination influences the epitope specificity of the humoral response to YF17D, significantly affecting the IgG antibody titers.[35] Nevertheless, our analysis of YF17D immunogenicity was not confounded by TBEV pre-vaccination, as all vaccination endpoints (e.g., good/weak responders, neutralizing antibody titers, and the number of YF-specific T cells) did not differ between TBEV-vaccinated and unvaccinated individuals, except for IgG titers, which were corrected by scaling both groups separately.[37] Unlike studies, focusing solely on neutralizing antibody titers,[23,29] we grouped individuals by both polyfunctional antigen-specific CD4$^+$ and CD8$^+$ responses as well as the neutralizing antibody levels separating good and weak responders. It is important to remark that all study participants, even those classified as poor responders, seroconverted and developed T cell and neutralizing antibody responses. Nevertheless, this grouping helps to reveal true biological differences between the good and weak vaccine responders.

Using these approaches, we successfully identified parameters associated with stronger YF17D vaccine responses: elevated baseline frequencies of cytokine-expressing CD4$^+$ T cells, higher CD8$^+$ T cell activation, and more abundant differentiated T cells, DCs, and pre-switched memory B cells. These indicators reflect the immune system's capacity to respond, aligning, in part, with previous studies on influenza vaccination.[23–26] Alternatively, higher frequencies of differentiated T cells in circulation could include increased numbers of naturally occurring YF-reactive T cells, which can exist even without an antigen exposure, potentially leading to robust T cell responses following immunization.[44] Conversely, baseline CXCL10 concentration in plasma was negatively associated with vaccine responses. CXCL10 levels might reflect an anti-viral state induced by interferons, which could limit the YF17D vaccine virus replication and thus its antigen dose and immunogenicity. Interestingly, live vaccines like YF17D seem to differ in the signatures associated with stronger humoral responses compared to other non-live vaccines that involve endotypes of immune activation, in innate cells like monocytes and DCs, and typically include interferon-stimulated genes and factors downstream of IRF7.[29]

Genetic variations, particularly in the HLA genes, have been shown to significantly influence adaptive immune responses to various vaccines, including hepatitis B, measles, influenza, and more recently to the severe acute respiratory syndrome coronavirus 2 vaccines.[4,45,46] In this study, we did not analyze genetic contributions to the vaccine response or basal immune variation in depth and only explored associations with the HLA locus, which is functionally linked to T cell immunity. We found that two prevalent HLA alleles, A*02:02 and DPB1*03:01, were positively associated with stronger responses to vaccination. These results highlight the importance of genetics also for the response to YF17D. The contribution of genetic factors and transcriptomic signatures are now the subject of further investigations in our cohort.

We have identified smoking as a factor not only linked to baseline CD4$^+$ T cell cytokine expression but also to the neutralizing response to the YF17D vaccine. Although our cohort only included 37 smokers, the association is consistent with previous studies.[39] This observation is likely attributed to its effects on adaptive immunity, such as augmenting the number of immune cells in circulation[47] and its effect on the epigenome and inflammation, which can result in long-lasting modulation of T cell activity.[48] Besides the link to smoking, the precise cause defining the pre-vaccination state could potentially arise from other non-infectious factors or subclinical pro-inflammatory responses that remain to be elucidated.

Collectively, our findings suggest that the exceptional performance of the YF17D vaccine may rely on its ability to be resilient against common interfering factors such as sex and CMV infection. Our findings support the idea that an active immunological state at baseline that does not impede the replication of a live attenuated virus results in a more effective vaccine response.

Limitations of the study

This study has inevitable limitations due to the characteristics of the cohort. The recruitment, conducted in a university hospital

(G) Effect of smoking on the response to the YF17D vaccine. Smoker distribution across clusters of the abundance of differentiated T cells (B), baseline activated CD4$^+$ T cells (see Figure 6A), good and weak responders, and a direct comparison with the neutralizing antibody titer (n = 37 smokers, n = 209 non-smokers).
Phenotypic markers of the following T cell populations named in the figure: naive (CD45RA$^+$CCR7$^+$CD95$^-$), CM (CD45RA-CCR7$^+$), EM (CD45RA-CCR7$^-$), cTfh (CXCR5$^+$), Treg (FoxP3$^+$CD25$^+$CD127 -), Th1 (CCR6 -CXCR3$^+$), Th17 (CCR6$^+$CXCR3$^-$), Th1-17 (CCR6$^+$CXCR3$^+$), and Th2 (CCR6-CXCR3-CCR4$^+$). Phenotypic markers of the following B cell populations named in the figure: memory pre-switched (CD27$^+$IgD-IgM$^+$). Boxplots show a horizontal line indicating the median and lower and upper hinges corresponding to the first and third quartiles. The lower and upper whiskers extend to 1.5x IQR from the respective hinge. Radar plots were built using scaled data and units refer to standard deviations from the mean.
Statistical significance was assessed with Student's t test for radar plots, chi-square for categorical group comparisons, and with Wilcoxon rank-sum test for boxplot comparisons and depicted as ns (non-significant), $*p \leq 0.05$, $**p \leq 0.01$, $***p \leq 0.001$, $****p \leq 0.0001$.
See also Table S2 for the linear model results and Figures S10 and S11.

setting, introduced a bias toward young students with healthy lifestyle habits, resulting in 84.8% of participants having a normal body mass index and 80.4% being aged 20–29 years. These characteristics limited our ability to evaluate the impact of these factors, and the findings presented here might not be generalizable to older populations. Additionally, certain pathogens had high prevalence rates within the cohort, such as EBV (84.7%) and WVPyV (96.7%), making it suboptimal for assessing their influence on baseline immune variation and vaccine responses. Lastly, the evaluation of vaccine responses is limited to day 28 post vaccination. While this time point is sufficient for the development of protective cellular and humoral immunity, it does not allow us to assess the impact of the tested parameters on antibody maturation or the durability of immune memory.

RESOURCE AVAILABILITY

Lead contact

Further information and requests for resources and reagents should be directed to and will be fulfilled by the lead contact, Prof. Dr. Simon Rothenfusser (simon.rothenfusser@med.uni-muenchen.de).

Materials availability

This study did not generate new unique reagents.

Data and code availability

- Data reported in this paper will be shared by the lead contact upon reasonable request. Information on the study cohort can be found in the ISRCTN registry, nr. 17974967.[49] Study protocol is available upon request.
- This paper does not report original code. All custom code used for the analyses was written with existing software as detailed in the methods section and is available upon request.
- Any additional information required to reanalyze the data reported in this paper is available from the lead contact upon request.

ACKNOWLEDGMENTS

The authors thank Natalie Roeder, Nicole Lichter, and Christine Hoerth for technical assistance. We thank Prof. Daniel Teupser and his coworkers from the Institute of Laboratory Medicine, LMU Munich, for performing blood tests. We acknowledge the iFlow Core Facility of the University Hospital Munich and the Core Facility Flow Cytometry of the Biomedical Center, LMU Munich, for assistance with the generation of flow cytometry data and thank Lisa Richter and Pardis Khosravani. We thank Elfriede Noessner and Barbara Mosetter from the immunoanalytics platform of the Helmholtz Center Munich for their help with cytokine measurements with the bioplex assay.

This work was supported by FlavImmunity, a combined grant of the German Research Foundation (DFG) project number 391217598 to S.R. and A.B.K. and the French National Research Agency (ANR) project number ANR-17-CE15-0031-01 to G.B.-S.; DFG TRR237 grant project number 369799452 to S.R. and A.B.K. (TRR237 TPB14); the Yellow4FLAVI project, funded by the European Union, under the Horizon Europe Program, grant agreement no. 101137459 to S.R., A.K., and G.B.-S.; DFG SFB1054 TPA06 project number 210592381 to A.B.K.; grants of the iMed consortium of the German Helmholtz Societies to S.R.; the Einheit für Klinische Pharmakologie (EKLIP), Helmholtz Zentrum München, Neuherberg, Germany to S.R. and S.E.; grants by the Friedrich Baur Foundation (FBS) to H.K. and M.P.; a Metiphys fellowship of the Medical Faculty of the LMU Munich to M.P.; the FöFoLe Program of the Medical Faculty of the LMU Munich to S.G., E.N., L.L., M.K.S., and S.J.; and the international doctoral program "iTarget: Immunotargeting of cancer" funded by the Elite Network of Bavaria to A.S.-P., M.Z., S.G., L.L., and E.N. and the doctoral program iTarget 2.0 to A.S.-P., M.Z., S.G., L.L., E.N., and S.J. The study was funded by the European Union. Views and opinions expressed are, however, those of the authors only and do not necessarily reflect those of the European Union or HADEA. The funders had no role in study design, data collection and analysis, decision to publish, or preparation of the manuscript.

AUTHOR CONTRIBUTIONS

Conceptualization, A.S.-P. and S.R.; methodology, A.S.-P., M.Z., E.N., E.W., T.W., J.B., and C.M.-Z.; investigation, A.S.-P., M.Z., E.N., E.W., S.G., M.K.S., F.D., F.L., S.J., and L.L.; formal analysis, A.S.-P., M.Z., E.N., E.Y., and B.P.; writing – original draft, A.S.-P.; writing – review and editing, A.S.-P. and S.R.; resources, T.W., J.T.-S., M.P., G.B.-S., and S.R.; funding acquisition, S.E., H.K., M.P., G.B.-S., A.B.K., and S.R.; supervision, G.B.-S., S.K.-H., A.B.K., S.E., and S.R.

DECLARATION OF INTERESTS

The authors declare no competing interests.

STAR★METHODS

Detailed methods are provided in the online version of this paper and include the following:

- KEY RESOURCES TABLE
- EXPERIMENTAL MODEL AND STUDY PARTICIPANT DETAILS
  - Cohort and samples
- METHOD DETAILS
  - HLA typing
  - Serological analysis
  - YF17D and YF17D-Venus virus production
  - Yellow fever neutralization
  - Cytokine measurement
  - T cell re-stimulation with YF-17D virus
  - Intracellular staining of *ex vivo* re-stimulated samples
  - COMPASS analysis
  - Flow cytometry and phenotyping
- QUANTIFICATION AND STATISTICAL ANALYSIS
  - Multivariate linear regression analysis
  - Batch correction
  - Hybrid hierarchical k-means clustering
  - Principal component analysis of baseline parameters
  - Other statistics
- ADDITIONAL RESOURCES

SUPPLEMENTAL INFORMATION

Supplemental information can be found online at https://doi.org/10.1016/j.xcrm.2025.101946.

Received: January 24, 2024
Revised: September 19, 2024
Accepted: January 15, 2025
Published: February 11, 2025

REFERENCES

1. Zimmermann, P., and Curtis, N. (2019). Factors That Influence the Immune Response to Vaccination. Clin. Microbiol. Rev. *32*, 10–1128. https://doi.org/10.1128/CMR.00084-18.

2. Patin, E., Hasan, M., Bergstedt, J., Rouilly, V., Libri, V., Urrutia, A., Alanio, C., Scepanovic, P., Hammer, C., Jönsson, F., et al. (2018). Natural variation in the parameters of innate immune cells is preferentially driven by genetic factors. Nat. Immunol. *19*, 302–314. https://doi.org/10.1038/s41590-018-0049-7.

3. Scepanovic, P., Alanio, C., Hammer, C., Hodel, F., Bergstedt, J., Patin, E., Thorball, C.W., Chaturvedi, N., Charbit, B., Abel, L., et al. (2018). Human genetic variants and age are the strongest predictors of humoral immune responses to common pathogens and vaccines. Genome Med. *10*, 59. https://doi.org/10.1186/s13073-018-0568-8.

4. Mentzer, A.J., O'Connor, D., Bibi, S., Chelysheva, I., Clutterbuck, E.A., Demissie, T., Dinesh, T., Edwards, N.J., Felle, S., Feng, S., et al. (2023). Human leukocyte antigen alleles associate with COVID-19 vaccine immunogenicity and risk of breakthrough infection. Nat. Med. *29*, 147–157. https://doi.org/10.1038/s41591-022-02078-6.

5. Linnik, J.E., and Egli, A. (2016). Impact of host genetic polymorphisms on vaccine induced antibody response. Hum. Vaccines Immunother. *12*, 907–915. https://doi.org/10.1080/21645515.2015.1119345.

6. Klein, S.L., and Flanagan, K.L. (2016). Sex differences in immune responses. Nat. Rev. Immunol. *16*, 626–638. https://doi.org/10.1038/nri.2016.90.

7. López-Otín, C., Blasco, M.A., Partridge, L., Serrano, M., and Kroemer, G. (2013). The Hallmarks of Aging. Cell *153*, 1194–1217. https://doi.org/10.1016/j.cell.2013.05.039.

8. Goronzy, J.J., and Weyand, C.M. (2013). Understanding immunosenescence to improve responses to vaccines. Nat. Immunol. *14*, 428–436. https://doi.org/10.1038/ni.2588.

9. Tu, W., and Rao, S. (2016). Mechanisms Underlying T Cell Immunosenescence: Aging and Cytomegalovirus Infection. Front. Microbiol. 7, 2111.

10. Goodrum, F., Caviness, K., and Zagallo, P. (2012). Human cytomegalovirus persistence. Cell Microbiol. *14*, 644–655. https://doi.org/10.1111/j.1462-5822.2012.01774.x.

11. Colonna-Romano, G., Akbar, A.N., Aquino, A., Bulati, M., Candore, G., Lio, D., Ammatuna, P., Fletcher, J.M., Caruso, C., and Pawelec, G. (2007). Impact of CMV and EBV seropositivity on CD8 T lymphocytes in an old population from West-Sicily. Exp. Gerontol. *42*, 995–1002. https://doi.org/10.1016/j.exger.2007.05.006.

12. Sylwester, A.W., Mitchell, B.L., Edgar, J.B., Taormina, C., Pelte, C., Ruchti, F., Sleath, P.R., Grabstein, K.H., Hosken, N.A., Kern, F., et al. (2005). Broadly targeted human cytomegalovirus-specific CD4+ and CD8+ T cells dominate the memory compartments of exposed subjects. J. Exp. Med. *202*, 673–685. https://doi.org/10.1084/jem.20050882.

13. Derhovanessian, E., Maier, A.B., Hähnel, K., Beck, R., de Craen, A.J.M., Slagboom, E.P., Westendorp, R.G.J., and Pawelec, G. (2011). Infection with cytomegalovirus but not herpes simplex virus induces the accumulation of late-differentiated CD4+ and CD8+ T-cells in humans. J. Gen. Virol. *92*, 2746–2756. https://doi.org/10.1099/vir.0.036004-0.

14. van den Berg, S.P.H., Warmink, K., Borghans, J.A.M., Knol, M.J., and van Baarle, D. (2019). Effect of latent cytomegalovirus infection on the antibody response to influenza vaccination: a systematic review and meta-analysis. Med. Microbiol. Immunol. *208*, 305–321. https://doi.org/10.1007/s00430-019-00602-z.

15. Furman, D., Jojic, V., Sharma, S., Shen-Orr, S.S., Angel, C.J.L., Onengut-Gumuscu, S., Kidd, B.A., Maecker, H.T., Concannon, P., Dekker, C.L., et al. (2015). Cytomegalovirus infection enhances the immune response to influenza. Sci. Transl. Med. *7*, 281ra43, 281ra43-281ra43. https://doi.org/10.1126/scitranslmed.aaa2293.

16. McElhaney, J.E., Garneau, H., Camous, X., Dupuis, G., Pawelec, G., Baehl, S., Tessier, D., Frost, E.H., Frasca, D., Larbi, A., et al. (2015). Predictors of the antibody response to influenza vaccination in older adults with type 2 diabetes. BMJ Open Diabetes Research & Care *3*, e000140. https://doi.org/10.1136/bmjdrc-2015-000140.

17. Frasca, D., Diaz, A., Romero, M., Landin, A.M., and Blomberg, B.B. (2015). Cytomegalovirus (CMV) seropositivity decreases B cell responses to the influenza vaccine. Vaccine *33*, 1433–1439. https://doi.org/10.1016/j.vaccine.2015.01.071.

18. Trzonkowski, P., Myśliwska, J., Szmit, E., Wieckiewicz, J., Łukaszuk, K., Brydak, L.B., Machała, M., and Myśliwski, A. (2003). Association between cytomegalovirus infection, enhanced proinflammatory response and low level of anti-hemagglutinins during the anti-influenza vaccination—an impact of immunosenescence. Vaccine *21*, 3826–3836. https://doi.org/10.1016/S0264-410X(03)00309-8.

19. Goronzy, J.J., Fulbright, J.W., Crowson, C.S., Poland, G.A., O'Fallon, W.M., and Weyand, C.M. (2001). Value of Immunological Markers in Predicting Responsiveness to Influenza Vaccination in Elderly Individuals. J. Virol. *75*, 12182–12187. https://doi.org/10.1128/JVI.75.24.12182-12187.2001.

20. van den Berg, S.P.H., Wong, A., Hendriks, M., Jacobi, R.H.J., van Baarle, D., and van Beek, J. (2018). Negative Effect of Age, but Not of Latent Cytomegalovirus Infection on the Antibody Response to a Novel Influenza Vaccine Strain in Healthy Adults. Front. Immunol. *9*, 82. https://doi.org/10.3389/fimmu.2018.00082.

21. van den Berg, S.P.H., Lanfermeijer, J., Jacobi, R.H.J., Hendriks, M., Vos, M., van Schuijlenburg, R., Nanlohy, N.M., Borghans, J.A.M., van Beek, J., van Baarle, D., and de Wit, J. (2021). Latent CMV Infection Is Associated With Lower Influenza Virus-Specific Memory T-Cell Frequencies, but Not With an Impaired T-Cell Response to Acute Influenza Virus Infection. Front. Immunol. *12*, 663664. https://doi.org/10.3389/fimmu.2021.663664.

22. Huang, Y., Zhang, Y., Seaton, K.E., De Rosa, S., Heptinstall, J., Carpp, L.N., Randhawa, A.K., McKinnon, L.R., McLaren, P., Viegas, E., et al. (2022). Baseline host determinants of robust human HIV-1 vaccine-induced immune responses: A meta-analysis of 26 vaccine regimens. EBioMedicine *84*, 104271. https://doi.org/10.1016/j.ebiom.2022.104271.

23. Tsang, J.S., Schwartzberg, P.L., Kotliarov, Y., Biancotto, A., Xie, Z., Germain, R.N., Wang, E., Olnes, M.J., Narayanan, M., Golding, H., et al. (2014). Global Analyses of Human Immune Variation Reveal Baseline Predictors of Postvaccination Responses. Cell *157*, 499–513. https://doi.org/10.1016/j.cell.2014.03.031.

24. Tsang, J.S., Dobaño, C., VanDamme, P., Moncunill, G., Marchant, A., Othman, R.B., Sadarangani, M., Koff, W.C., and Kollmann, T.R. (2020). Improving Vaccine-Induced Immunity: Can Baseline Predict Outcome? Trends Immunol. *41*, 457–465. https://doi.org/10.1016/j.it.2020.04.001.

25. Kennedy, R.B., Simon, W.L., Gibson, M.J., Goergen, K.M., Grill, D.E., Oberg, A.L., and Poland, G.A. (2016). The composition of immune cells serves as a predictor of adaptive immunity in a cohort of 50- to 74-year-old adults. Immunology *148*, 266–275. https://doi.org/10.1111/imm.12599.

26. Kotliarov, Y., Sparks, R., Martins, A.J., Mulè, M.P., Lu, Y., Goswami, M., Kardava, L., Banchereau, R., Pascual, V., Biancotto, A., et al. (2020). Broad immune activation underlies shared set point signatures for vaccine responsiveness in healthy individuals and disease activity in patients with lupus. Nat. Med. *26*, 618–629. https://doi.org/10.1038/s41591-020-0769-8.

27. HIPC-CHI Signatures Project Team; HIPC-I Consortium (2017). Multicohort analysis reveals baseline transcriptional predictors of influenza vaccination responses. Sci. Immunol. *2*, eaal4656. https://doi.org/10.1126/sciimmunol.aal4656.

28. Fourati, S., Cristescu, R., Loboda, A., Talla, A., Filali, A., Railkar, R., Schaeffer, A.K., Favre, D., Gagnon, D., Peretz, Y., et al. (2016). Pre-vaccination inflammation and B-cell signalling predict age-related hyporesponse to hepatitis B vaccination. Nat. Commun. *7*, 10369. https://doi.org/10.1038/ncomms10369.

29. Fourati, S., Tomalin, L.E., Mulè, M.P., Chawla, D.G., Gerritsen, B., Rychkov, D., Henrich, E., Miller, H.E.R., Hagan, T., Diray-Arce, J., et al. (2022). Pan-vaccine analysis reveals innate immune endotypes predictive of antibody responses to vaccination. Nat. Immunol. *23*, 1777–1787. https://doi.org/10.1038/s41590-022-01329-5.

30. Hagan, T., Gerritsen, B., Tomalin, L.E., Fourati, S., Mulè, M.P., Chawla, D.G., Rychkov, D., Henrich, E., Miller, H.E.R., Diray-Arce, J., et al. (2022). Transcriptional atlas of the human immune response to 13 vaccines reveals a common predictor of vaccine-induced antibody

responses. Nat. Immunol. *23*, 1788–1798. https://doi.org/10.1038/s41590-022-01328-6.

31. Poland, J.D., Calisher, C.H., Monath, T.P., Downs, W.G., and Murphy, K. (1981). Persistence of neutralizing antibody 30-35 years after immunization with 17D yellow fever vaccine. Bull. World Health Organ. *59*, 895–900.
32. Fuertes Marraco, S.A., Soneson, C., Cagnon, L., Gannon, P.O., Allard, M., Abed Maillard, S., Montandon, N., Rufer, N., Waldvogel, S., Delorenzi, M., and Speiser, D.E. (2015). Long-lasting stem cell–like memory CD8+ T cells with a naïve-like profile upon yellow fever vaccination. Sci. Transl. Med. *7*, 282ra48. https://doi.org/10.1126/scitranslmed.aaa3700.
33. Monath, T.P., and Vasconcelos, P.F.C. (2015). Yellow fever. J. Clin. Virol. *64*, 160–173. https://doi.org/10.1016/j.jcv.2014.08.030.
34. Kling, K., Domingo, C., Bogdan, C., Duffy, S., Harder, T., Howick, J., Kleijnen, J., McDermott, K., Wichmann, O., Wilder-Smith, A., and Wolff, R. (2022). Duration of protection after vaccination against yellow fever: a systematic review and meta-analysis. Clin. Infect. Dis. *75*, 2266–2274, ciac580. https://doi.org/10.1093/cid/ciac580.
35. Santos-Peral, A., Luppa, F., Goresch, S., Nikolova, E., Zaucha, M., Lehmann, L., Dahlstroem, F., Karimzadeh, H., Thorn-Seshold, J., Winheim, E., et al. (2024). Prior flavivirus immunity skews the yellow fever vaccine response to cross-reactive antibodies with potential to enhance dengue virus infection. Nat. Commun. *15*, 1696. https://doi.org/10.1038/s41467-024-45806-x.
36. Lin, L., Finak, G., Ushey, K., Seshadri, C., Hawn, T.R., Frahm, N., Scriba, T.J., Mahomed, H., Hanekom, W., Bart, P.-A., et al. (2015). COMPASS identifies T-cell subsets correlated with clinical outcomes. Nat. Biotechnol. *33*, 610–616. https://doi.org/10.1038/nbt.3187.
37. Libert, C., Dejager, L., and Pinheiro, I. (2010). The X chromosome in immune functions: when a chromosome makes the difference. Nat. Rev. Immunol. *10*, 594–604. https://doi.org/10.1038/nri2815.
38. Furman, D., Hejblum, B.P., Simon, N., Jojic, V., Dekker, C.L., Thiébaut, R., Tibshirani, R.J., Davis, M.M., and David, F.,P.,H.B. (2014). Systems analysis of sex differences reveals an immunosuppressive role for testosterone in the response to influenza vaccination. Proc. Natl. Acad. Sci. USA *111*, 869–874. https://doi.org/10.1073/pnas.1321060111.
39. Monath, T.P., Nichols, R., Archambault, W.T., Moore, L., Marchesani, R., Tian, J., Shope, R.E., Thomas, N., Schrader, R., Furby, D., and Bedford, P. (2002). Comparative safety and immunogenicity of two yellow fever 17D vaccines (ARILVAX and YF-VAX) in a phase III multicenter, double-blind clinical trial. Am. J. Trop. Med. Hyg. *66*, 533–541. https://doi.org/10.4269/ajtmh.2002.66.533.
40. Pfister, M., Kürsteiner, O., Hilfiker, H., Favre, D., Durrer, P., Ennaji, A., L'age-Stehr, J., Kaufhold, A., and Herzog, C. (2005). Immunogenicity and Safety of Berna-YF Compared with two other 17D Yellow Fever Vaccines in Phase 3 Clinical Trial. The American Journal of Tropical Medicine and Hygiene Am J Trop Med Hyg *72*, 339–346. https://doi.org/10.4269/ajtmh.2005.72.339.
41. Veit, O., Niedrig, M., Chapuis-Taillard, C., Cavassini, M., Mossdorf, E., Schmid, P., Bae, H.-G., Litzba, N., Staub, T., Hatz, C., et al. (2009). Immunogenicity and Safety of Yellow Fever Vaccination for 102 HIV-Infected Patients. Clin. Infect. Dis. *48*, 659–666. https://doi.org/10.1086/597006.
42. Klein, S.L., Jedlicka, A., and Pekosz, A. (2010). The Xs and Y of immune responses to viral vaccines. Lancet Infect. Dis. *10*, 338–349. https://doi.org/10.1016/S1473-3099(10)70049-9.
43. Nicoli, F., Clave, E., Wanke, K., von Braun, A., Bondet, V., Alanio, C., Douay, C., Baque, M., Lependu, C., Marconi, P., et al. (2022). Primary immune responses are negatively impacted by persistent herpesvirus infections in older people: results from an observational study on healthy subjects and a vaccination trial on subjects aged more than 70 years old. EBioMedicine *76*, 103852. https://doi.org/10.1016/j.ebiom.2022.103852.
44. Pan, Y.-G., Aiamkitsumrit, B., Bartolo, L., Wang, Y., Lavery, C., Marc, A., Holec, P.V., Rappazzo, C.G., Eilola, T., Gimotty, P.A., et al. (2021). Vaccination reshapes the virus-specific T cell repertoire in unexposed adults. Immunity *54*, 1245–1256.e5. https://doi.org/10.1016/j.immuni.2021.04.023.
45. Smatti, M.K., Alkhatib, H.A., Al Thani, A.A., and Yassine, H.M. (2022). Will Host Genetics Affect the Response to SARS-CoV-2 Vaccines? Historical Precedents. Front. Med. *9*, 802312. https://doi.org/10.3389/fmed.2022.802312.
46. Xie, J., Mothe, B., Alcalde Herraiz, M., Li, C., Xu, Y., Jödicke, A.M., Gao, Y., Wang, Y., Feng, S., Wei, J., et al. (2024). Relationship between HLA genetic variations, COVID-19 vaccine antibody response, and risk of breakthrough outcomes. Nat. Commun. *15*, 4031. https://doi.org/10.1038/s41467-024-48339-5.
47. Pedersen, K.M., Çolak, Y., Ellervik, C., Hasselbalch, H.C., Bojesen, S.E., and Nordestgaard, B.G. (2019). Smoking and Increased White and Red Blood Cells. Arterioscler. Thromb. Vasc. Biol. *39*, 965–977. https://doi.org/10.1161/ATVBAHA.118.312338.
48. Saint-André, V., Charbit, B., Biton, A., Rouilly, V., Possémé, C., Bertrand, A., Rotival, M., Bergstedt, J., Patin, E., Albert, M.L., et al. (2024). Smoking changes adaptive immunity with persistent effects. Nature *626*, 827–835. https://doi.org/10.1038/s41586-023-06968-8.
49. Rothenfusser, S. (2023). Creating and studying a group of yellow fever-vaccinated individuals to discover factors affecting the immune response to vaccination and acute viral infections. ISRCTN. https://doi.org/10.1186/ISRCTN17974967.
50. Winheim, E., Rinke, L., Lutz, K., Reischer, A., Leutbecher, A., Wolfram, L., Rausch, L., Kranich, J., Wratil, P.R., Huber, J.E., et al. (2021). Impaired function and delayed regeneration of dendritic cells in COVID-19. PLoS Pathog. *17*, e1009742-.
51. Mentzer, A.J., Brenner, N., Allen, N., Littlejohns, T.J., Chong, A.Y., Cortes, A., Almond, R., Hill, M., Sheard, S., McVean, G., et al. (2022). Identification of host–pathogen-disease relationships using a scalable multiplex serology platform in UK Biobank. Nat. Commun. *13*, 1818. https://doi.org/10.1038/s41467-022-29307-3.
52. Zhou, X., and Stephens, M. (2012). Genome-wide efficient mixed-model analysis for association studies. Nat. Genet. *44*, 821–824. https://doi.org/10.1038/ng.2310.
53. Tan, J.L., and Lok, S.M. (2014). In Dengue Virus Purification and Sample Preparation for Cryo-Electron Microscopy BT - Dengue: Methods and Protocols, R. Padmanabhan and S.G. Vasudevan, eds. (Springer New York), pp. 41–52. https://doi.org/10.1007/978-1-4939-0348-1_4.
54. Scheck, M.K., Lehmann, L., Zaucha, M., Schwarzlmueller, P., Huber, K., Pritsch, M., Barba-Spaeth, G., Thorn-Seshold, O., Krug, A.B., Endres, S., et al. (2022). FluoRNT: A robust, efficient assay for the detection of neutralising antibodies against yellow fever virus 17D. PLoS One *17*, e0262149.
55. Hofmann, H., Heinz, F.X., and Dippe, H. (1983). ELISA for IgM and IgG antibodies against tick-borne encephalitis virus: Quantification and standardization of results. Zentralblatt für Bakteriol. Mikrobiol. Hyg. 1. Abt. Orig. Med. Mikrobiol. Infekt. Parasitol. *255*, 448–455. https://doi.org/10.1016/S0174-3031(83)80002-X.

STAR★METHODS

KEY RESOURCES TABLE

REAGENT or RESOURCE	SOURCE	IDENTIFIER
Antibodies		
Anti-human IgG HRP	Jackson ImmunoResearch	Cat# 109-035-088; RRID:AB_2337584
Anti-human IgM HRP	ThermoFisher	Cat# 31415; RRID:AB_228282
Fluorochrome-conjugated antibodies for antigen-presenting cell populations, DC and monocytes	Winheim et al.[50]	NA
BUV395-CD45RA	BD	Cat# 740298; RRID:AB_2740037
BUV496-CD16	BD	Cat# 612944; RRID:AB_2870224
BUV563-CD127	BD	Cat# 748489; RRID:AB_2872902
BUV661-CCR6	BD	Cat# 750696; RRID:AB_2874817
BUV737-PD1	BD	Cat# 612792; RRID:AB_2870119
BUV805-ICOS	BD	Cat# 748903; RRID:AB_2873306
Brilliant Violet™ 480-CD56	BD	Cat# 566162; RRID:AB_2739559
Brilliant Violet™ 510-CD4	BioLegend	Cat# 317444; RRID:AB_2561866
Brilliant Violet™ 570-HLA-DR	BioLegend	Cat# 307637; RRID:AB_10895753
Brilliant Violet™ 605-CXCR3	BioLegend	Cat# 353728; RRID:AB_2563157
Pacific Blue™-CD57	BioLegend	Cat# 322316; RRID:AB_2063197
Brilliant Violet™ 650-Ki67	BD	Cat# 563757; RRID:AB_2688008
Brilliant Violet™ 711-KLRG1	BioLegend	Cat# 138427; RRID:AB_2629721
Brilliant Violet™ 750-CXCR5	BioLegend	Cat# 356941; RRID:AB_2832703
Brilliant Violet™ 785-CCR7	BioLegend	Cat# 353229; RRID:AB_2561371
FITC-Tbet	BioLegend	Cat# 644811; RRID:AB_2287097
Spark Blue™ 550-CD3	BioLegend	Cat# 344851; RRID:AB_2819984
BB700-CD25	BD	Cat# 566448; RRID: AB_2744335
PE/Cyanine7-CCR4	BioLegend	Cat# 359410; RRID:AB_2562431
PE-TCF1	BioLegend	Cat# 655207; RRID:AB_2728491
PE/Dazzle 594-FoxP3	BioLegend	Cat# 320126; RRID:AB_2564025
PE/Cyanine5-Eomes	Thermofisher	Cat# 15-4875-80; RRID:AB_2802206
Alexa Fluor® 700-CD8	BioLegend	Cat# 300920; RRID:AB_528885
APC Fire 810-CD38	BioLegend	Cat# 356644; RRID: AB_2860936
APC/Fire™ 750-CD95	BioLegend	Cat# 305638; RRID:AB_2629736
BUV395-CD24	BD	Cat# 566221; RRID:AB_2739610
BUV496-CD21	BD	Cat# 750188; RRID:AB_2874390
BUV563-CD10	BD	Cat# 748731; RRID:AB_2873135
BUV661-CD71	BD	Cat# 750651; RRID:AB_2874779
BUV737-IgG	BD	Cat# 741858; RRID:AB_2871189
BUV805-IgM	BD	Cat# 748927; RRID:AB_2873330
Brilliant Violet™ 421-IL21R	BioLegend	Cat# 347809; RRID:AB_2561651
Pacific Blue™-CXCR3	BioLegend	Cat# 353724; RRID:AB_2561442
Brilliant Violet™ 570-CD19	BioLegend	Cat# 302236; RRID:AB_2563606
Brilliant Violet™ 605-TACI	BD	Cat# 744145; RRID:AB_2742031
Brilliant Violet™ 711-CD20	BD	Cat# 563126; RRID:AB_2313579
Brilliant Violet™ 785-IL21-R	BD	Cat# 742422; RRID:AB_2740773
PerCP/Cyanine5.5-IgD	BioLegend	Cat# 405709; RRID:AB_1575115
PE/Dazzle 594-BCMA	BioLegend	Cat# 357512; RRID:AB_2566531

(*Continued on next page*)

Continued		
REAGENT or RESOURCE	SOURCE	IDENTIFIER
PE/Cyanine5-CXCR4	BioLegend	Cat# 306508; RRID:AB_314614
PE/Cy7-CD138	BioLegend	Cat# 356514; RRID:AB_2562658
Alexa Fluor® 700-CD27	BioLegend	Cat# 302814; RRID:AB_493757
APC Cy7-BAFF-R	BioLegend	Cat# 316912; RRID:AB_2203681
V450-IFNg	BD	Cat# 560372; RRID:AB_1645595
Brilliant Violet™ 510-CD45RA	BioLegend	Cat# 304142; RRID:AB_2561947
Brilliant Violet™ 605-CD27	BioLegend	Cat# 302830; RRID:AB_2561450
Brilliant Violet™ 650-CD8	BioLegend	Cat# 344730; RRID:AB_2564510
Brilliant Violet™ 711-IL2	BioLegend	Cat# 500346; RRID:AB_2616639
FITC-CD40L	BioLegend	Cat# 310804; RRID:AB_314827
PerCP-CD3	BioLegend	Cat# 981016; RRID:AB_2876777
PE-IL21	BioLegend	Cat# 513004; RRID:AB_2249025
PE/Dazzle 594-CD107a	BioLegend	Cat# 328646; RRID:AB_2566115
PE/Cyanine7-CXCR5	BioLegend	Cat# 356924; RRID:AB_2562355
APC-TNFa	BioLegend	Cat# 502912; RRID:AB_315264
Alexa Fluor® 700-CD4	BioLegend	Cat# 317426; RRID:AB_571943
Bacterial and virus strains		
YF17D Stamaril	Sanofi Pasteur, Lyon, France	NA
YF17D-Venus	Dr. Charles M. Rice (Rockefeller University, NY, USA).	NA
Biological samples		
Sera, plasma and PBMC of YF17D vaccinees	This study	NA
Chemicals, peptides, and recombinant proteins		
Recombinant YF17D soluble envelope	Dr. G. Barba-Spaeth. Santos-Peral et al.[35]	NA
StrepTactin-HRP	Bio-Rad	Cat# 1610380
BD OptEIA TMB Substrate Reagent Set	BD	Cat# 555214; RRID:AB_2869044
Polyethylene Glycol 8000	Carl Roth	Cat# 5322-68-3
TrueNuclear Perm Buffer	BioLegend	Cat# 424401
Foxp3/Transcription Factor Staining Buffer Set	Invitrogen	Cat# 00-5521-00
Zombie UV	BioLegend	Cat# 423108
Fixable Viability Dye eFluor 780	Thermo Fisher	Cat# 65-0865-14
Fixable Far Red Dead Cell Stain	Thermo Fisher	Cat# L10120
Paraformaldehyde	Sigma Aldrich	Cat# 8.18708
Brefeldin A	BioLegend	Cat# 420601
Antigens for multiplex serology assay	In house, Mentzer et al.[51]	NA
Critical commercial assays		
Bioplex Multiplex Assay pro Human Cytokine	Bio-Rad	Customized order
Bioplex Multiplex Assay pro Human Chemokine	Bio-Rad	Customized order
Bioplex Multiplex Assay pro Human Inflammation	Bio-Rad	Customized order
Deposited data		
NA	NA	NA

(*Continued on next page*)

Continued

REAGENT or RESOURCE	SOURCE	IDENTIFIER
Experimental models: Cell lines		
Vero B4	ATCC	Cat# CCL81; RRID:CVCL_0059
Software and algorithms		
GraphPad Prism 8	GraphPad, La Jolla, CA, USA	https://www.graphpad.com; RRID: SCR_002798
R version 4.3.2	R Core Team 2017	https://www.r-project.org; RRID: SCR_001905
COMPASS	Lin et al.[36]	https://github.com/RGLab/COMPASS; RRID:SCR_001801
Gemma	Zhou et al.[52]	https://github.com/genetics-statistics/GEMMA
TYPE® HLA Sequencing Software	Thermofisher	Cat# 53999100
NGSengine	gendx	https://www.gendx.com/product_line/ngsengine/
Bioplex manager software	Bio-Rad	https://www.bio-rad.com/; RRID:SCR_014330
FlowJo	FlowJo	https://www.flowjo.com/; RRID: SCR_008520
BioRender	BioRender website	https://www.biorender.com/; RRID:SCR_018361

EXPERIMENTAL MODEL AND STUDY PARTICIPANT DETAILS

Cohort and samples

250 healthy young adults, naive to flavivirus infections and to Japanese encephalitis and yellow fever virus vaccine, were recruited at the Division of Infectious Diseases and Tropical Medicine (DIDTM) as well as the Department of Clinical Pharmacology, University Hospital, LMU Munich, Germany. Permission of the responsible institutional review board at the Medical Faculty of LMU had been granted prior to study initiation (IRB #86-16). Clinical cohort details are registered in the ISRCTN registry: 17974967.[49] Participants were recruited over five years (2015–2019), and all gave informed consent before receiving the subcutaneous YF17D vaccine (Stamaril; Sanofi Pasteur, Lyon, France). Samples were collected immediately before immunization and on days 3, 7, 14, and 28 post-vaccination. Race and socioeconomic status were not criteria for recruitment. None of the participants had a fever or acute illness symptoms at the time of vaccination. The biological sex at birth was noted according to both self-reported information and genetic analysis. The final cohort consisted of 169 females and 81 males, with a median age of 24 years (range: 19–47). The body mass index (BMI) was within the normal range (18.5–24.9) for 212 participants (84.8%).

With every blood draw an evaluation of immune parameters (immune cell counts, CRP and bulk IgG, IgM and IgA concentration) were measured at the Institute of Laboratory Medicine of the LMU University Hospital Munich. All measurement procedures were performed and controlled according to standardized protocols accredited according to DIN EN ISO 15189 and DIN EN ISO/IEC 17025 by trained laboratory personnel. Serum and plasma samples were stored at -80°C and heat-inactivated before the assays. PBMC samples were isolated manually from buffy coat following Ficoll-Paque PLUS (GE Healthcare, Sweden) density centrifugation and cryopreserved in heat-inactivated fetal calf serum (FCS) supplemented with 10% DMSO (Sigma-Aldrich) in liquid nitrogen. All assays started with the thawing of cryopreserved PBMC with an average recovery of 70%.

METHOD DETAILS

HLA typing

HLA typing was done at the Laboratory of Immunogenetics and Molecular Diagnostics, Department of Transfusion Medicine, Cell Therapeutic Agents and Hemostaseology, LMU University Hospital Munich, Munich, Germany. HLA typing was performed using DNA isolated from buffy coats obtained directly prior to vaccination and either Sanger or Next Generation Sequencing (NGS). Typing results were reported in a 2field resolution (Sanger on 3130xl Genetic Analyzer, Applied Biosystems, Foster City, USA) or a 3field resolution (NGS Ion Chef System and Ion personal genome machine, life technologies/Thermo Fisher, Waltham, USA). Sanger sequencing was realized by a home-made PCR amplification strategy of class II (exon 2–4). Sequence raw data were processed either by uType (Thermo Fisher, Waltham, USA) software (Sanger) or by NGSengine (NGS; GenDx, Utrecht, Netherlands) for HLA type creation.

Serological analysis

Baseline serum was evaluated for specific IgG responses to multiple antigens in a multiplex serology assay performed by the Infections and Cancer Epidemiology Division at the German Cancer Research Center in Heidelberg and described in detail by Mentzer et al.[51] Antigens used for serodiagnosis are summarised in Figure S1. Donors were categorized into positive or negative groups based on established pre-validated thresholds. In cases where two or more antigens were utilized, the determination of overall seropositivity or negativity was carried out as follows: for CMV infection, seropositivity was assigned to individuals showing sero-reactivity for a minimum of 2 out of 3 antigens. For EBV, individuals were considered seropositive if they tested positive for 2 out of 4 antigens. Regarding *Toxoplasma gondii*, either one of the two antigens and for *Mycoplasma genitalium*, overall positivity was determined by seropositivity to both antigens. Additionally, the study participants were categorized based on their previous vaccination status with the inactivated TBEV vaccine. Together with the self-reported vaccination status, TBEV pre-immunity was confirmed by a positive result for an in-house TBEV neutralization assay and by the presence of anti-TBEV IgG antibodies in an enzyme-linked immunosorbent assay (ELISA) utilizing TBEV-DIII as the antigen (reported in detail by Santos-Peral et al.[35]). Since the study was conducted in Bavaria, a German region where TBEV vaccine is recommended, a significant number of the cohort had received at least one dose of a TBEV vaccine before inclusion in the study (139 TBEV-IgG positive, 56-IgG TBEV negative, and 55 unknown).[35]

YF17D and YF17D-Venus virus production

The vaccine used in the study, Stamaril, was manufactured in France by Sanofi Pasteur and was given as a standard of care to all the study participants from 2015 to 2019. Stamaril contains a life-attenuated 17D-204 substrain. For *in vitro* assays, the YF17D virus was directly amplified from the Stamaril vaccine dose. YF17D variant YF17D-Venus plasmid was a generous gift from Charles M. Rice and Margaret MacDonald (The Rockefeller University, New York, USA). Virus stock production and purification was done as previously described.[53,54] In brief, the supernatant from infected Vero B4 cells was harvested 3–4 days post-infection, when a cytopathic effect became visible, and the cellular debris was homogenized using a douncer. Subsequently, the supernatant was mixed with 7% (w/v) polyethylene glycol 8000. The virus-PEG complexes were pelleted and resuspended in TNE buffer (20 mM Tris-HCl, pH 8, 150 mM NaCl, 2 mM EDTA) before further purification on a 30/60% sucrose cushion. The infectivity of the purified virus was determined by plaque assay and stored at -80°C.

Yellow fever neutralization

The neutralizing antibody titer was quantified by a Fluorescence Reduction Neutralization Test (FluoRNT) as previously described by Scheck et al.[54] The frequency of YF17D-Venus virus-infected Vero cells in the absence of vaccinee serum was set as 100% and the percentage of reduction was calculated for serial serum dilution steps. Neutralization curves were fitted by 4-parameter logistic regression using Prism 8 (GraphPad, La Jolla, CA, USA). 50% FluoRNT values were interpolated from the curves.

Cytokine measurement

Cytokine concentrations were measured in plasma samples at days 0, 3 and 7 using three panels (pro-Human Cytokine, pro-Human Chemokine, pro-Human Inflammation) of the Bioplex Multiplex Assay (Bio-Rad Laboratories, USA) according to the manufacturer's instructions. Data was subsequently analyzed using the Bio-plex Manager Software.

T cell re-stimulation with YF-17D virus

To measure antigen-specific T cells, cryopreserved PBMC were thawed and rested overnight at high density (5.10^6 cells per mL) in R10 medium at 37°C in a 5% CO2 humidified atmosphere. PBMC were stimulated at a concentration of 5 million cells/mL. Cells were then incubated with live YF17D virus (MOI 3) or with the equivalent volume of purified supernatant of uninfected cells (unstimulated control). PBMC were stimulated for 20 h and brefeldin A (BioLegend) was added for the last 4 h of stimulation.

Intracellular staining of *ex vivo* re-stimulated samples

The staining of the PBMC starts with the addition of anti-CD107a (clone H4A3, BD Biosciences) for the last 4 h of stimulation. After the stimulation, PBMCs were stained for viability (fixable Viability Dye eFluor 780, ThermoFischer) for 20 min on ice. Next, PBMC were blocked for 10 min with 10% human AB serum (Sigma) in FACS buffer (BSA 0.5%, 2 mM EDTA in PBS). Surface staining was done in blocking buffer with a combination of fluorochrome labeled anti-human $CD4^+$ (clone OKT4, BD BioLegend), CD3 (clone SK7, BD Biosciences), $CD8^+$ (clone SK1, BioLegend), CD27 (clone O323, BioLegend), CCR7 (clone G043H7, BioLegend) and CD45RA (clone HI100, BioLegend) markers. Intracellular cytokine staining (ICS) was performed following fixation and permeabilization with Foxp3/Transcription Factor Staining Buffer Set (Invitrogen) for 20 min at RT. Cells were stained for 45 min with anti-IFN-γ (clone B27, BD Biosciences), TNF-α (clone MAb11, BioLegend) IL-2 (clone MQ1-17H12, BD Biosciences), CD40L (Clone 24–31, BioLegend), IL21 (Clone 3A3-N2, BioLegend) antibodies in permeabilization buffer. Cytokine expression level obtained in the unstimulated control was subtracted in the analysis of YF17D-specific cytokine responses. Flow cytometric analyses were performed using Fortessa (BD Biosciences) and data was analyzed with Flow Jo (v.10) software. Gating strategy is shown in Figure S6.

COMPASS analysis

The functional score of the antigen-specific T cell response was estimated using COMPASS as previously described.[36] FS is defined as the proportion of antigen-specific subsets detected among all possible ones. Six parameters were included for $CD4^+$ responses: IL-2, TNFα, IFNγ, IL-21, CD107a, CD40L and four for $CD8^+$ responses: IL-2, TNFα, IFNγ, CD107a. The data was analyzed with COMPASSSimple package from Bioconductor with a minimum of 100.000 iterations and 8 repetitions (Figure S6).

Flow cytometry and phenotyping

Monocyte and DC immune phenotyping was performed in four batches with the same panel of antibodies and gating strategy described previously.[50] For T and B cell analysis, cryopreserved PBMCs were thawed and incubated with Live/Dead stain. Next, PBMC were stained with optimized concentrations of surface marker antibodies in the dark for 30 min at 37°C. Cells were then washed twice with FACS buffer. For the intranuclear staining, the cells were fixed/permeabilized with TrueNuclear Fixation Buffer (BioLegend) for 1h at room temperature and protected from light. The cells were washed twice in TrueNuclear Perm Buffer (BioLegend) and incubated with optimized concentrations of antibodies for 20 h at 4°C. Cells were washed twice in the True Nuclear Perm Buffer and fixed with 4% PFA for 10 min at room temperature. For B cell phenotyping the following antibodies were used: viability marker Zombie UV, CD24 (ML5) BUV395, CD21 (1048) BUV496, CD10 (HI10a) BUV563, CD71 (M-A712) BUV661, IgG (G18-145) BUV 737, IgM (UCH-B1) BUV805, CD267 (1A1-K21-M22) BV605, Ki67 (B56) BV650, antiCXCR3 (G025H7) Pacific Blue, IL21R (2G1-K12) BV421, CD19 (HIB19) BV570, CD20 (2H7) BV711, Tbet (4B10) FITC, CD3 (SK7) Spark Blue 550, IgD (IA6-2) PerCP/Cy5.5, BCMA (19F2) PE/Dazzle 594, CXCR4 (12G5) PE/Cy5, CD138 (MI15) PE/Cy7, CD27 (O323) Alexa Fluor 700, BAFF-R (11C1) APC/Cy7, and CD38 (HB-7) APC/Fire 810. And for T cell analysis viability marker Zombie UV (BioLegend), CD45RA (HI100) BUV395, CD16 (3G8) BUV496, CD127 (HIL76M21) BUV 563, CCR6 (11A9) BUV661, PD1 (EH12.1) BUV 737, ICOS (DX29) BUV805, CD56 (NCAM16.2) BV480, Ki67 (B56) BV650, CXCR3 (G025H7) BV605, $CD4^+$ (SK3) BV510, KLRG1 (2F1) BV711, Tbet (4B10) FITC, CXCR5 (J252D4) BV750, CD3 (SK7) Spark Blue 550, CD25 (BC96) BB700, TCF1 (7F11A10) PE, FoxP3 (206D) PE/Dazzle 594, CCR4 (L291H4) PE/Cy7, HLA-DR (L243) PE/Fire 810, $CD8^+$ (HIT8a) Alexa Fluor 700, CD95 (DX2) APC/Fire 750, CD38 (HB-7) APC/Fire 810, and EOMES (WD1928) PE/Cy 5.5. Samples were measured using the Cytek Aurora (Cytek Biosciences) with the recommended Cytek assay settings, where gains are automatically adjusted after each daily QC based on laser and detector performance to an optimal value, ensuring comparability between measurements. Cell populations were gated manually using FlowJo v10 (gating strategies Figure S12).

ELISA for the quantification of anti-YF antibodies in human serum. YF17D-specific antibodies in human serum samples were quantified with an in-house ELISA assay using recombinantly produced soluble E protein (kindly provided by Giovanna Barba-Spaeth) or 17D-virion as antigens. 1 μg/mL (sE) or 3.5 μg/mL (virion) were coated overnight in half-area plates (Corning) in sodium bicarbonate solution pH 9.5. After washing the plates with PBS tween 0.05%, plates were then blocked with 10% goat serum in PBS tween 0.05% before the addition of diluted serum samples. Following sample incubation, plates were thoroughly washed before the incubation with HRP-linked anti-human IgG or IgM. Next, TMB substrate was added after thorough washings for approximately 30 min and the reaction stopped with H2SO4 2N. A background control was carried for every sample dilution in wells without pre-coated antigens. Antibody titers were calculated as relative units to a standard curve obtained upon serial dilutions of serum from a YF-vaccinated donor.[55]

QUANTIFICATION AND STATISTICAL ANALYSIS

Multivariate linear regression analysis

Before linear model implementation data used as dependent variables was adequately transformed as follows. "0" values were replaced with half the value of the smallest value for each parameter and data was normalized using the BoxCox approximation (package "fpp"). The multivariate regression analysis was performed in R (V 4.3.1) using the lm function from "stats" package and, unless they were the variable in question, the model included as covariates CMV infection status, age, sex, year of study inclusion, and the analysis batch of the corresponding variable. When outcome-of-vaccination parameters were evaluated as dependent variables, TBEV vaccination status was included as a covariate. When the linear model was implemented for good/weak responders, data was corrected for batch effects as described below beforehand to avoid model overfitting. 95% confidence intervals were calculated using the "confint" function from "stats" package in R. Information retrieved from the model was re-transformed to the original units. The effect size is the slope (estimate) of the model and fold-changes were calculated as (intercept + estimate)/intercept. A Benjamini-Hochberg procedure was implemented to adjust *p-value*s for multiple hypothesis testing per analysis group.

Batch correction

Flow cytometry data was corrected for batch effects before clustering or plotting. Briefly, 0 values were replaced with half the value of the smallest value for each parameter. The data was normalized using the BoxCox approximation (package "fpp") before adjusting for batch effects using ComBat ("sva" package). Data was then retransformed back to the original units inverting the BoxCox transformation.

CellPress
OPEN ACCESS

Hybrid hierarchical k-means clustering

Data were scaled before clustering with the hybrid hierarchical k-means approach using the R function "hkmeans" from factoextra package. This approach combines hierarchical clustering with K-means clustering refinement. The optimal number of clusters was determined by the NbClust function (index = all). For this approach, we used hierarchical clustering with Ward's minimum variance method based on a similarity measure of Euclidean distance applied to normalized data. Heatmaps were created using the complex-Heatmap R package.

Genetic association analysis

HLA typing data was used to perform the genetic association analysis. The analysis was performed for the main vaccination outcomes ($CD4^+$ and $CD8^+$ functional score, yellow fever-specific IgM and IgG antibody titer, neutralizing antibody titer, and frequency antigen-specific $CD4^+$ and $CD8^+$ measured by ICS for CD40L + IFNγ+ and IFNγ+TNFα+) and categorical outcome (weak, good responders). The associations were mapped using a linear mixed model in GEMMA[52](Version 0.98.3). Continuous vaccination outcome values were inverse-normal transformed. HLA alleles with minor allele frequency (MAF) > 0.05 were used for the analysis. Covariates age, sex and CMV status were included in the model. Relatedness among the individuals was accounted for in the model by including a genetic relatedness matrix, estimated from matched SNP genotype data. Wald test is applied to tests if the estimated effect size is different than zero. Bonferroni multiple testing correction was applied to account for the number of independent outcomes and gene combinations tested.

Principal component analysis of baseline parameters

Baseline parameter data was imputed using the k-nearest-neighbour method (k = 5). Principal component analysis was run in R using the 'prcomp' package. Inspecting the scree plot, we estimated that the contributions of the PCs level off after the 4th PC. Associations of the first 4 PCs with metadata and outcome parameters were assessed using t-test for binary categorical variables, ANOVA for categorical variables with multiple levels and spearman correlation for continuous variables. Bonferroni correction was applied to account for the number of tests conducted for each analysis.

Other statistics

Boxplots show a horizontal line indicating the median and lower and upper hinges corresponding to the first and third quartiles. The lower and upper whiskers extend to 1.5x IQR (interquartile range) from the respective hinge. Determination of statistical significance included Chi-square test, Wilcoxon Rank-Sum test, and Spearman correlations. For radar plots, data was scaled, and Student's t test or ANOVA was used to test the significance. For YF-specific IgG radar plots, data was scaled separately for TBEV pre-vaccination groups. For linear models evaluating IgG vaccination outcome, TBEV was included as a covariate. The test used in each case is indicated for the corresponding panels in the figure captions and the statistical significance is shown as: ns (non-significant), $^{*}p \leq 0.05$, $^{**}p \leq 0.01$, $^{***}p \leq 0.001$, $^{****}p \leq 0.0001$.

ADDITIONAL RESOURCES

The cohort and study have been registered on "https://www.isrctn.com/ISRCTN17974967" ID:17974967.

Cell Reports Medicine, Volume *6*

Supplemental information

Basal T cell activation predicts yellow fever vaccine response independently of cytomegalovirus infection and sex-related immune variations

Antonio Santos-Peral, Magdalena Zaucha, Elena Nikolova, Ekin Yaman, Barbara Puzek, Elena Winheim, Sebastian Goresch, Magdalena K. Scheck, Lisa Lehmann, Frank Dahlstroem, Hadi Karimzadeh, Julia Thorn-Seshold, Shenzhi Jia, Fabian Luppa, Michael Pritsch, Julia Butt, Camila Metz-Zumaran, Giovanna Barba-Spaeth, Stefan Endres, Sarah Kim-Hellmuth, Tim Waterboer, Anne B. Krug, and Simon Rothenfusser

Pathogen family or species	*Type*	*Antigen*	*Cohort prevalence (n = 248)*[a]	*Male n = 80*	*Female n = 168*
Papillomaviridae	**HPV 1**	L1	70 (28.23 %)	15	55
	**HPV 4**	L1	34 (13.71 %)	5	29
	**HPV 8**	L1	26 (10.48 %)	5	21
Polyomaviridae	**JCPyV**	VP1	110 (44.35 %)	41	69
	**KIPyV**	VP1	163 (65.73 %)	56	107
	**WUPyV**	VP1	240 (96.77 %)	77	163
	**HPyV 6**	VP1	202 (81.45 %)	65	137
	**HPyV 7**	VP1	113 (45.56 %)	37	76
	**MCV**	VP1	178 (71.77 %)	60	118
Herpesviridae	**HHV 1 or HSV1**	gG	124 (50.00 %)	40	84
	**HHV 2 or HSV2**	mgG	17 (6.85 %)	5	12
	**HHV 3 or VZV**	gE gI	145 (58.47 %)	46	99
	**HHV 4 or EBV**		210 (84.68 %)	64	146
		Zebra	198 (79.84 %)	61	137
		EA D	157 (63.31 %)	46	111
		VCA p18	186 (75.00 %)	57	129
		EBNA	206 (83.06 %)	65	141
	**HHV 5 or CMV**		83 (33.47 %)	25	58
		pp150	91 (36.69 %)	27	64
		pp52	77 (31.05 %)	21	56
		pp28	87 (35.08 %)	26	61
	**HHV 6**		70 (28.23 %)	18	52
		IE1B	58 (23.39 %)	13	45
		IE1A	21 (8.47 %)	7	14
	**HHV 7**	U14	187 (75.40 %)	59	128
Toxoplasma gondii	**Tg**		37 (14.92 %)	11	26
		p22	7 (2.82 %)	2	5
		Sag 1	31 (12.50 %)	9	22
Chlamydia trachomatis	**Ct**	Pgp3	35 (14.11 %)	6	29
Mycoplasma genitalium	**Mg**		6 (2.42 %)	3	3
		MgPa	19 (7.66 %)	6	13
		rMgPa	22 (8.87 %)	10	12
Parvoviridae	**B19**	VP1	175 (70.56 %)	57	118

Figure S1. Prior infection status of the study cohort
Cohort prevalence of 20 common pathogen infections including viruses, bacteria, and protozoans.
[a]2 individuals were not available for serology analysis

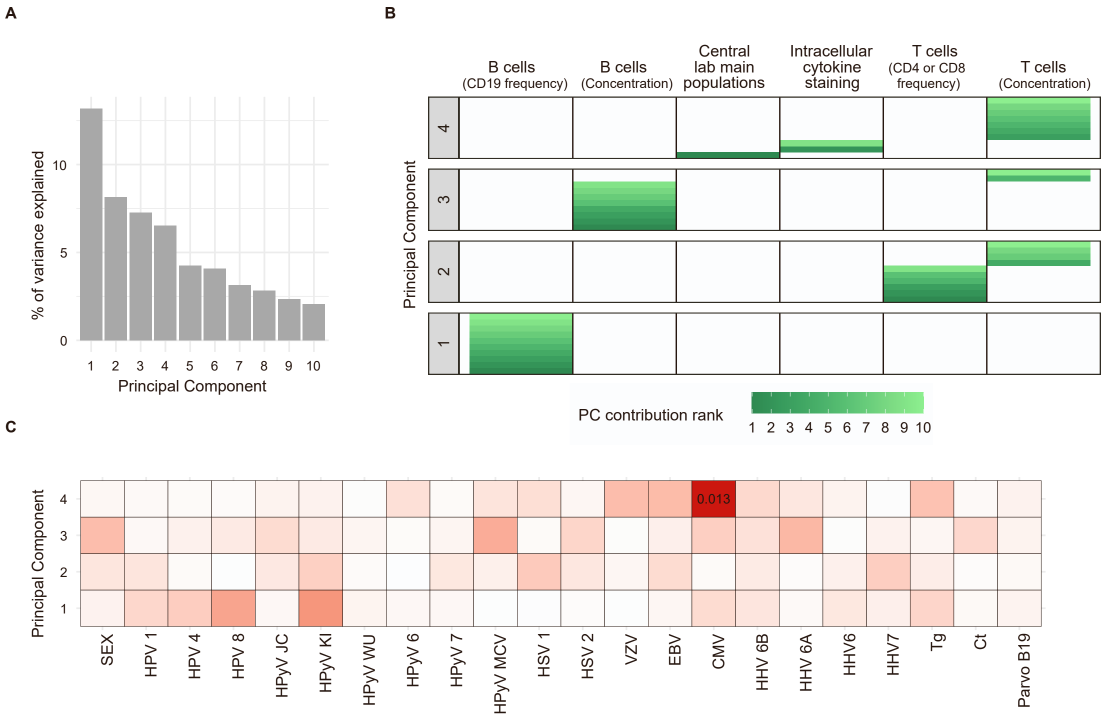

Figure S2. Principal component analysis of baseline parameters. Related to Figures 1 and 2 and Table S1.

A) Scree plot illustrating the proportion of variance explained by the top 10 principal components (PCs). B) Key contributors to the first four PCs, grouped by baseline parameter categories. PCs 1 and 3 are primarily influenced by B-cell-related measurements, while PCs 2 and 4 are largely driven by T-cell-related measurements. Notably, PC4 also captures variability related to lymphocyte counts. C) Association between baseline PCs and metadata categories. The mean of PC4 values differs significantly between CMV-positive and CMV-negative individuals (t-test, adj. p-value = 0.013). Baseline parameters are listed in supplementary table S1.

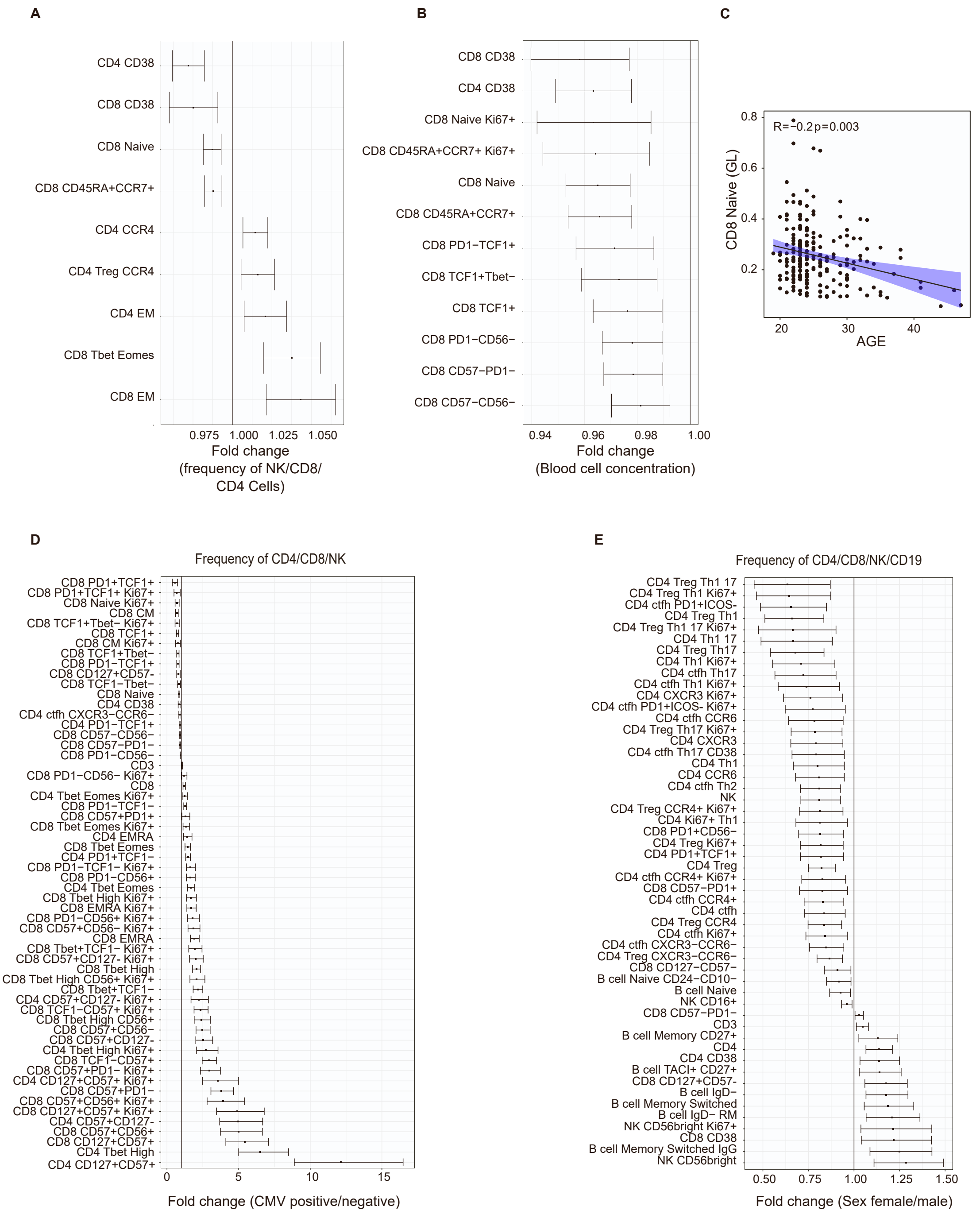

Figure S3. Effect of Age, CMV and Sex on baseline immune parameters. Related to Figures 1 and 2 and Table S2.

A-B) Effect of age on the frequency of circulating immune cells (A) or their concentration in blood (B) based on multiple regression analysis for CD8+, CD4+, B, and NK cell subsets. Fold changes corresponding to each additional year of age are displayed only for comparisons that meet the false discovery rate (FDR) threshold (< 0.1). Horizontal bars represent 95% confidence intervals (2.5-97.5%).
C) Spearman correlation between age and the concentration of naïve CD8+ T cells in blood (n = 238).
D-E) Effect of CMV infection (D) and sex (E) on the frequency of circulating immune cells, estimated using a multiple regression model for CD8+, CD4+, B, and NK cell parameters. Fold changes are reported only for significant comparisons (FDR < 0.1), with horizontal bars indicating the 2.5-97.5% confidence intervals. Sample sizes and linear model results can be seen in Tables S1 and S3. Phenotypic markers of T cell populations depicted in the figure are as follows: TSCM (CD45RA+CCR7+CD95+); Naïve (CD45RA+CCR7+CD95-); CM (CD45RA-CCR7+); EM (CD45RA-CCR7-); EM1 (CD45RA-CCR7-CD27+); EM2 (CD45RA-CCR7-CD27-); EMRA (CD45RA+CCR7-); pE (CD45RA+CCR7-CD27+); E (CD45RA+CCR7-CD27-); Effector (CD45RA+CD27-); cTfh (CXCR5+); Treg (FoxP3+CD25+CD127-); Th1 (CCR6-CXCR3+); Th17 (CCR6+CXCR3-); Th1-17 (CCR6+CXCR3+); Th2 (CCR6-CXCR3-CCR4+).
Phenotypic markers of B cell populations depicted in the figure are as follows: AM (CD27+CD21-); RM (CD27+CD21+); TLM (CD27-CD21-); IM (CD27-CD21+); plasmablasts (CD20-CD38+); Naïve (IgD+CD27-); Memory (CD27+); DN (IgD-CD27-); DN1 (DN, CXCR5+CD21+); DN2 (DN, CXCR5-CD21-); Memory switched (CD27+IgD-IgM-); Memory pre-switched (CD27+IgD-IgM+).

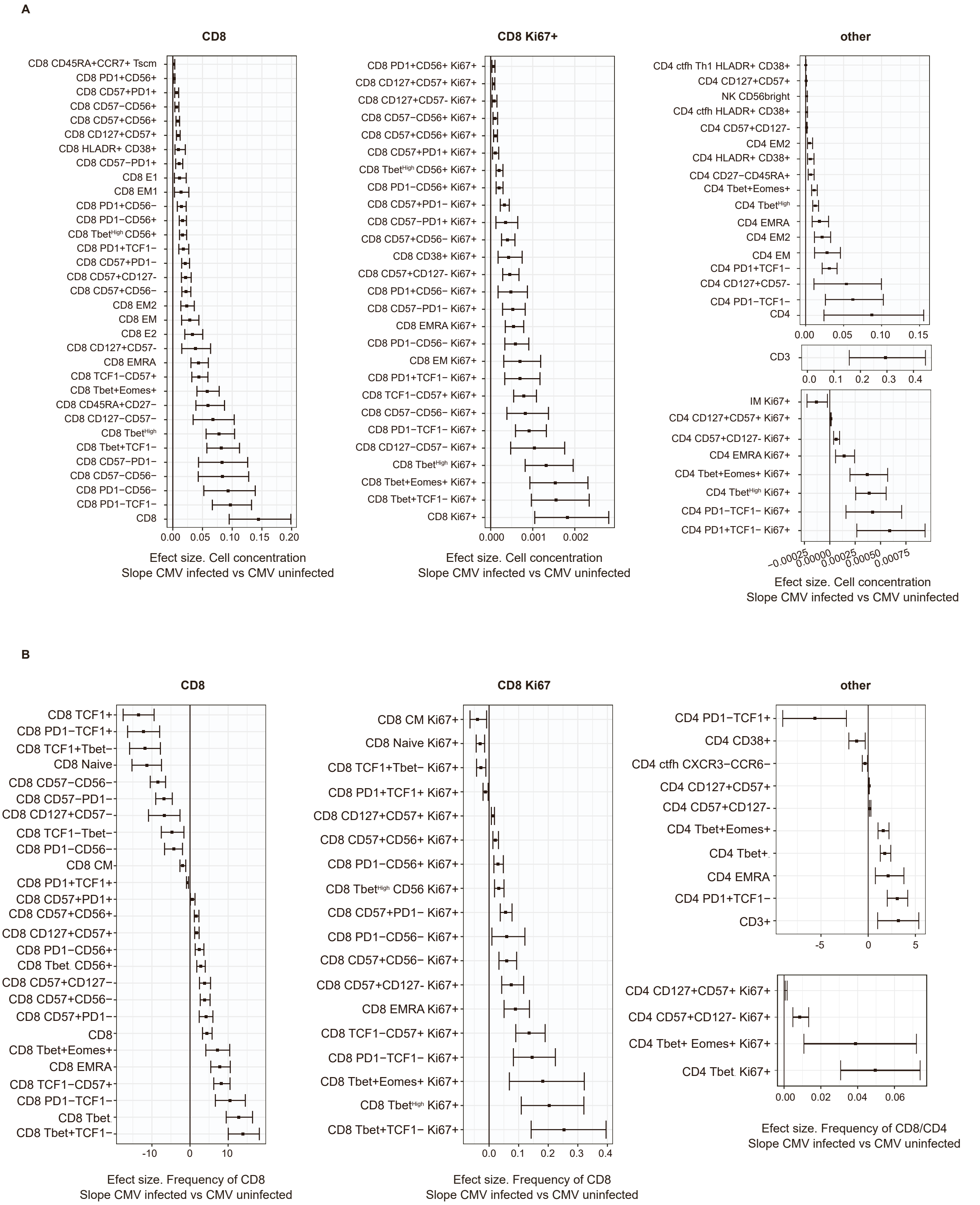

Figure S4. Effect size of CMV infection on blood cell concentrations and population frequencies. Related to Figure 1 and Table S2.

A) The estimated slope representing the difference in cell concentrations between CMV-infected and uninfected individuals calculated using a multiple regression model. B) The slope, representing the difference in CD8/CD4/NK frequencies between CMV-infected and uninfected individuals estimated by multiple regression. CD8, CD4 and CD3 values represent the difference in lymphocyte frequency.
Only comparisons passing a multiple testing correction (FDR<0.1) are shown. Horizontal bars indicate the 2.5-97.5 % confidence intervals. For sample sizes and further linear model results, see table S3.
Phenotypic markers of T cell populations depicted in the figure are as follows:: TSCM (CD45RA+CCR7+CD95+); CM (CD45RA-CCR7+); EM (CD45RA-CCR7-); EM1 (CD45RA-CCR7-CD27+); EM2 (CD45RA-CCR7-CD27-); EMRA (CD45RA+CCR7-); pE (CD45RA+CCR7-CD27+); E (CD45RA+CCR7-CD27-); Effector (CD45RA+CD27-); cTfh (CXCR5+).
Phenotypic markers of B cell populations depicted in the figure are as follows: AM (CD27+CD21-).

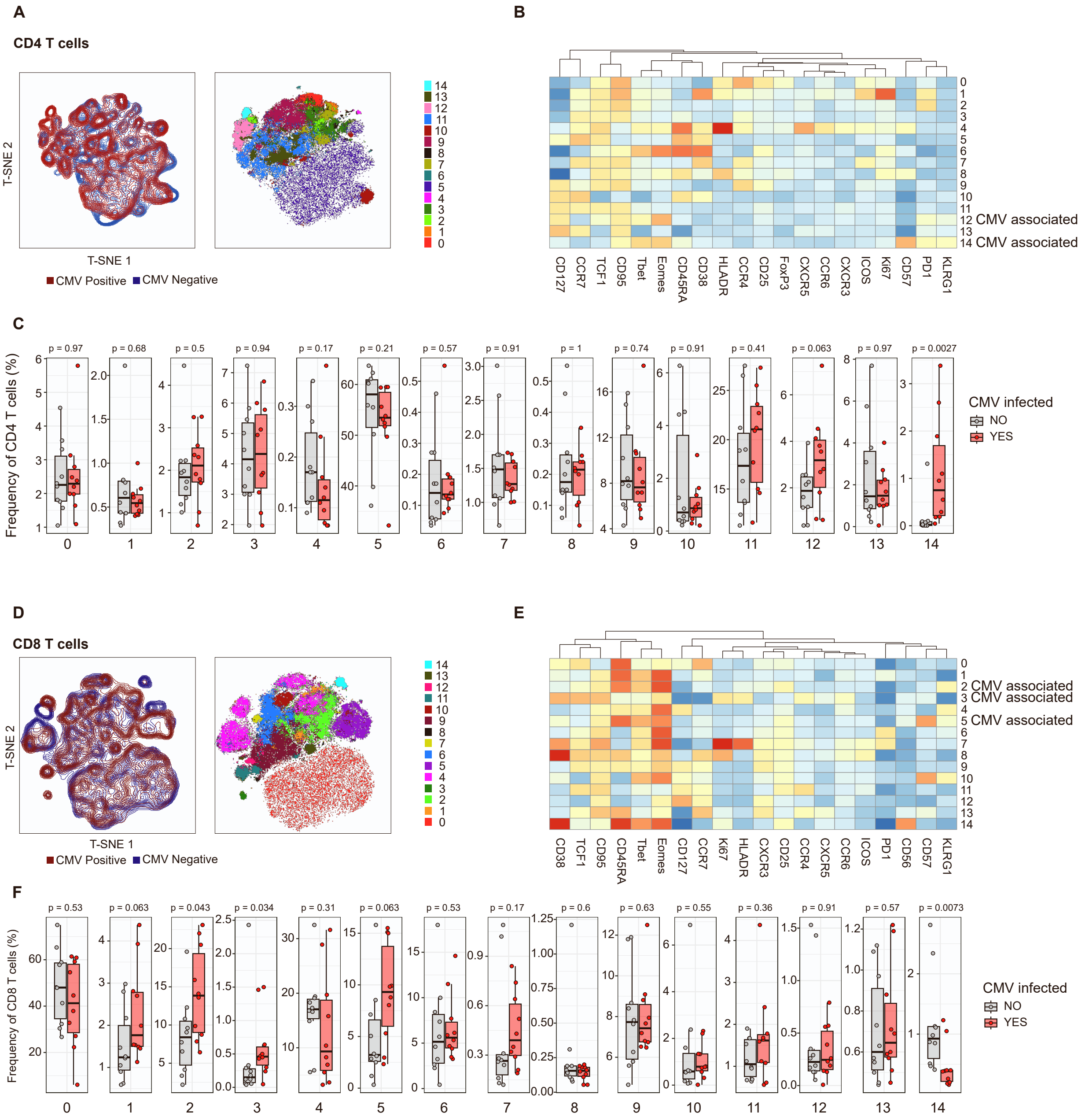

Figure S5. Dimension reduction and FlowSOM clustering of baseline CD4 and CD8 T cell populations. Related to Figure 1.

A) T-SNE dimensional reduction of CD4 T cells. The left panel visualizes the overlay of CMV-infected and uninfected groups on the T-SNE plot, while the right panel shows 14 clusters generated by FlowSOM clustering. B) Heatmap displaying the expression levels of 20 CD4 T cell markers across the 14 FlowSOM-identified clusters, with distinct expression patterns for each cluster. C) Boxplots illustrating the frequency of each CD4 T cell cluster for CMV-infected and uninfected individuals. D) T-SNE dimensional reduction of CD8 T cells. The left panel overlays CMV-infected and uninfected groups, and the right panel shows the 14 clusters identified by FlowSOM clustering. (E) Heatmap displaying the expression levels of 20 CD8 T cell markers across the 14 clusters, highlighting differential marker expression. (F) Boxplots displaying the frequency of each CD8 T cell cluster for CMV-infected and uninfected individuals.

The analysis was conducted on a cohort of 20 individuals (10 CMV-positive and 10 CMV-negative). For each individual, the analysis was restricted to 20,000 CD4 T cells and 12,900 CD8 T cells. Boxplots show a horizontal line indicating the median and lower and upper hinges corresponding to the first and third quartiles. The lower and upper whiskers extend to 1.5x IQR from the respective hinge. Significance in panels C and F was assessed using the Wilcoxon rank-sum test.

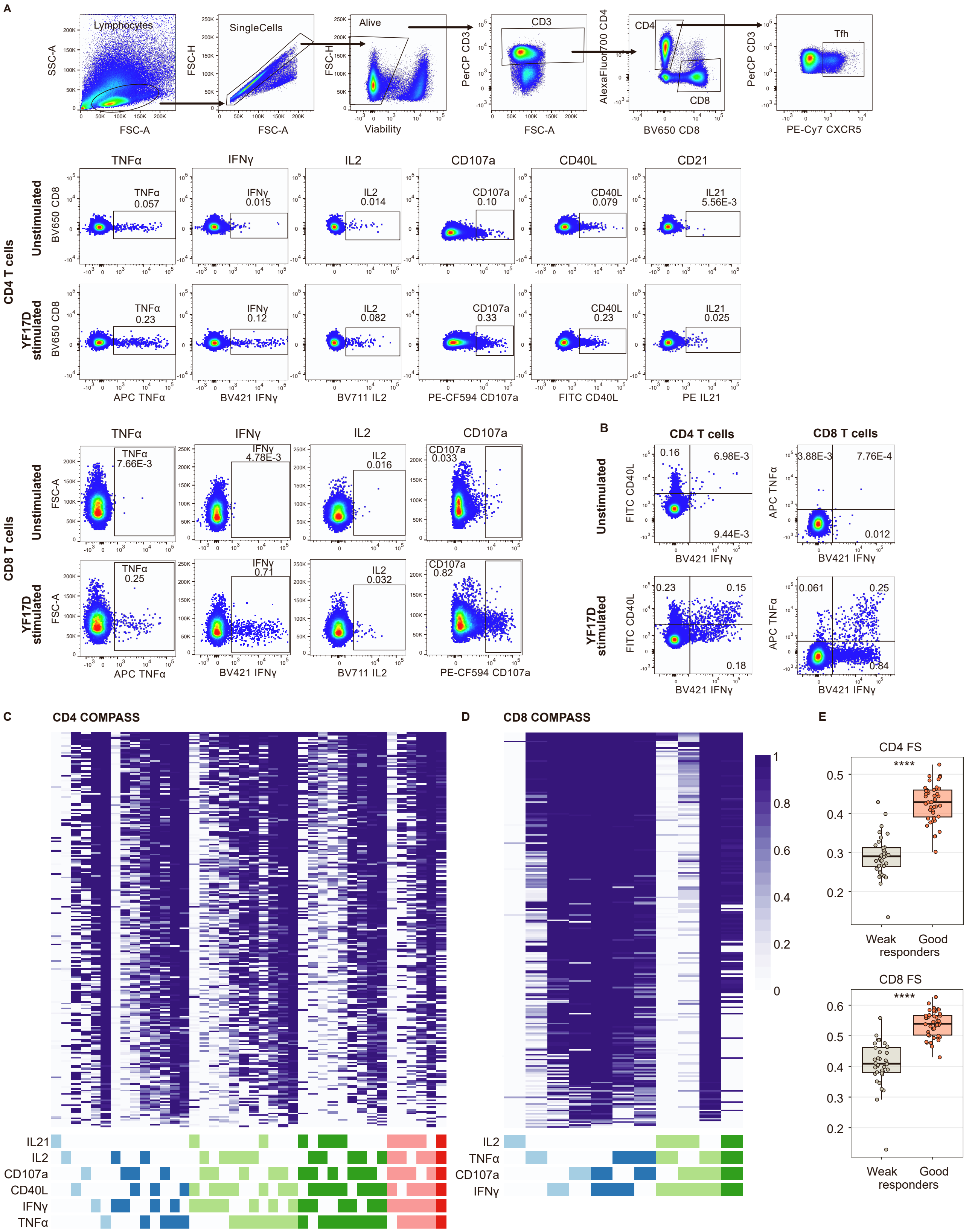

Figure S6. YF17D ex vivo restimulation and Combinatorial Polyfunctionality Analysis of Single Cells (COMPASS) of the antigen-specific CD4 and CD8 T cell response on day 28 pv. Related to Figure 3 and to STAR Methods, intracellular staining of ex vivo re-stimulated samples.

A) Gating strategy for intracellular cytokine staining for unstimulated and YF17D re-stimulated samples. B) Representative examples for the quantification of antigen-specific CD8 and CD4 T cells. C) Heatmap of the posterior probability of antigen-specificity for 64 functional CD4 subpopulations (n = 187 individuals) D) Heatmap of the posterior probability of antigen-specificity for 16 functional CD8 subpopulations (n = 187 individuals) E) Functionality score of the CD4 and CD8 T cell response for good (n = 44) and weak (n = 36) vaccine responders as defined in figure 4 A/B.

Functional subpopulations that lacked antigen-specificity were excluded from visualization in A and B. Boxplots show a horizontal line indicating the median and lower and upper hinges corresponding to the first and third quartiles. The lower and upper whiskers extend to 1.5x IQR from the respective hinge. Statistical significance for E was calculated with a Wilcoxon–Mann–Whitney test and depicted as **** p<0.0001.

A **TBEV pre-vaccination**

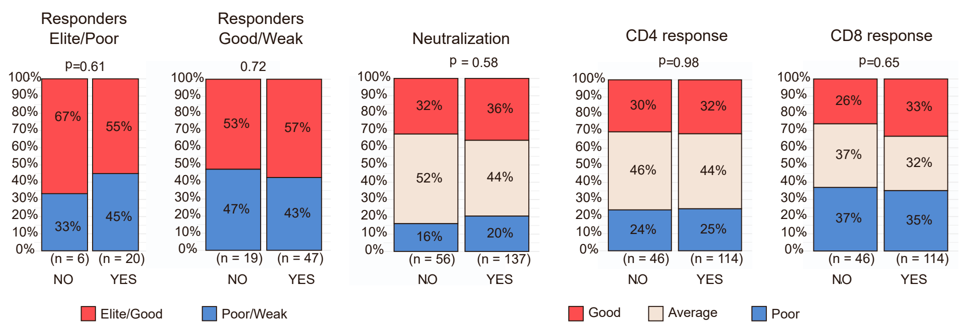

Figure S7. Categorical classification across vaccine response groups of individuals with or without a previous TBEV vaccination. Related to Figure 4.

TBEV-pre-vaccinated and TBEV-naive individuals were allocated to the vaccine response groups defined in Figure 4 A. Statistical significance was evaluated with a Chi-square test. Sample size is indicated below each comparison.

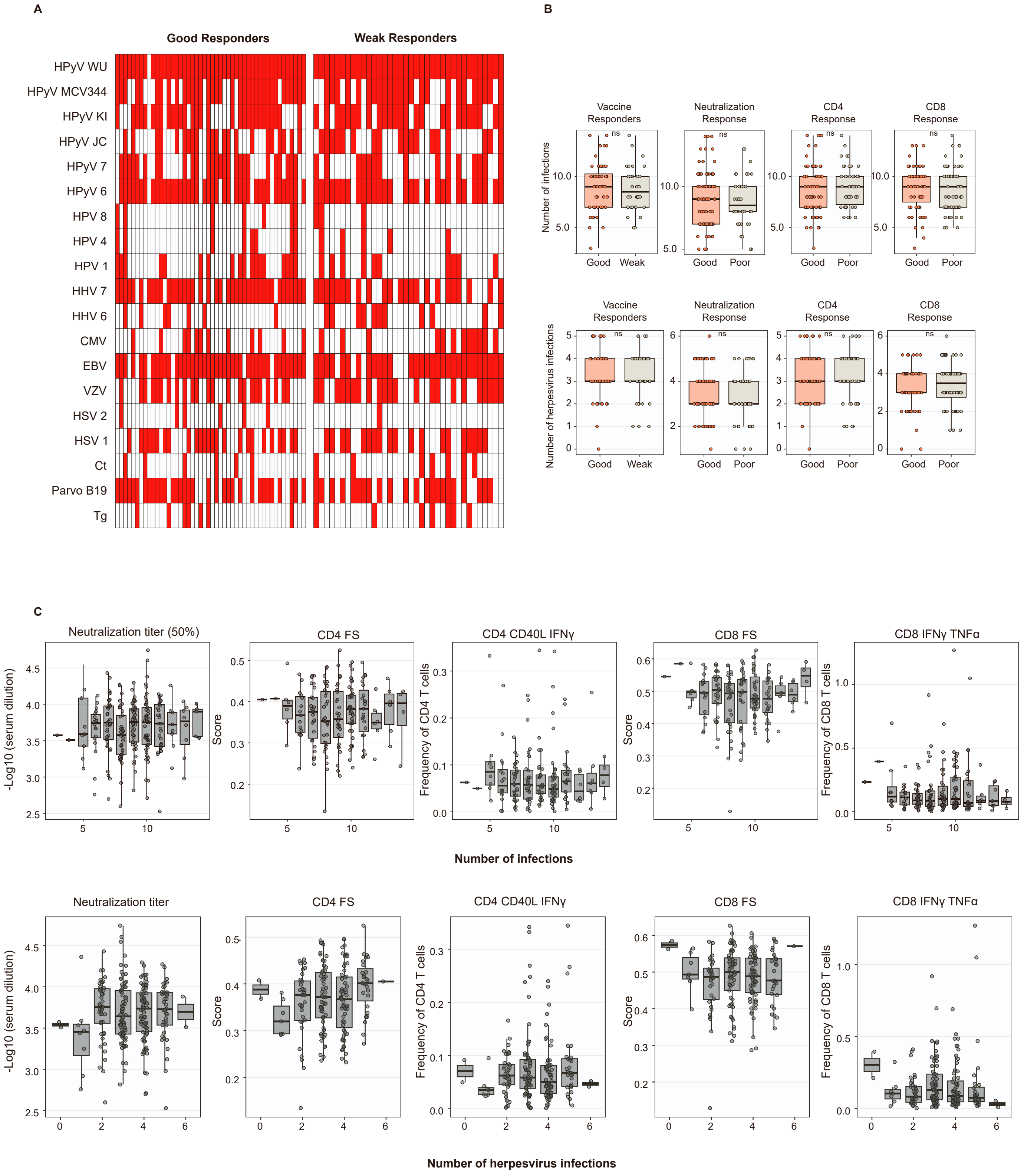

Figure S8. No cumulative effect of prior infections on the YF17D vaccine response. Related to Figures 3 and 4.

A) Depiction of the number of past infections for good and weak responders. Each column corresponds to an individual participant, with red indicating a positive infection history and white indicating a negative infection history for each assessed pathogen. B) Boxplots illustrating the distribution of the total number of infections (top panels) and herpesvirus infections (bottom panels) across good (n = 49) and weak (n = 37) responders, as well as good and poor neutralization groups (n = 90 and n = 48, respectively), and elite/good and poor CD4 (n =67 and n = 51, respectively) and CD8 responses (n = 60 and n = 73, respectively) as categorized in Figure 4A. C) Boxplots displaying the magnitude of selected vaccination endpoints for the study participants stratified by the number of prior infections. Boxplots show a horizontal line indicating the median and lower and upper hinges corresponding to the first and third quartiles. The lower and upper whiskers extend to 1.5x IQR from the respective hinge. Statistical significance was calculated with a Wilcoxon–Mann–Whitney test for B and C with the designation: ns (non-significant), *p ≤ 0.05, **p ≤ 0.01, ***p ≤ 0.001, ****p ≤ 0.0001.

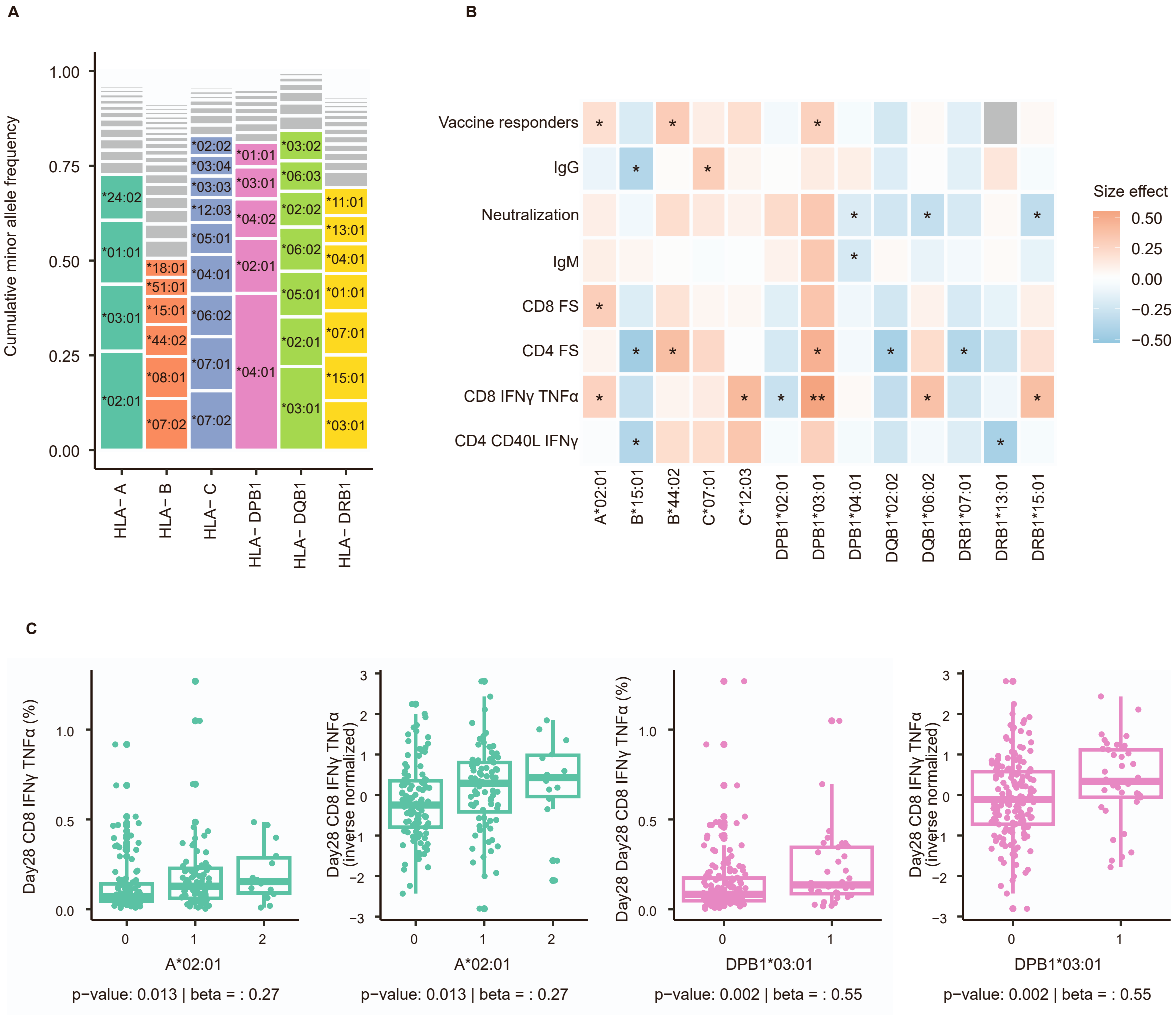

Figure S9. Association analysis of HLA alleles and vaccination outcomes. Related to Figures 4 and 5.

A) Frequency of HLA alleles for the 6 assessed HLA genes. Alleles with minor allele frequency < 0.05 were excluded from the analysis and are shown in grey. B) Association analysis results are shown for alleles with statistical evidence of association (p-value < 0.05) for at least one of the vaccination outcomes. The color gradient reflects the effect size and direction of the association, with statistical significance indicated by symbols (* = p ≤ 0.05, ** = p ≤ 0.01). Grey fields indicate combinations of alleles and outcomes that were not tested due to minor allele frequency threshold. C) Individual-level data for HLA-A*02:01 and for HLA-DPB1*03:01 grouped based on the number of gene copies (0, 1 or 2). For each allele, both the raw outcome counts, and the transformed outcome counts are shown. Boxplots show a horizontal line indicating the median and lower and upper hinges corresponding to the first and third quartiles. The lower and upper whiskers extend to 1.5x IQR from the respective hinge. Statistical significance in C was estimated with a Wilcoxon rank sum test.

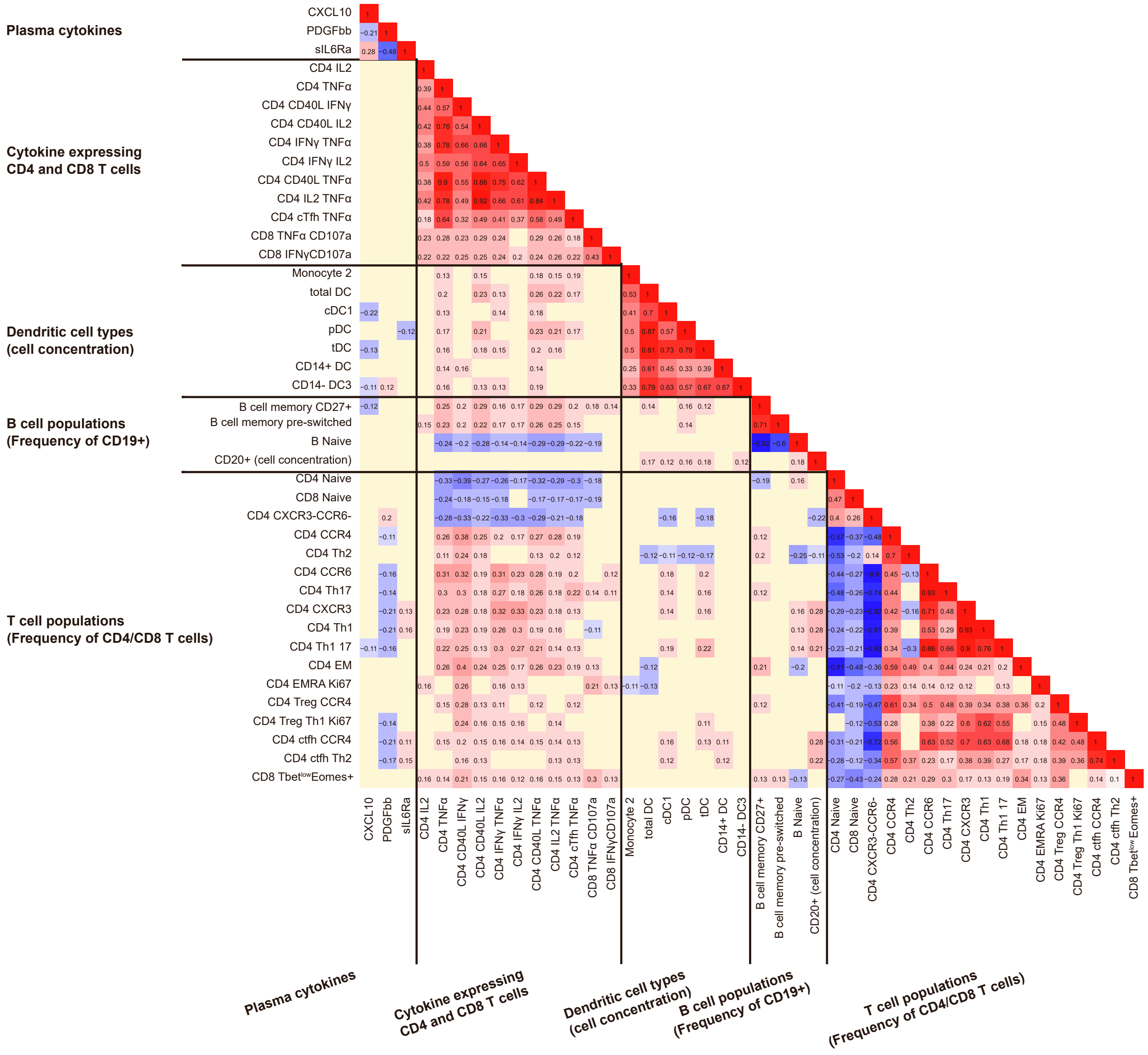

Figure S10. Spearman correlation of baseline parameters. Related to Figures 6 and 7.

This heatmap displays the Spearman correlation coefficients for statistically significant associations between baseline parameters (p-value < 0.05). The color gradient represents the magnitude and direction of the correlation, with positive correlations shown in the red spectrum and negative correlations in blue. Only significant correlations are shown, and the intensity of the color reflects the correlation spearman coefficient.

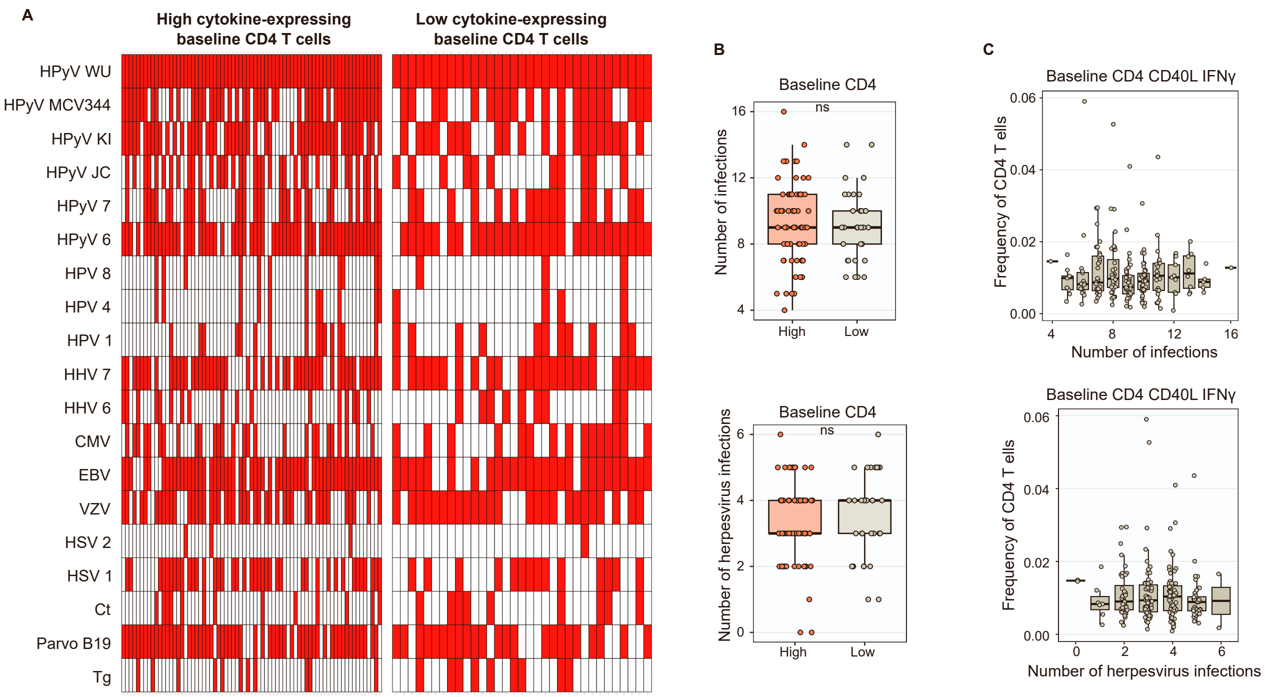

Figure S11. The cumulative number of prior infections is not associated with baseline cytokine-expressing CD4 T cells. Related to Figures 6 and 7.

A) Depiction of the number of past infections for individuals grouped by high or low levels of cytokine-expressing CD4 T cells as defined in Figure 6A. Each column represents an individual, with red indicating a positive infection history and white indicating a negative infection history for each pathogen assessed. B) Boxplots illustrating the distribution of the total number of infections (top panels) and herpesvirus infections (bottom panels) across the different baseline CD4 T cell clusters defined in Figure 6A. C) Boxplots illustrating the frequency of CD40L+ IFNγ+ CD4 T cells for the study participants stratified by the number of infections. Boxplots show a horizontal line indicating the median and lower and upper hinges corresponding to the first and third quartiles. The lower and upper whiskers extend to 1.5x IQR from the respective hinge. Statistical significance was calculated with a Wilcoxon–Mann–Whitney test for B and C with the designation: ns (non-significant), $*p \leq 0.05$, $**p \leq 0.01$, $***p \leq 0.001$, $****p \leq 0.0001$.

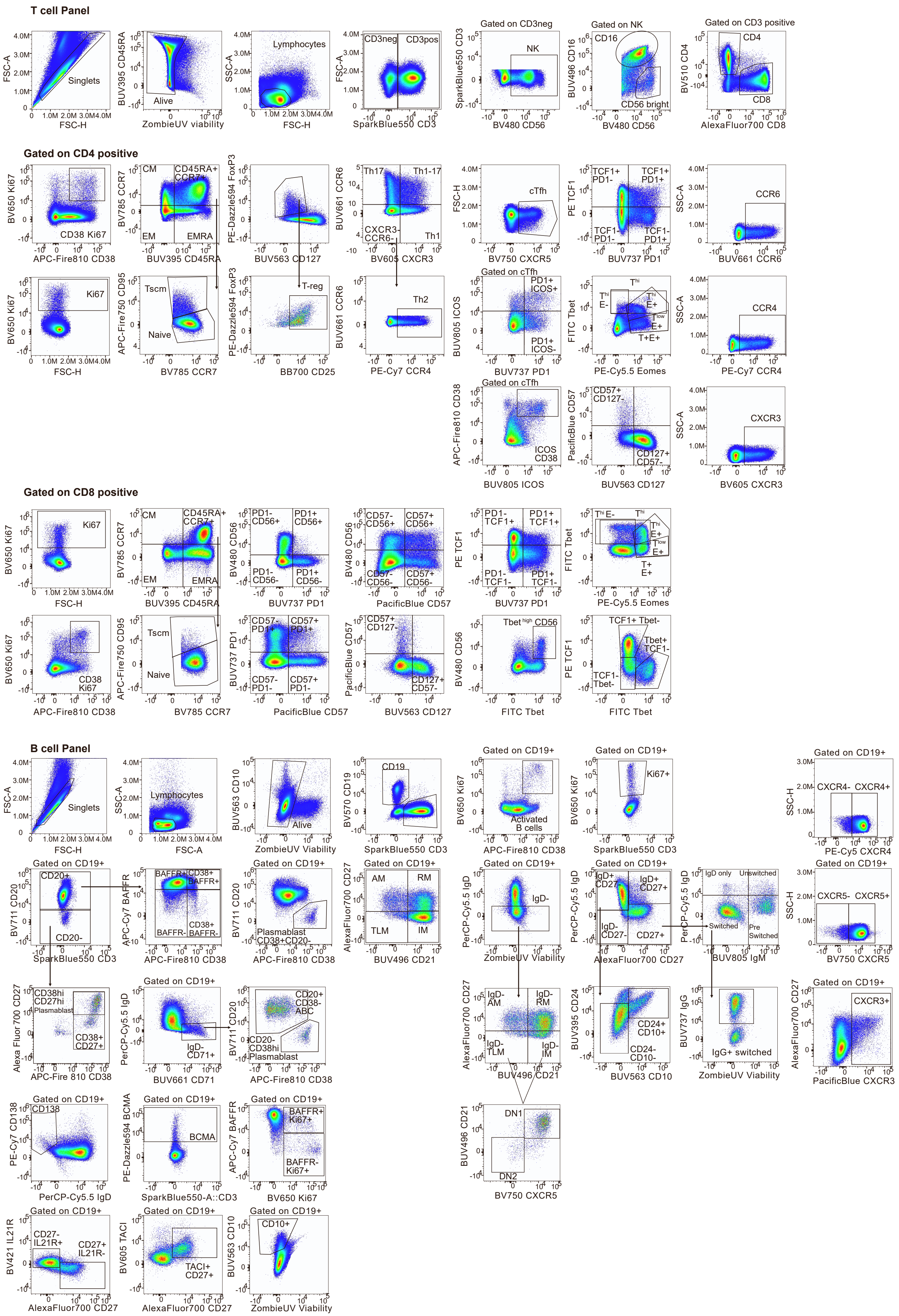

Figure S12. Gating strategy for T cell (top) and B cell (bottom) parameters. Related to Star Methods, Flow cytometry and phenotyping
